# Installing amino acids and peptides on *N*-heterocycles under visible-light assistance

**DOI:** 10.1038/srep20068

**Published:** 2016-02-02

**Authors:** Yunhe Jin, Min Jiang, Hui Wang, Hua Fu

**Affiliations:** 1 Key Laboratory of Bioorganic Phosphorus Chemistry and Chemical Biology (Ministry of Education), Department of Chemistry, Tsinghua University, Beijing 100084, P.R. China

## Abstract

Readily available natural α-amino acids are one of nature’s most attractive and versatile building blocks in synthesis of natural products and biomolecules. Peptides and *N*-heterocycles exhibit various biological and pharmaceutical functions. Conjugation of amino acids or peptides with *N*-heterocycles provides boundless potentiality for screening and discovery of diverse biologically active molecules. However, it is a great challenge to install amino acids or peptides on *N*-heterocycles through formation of carbon-carbon bonds under mild conditions. In this article, eighteen *N*-protected α-amino acids and three peptides were well assembled on phenanthridine derivatives via couplings of *N*-protected α-amino acid and peptide active esters with substituted 2-isocyanobiphenyls at room temperature under visible-light assistance. Furthermore, *N*-Boc-proline residue was successfully conjugated with oxindole derivatives using similar procedures. The simple protocol, mild reaction conditions, fast reaction, and high efficiency of this method make it an important strategy for synthesis of diverse molecules containing amino acid and peptide fragments.

Amino acids are one of nature’s most attractive and versatile building blocks in the synthesis of natural products and biomolecules^1^, and peptides are of great importance in present day drug discovery programs^2^. On the other hand, *N*-heterocycles are ubiquitous in natural products and biologically active molecules^3^, and they have been assigned as privileged structures in drug discovery because *N*-heterocyclic moieties often exhibit improved solubility and can facilitate salt formation property, both of which are important for oral absorption and bioavailability^4^,^5^,^6^. Conjugations of amino acids or peptides with *N*-heterocyclic compounds provide great opportunity for screening and discovery of diverse biologically active substances. Among the previous strategies available, common conjugations are usually performed through formation of amides (Fig. 1a), carbon-heteroatom bonds (Fig. 1b). The conjugations via the formation C-C bonds have been developed by using olefin metathesis^7^ and transition metal-catalyzed cross-coupling^8^ strategies. However, connections by C-C bonds still are a great and pressing challenge for chemists because of inherent structural characteristics of amino acids and peptides. On the other hand, the transition metal-catalyzed decarboxylative strategy for the formation of C-C bonds has provided some valuable reactions in organic synthesis^9^,^10^,^11^,^12^, such as Heck-type reactions^13^,^14^, allylations^15^, redox-neutral cross-coupling reactions^16^,^17^, and oxidative arylations^18^,^19^. However, the reactions usually need high temperatures and the stronger bases, which are intolerant for amino acids and peptides. Recently, visible light photoredox catalysis has attracted much attention, and it has emerged as a powerful activation protocol in new chemical transformations^20^,^21^,^22^,^23^,^24^,^25^,^26^,^27^. Furthermore, some decarboxylative couplings to the formation C-C bonds have been developed^28^,^29^,^30^,^31^,^32^,^33^,^34^,^35^,^36^,^37^,^38^,^39^. To the best of our knowledge, conjugation between amino acids or peptides and *N*-heterocycles is limited via formation of C-C bond under mild conditions and visible-light assistance^38^. Herein, we report an efficient installation of amino acids and peptides on phenanthridine and oxindole derivatives at room temperature under visible-light assistance (Fig. 1c).

## Results and Discussion

Since 2-isocyanobiphenyls are effective radical acceptors^40^,^41^,^42^, we first selected them as the partners of *N*-protected amino acid active esters under the visible-light photoredox catalysis. As shown in Table 1, our investigation for optimized conditions began by using model reaction of *N*-Boc-Pro-OPht (Pht = phthalimide) (**1r**) with isonitrile **2a**. The reaction was performed by using 1.0 mol% [Ru(bpy)_3_]Cl_2_ as the photoredox catalyst, one equiv of diisopropylethylamine (DIPEA) (relative to amount of **2a**) as the reductant, DMF as the solvent at room temperature under argon atmosphere for 2 h (entry 1), and **3r** was produced in 86% conversion yield (determined by ^1^H NMR using trichloroethylene as the internal standard). When 0.4 equivalent of DIPEA was used, yield decreased (entry 2), but addition of 1.2 equiv of K_2_CO_3_ as the base greatly promoted their reactivity (entry 3). Yields declined in the presence of 0.2 equiv of DIPEA (entry 4) or in the absence of DIPEA (entry 5). Other tertiary amines were screened (entries 6 and 7), and they were inferior to DIPEA. We investigated Na_2_CO_3_, Cs_2_CO_3_ and NaHCO_3_ as the bases (entries 8–10), and the results showed that K_2_CO_3_ was a suitable base (compare entries 3, 8–10). Effect of solvents was explored, and DMF provided the best result (compare entries 3, 11–13). The reaction in aqueous media (DMF/H_2_O = 9:1) was also attempted, and a 92% yield was provided (entry 14). When [*fac*-Ir(ppy)_3_] was used as the photoredox catalyst, only 79% conversion yield was found (entry 15). No reaction was observed when the system was exposed in air (entry 16). The reaction was performed well under irradiation of blue LED (entry 17). The reaction did not work in the absence of light (entry 18).

**Figure 2:**
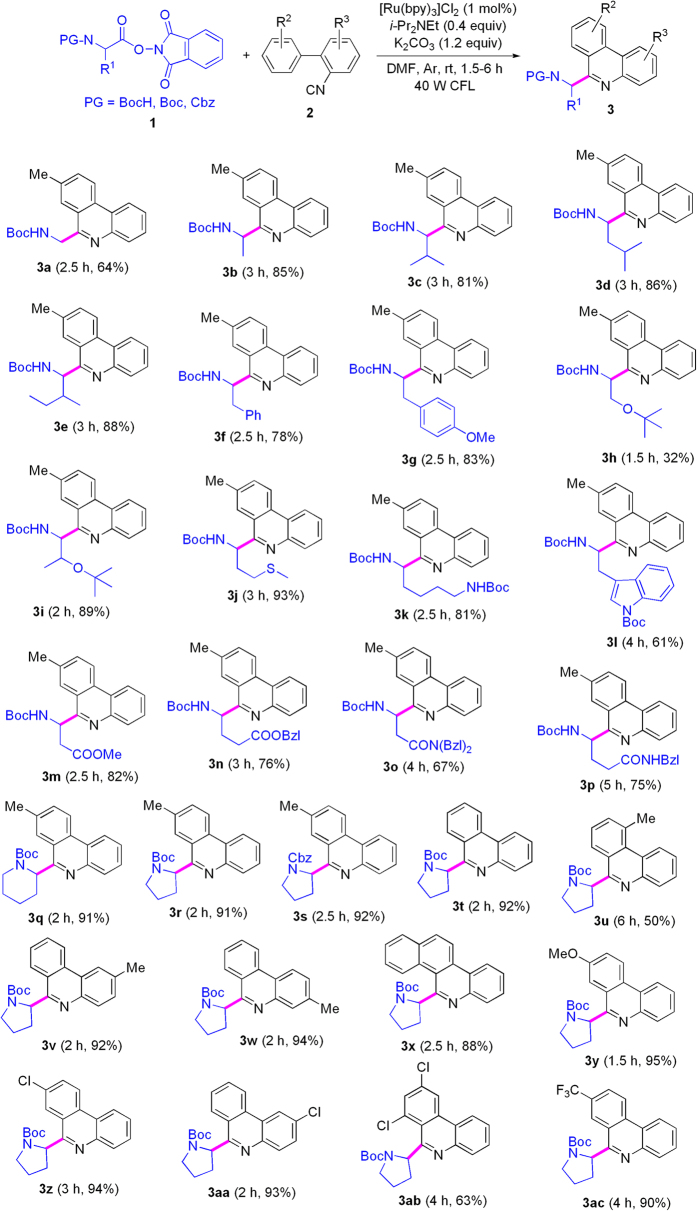
Substrate scope on conjugations of *N*-protected amino acids and phenanthridines*. ^*^Reaction conditions: under Ar atmosphere and irradiation of visible light, *N*-protected amino acid-OPht (**1**) (0.45 mmol for synthesis of 3**a–p**; 0.30 mmol for synthesis of the others), substituted 2-isocyanobiphenyl (**2**) (0.15 mmol), [Ru(bpy)_3_]Cl_2_ (1.5 μmol), DIPEA (0.06 mmol), K_2_CO_3_ (0.18 mmol), DMF (2.0 mL), temperature (rt, ~25 ^o^C), time (1.5–6 h) in a sealed Schlenk tube. ^†^Isolated yield. Boc = *tert*-butyloxycarbonyl. Bzl = benzyl. Cbz = benzyloxycarbonyl.

After getting the optimized conditions under visible-light assistance, we investigated the substrate scope of this reaction by testing decarboxylative couplings of various *N*-protected amino acid active esters (**1**) with substituted 2-isocyanobiphenyls (**2**). As shown in Figure 2, active esters of six neutral amino acids including glycine, alanine, valine, leucine, isoleucine and phenylalanine provided high yields (see **3a**–**f**). *N,O*-Protected amino acid active esters with hydroxyl on the side chains were tested (see **3g**–**i**), *N*-Boc-Ser(OBu^*t*^)-OPht gave lower yield for occurrence of unknown by-products (see **3h**), and *N*-Boc-Tyr(OMe)-OPht and *N*-Boc-Thr(OBu^*t*^)-OPht provided satisfactory results (see **3g** and **3i**). *N*-Boc-Met-OPht was a good substrate, and it afforded the target product **3j** in 93% yield. *N, N’*-Bis(Boc)-protected Lys-OPht and Trp-OPht displayed high reactivity (see **3k** and **3l**). Active esters of two acidic amino acids (aspartic acid and glutamic acid) donated good yields after their carboxyls on the side chains were esterized with methanol or phenylmethanol (see **3m** and **3n**). *N, N’*-Protected asparagine and glutamine active esters afforded **3o** and **3p** in 67 and 75% yields, respectively. *N*-Boc pipecolinic acid active ester was used as the substrate, and it showed good result (see **3q**). We attempted another *N*-protective group, benzyloxycarbonyl (Cbz), and *N*-Cbz-Pro-OPht exhibited similar reactivity to *N*-Boc-Pro-OPht (see **3r** and **3s**). Unfortunately, active esters of three natural amino acids including cysteine, histidine and arginine gave some by-products because of side reaction on side chains of the amino acids under the present photoredox conditions. We also explored the scope of substituted 2-isocyanobiphenyls, and isonitriles with steric hindrance provided lower yields (see **3u** and **3ab**). The visible-light photoredox decarboxylicative couplings showed tolerance of some functional groups including amides, ether (see **3g**, **3h**, **3i** and **3y**), thioether (**3j**), esters (see **3m** and **3n**), C-Cl bond (see **3z**, **3aa** and **3ab**), and CF_3_ (see **3ac**). It is worthwhile to note that the obtained products contain amino acid residues, and further derivatization is an easy task after the protective groups on the amino acid residues are removed. Therefore, the results above provide opportunity for construction of diverse molecules. In addition, phenanthridines are found in a wide variety of naturally occurring alkaloids^43^,^44^,^45^ and display diverse biological and pharmaceutical activities^46^,^47^. The present method affords a convenient, efficient and practical protocol for synthesis of phenanthridines.

**Figure 3:**
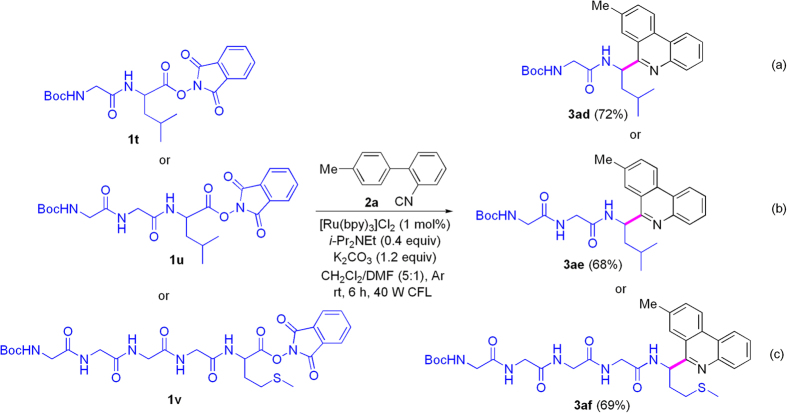
Conjugations of *N*-Boc peptides and 8-methylphenanthridine under visible-light assistance.

Inspired by the excellent results above, we extended scope of substrates to *N*-Boc-peptide active esters. As shown in Fig. 3, reaction of dipeptide derivative Boc-Gly-Leu-OPht (**1t**) with **2a** provided the target product (**3ad**) in 72% yield under photoredox catalysis in mixed solvent of CH_2_Cl_2_ and DMF (2.0 mL, CH_2_Cl_2_/DMF = 5:1) (note: the active ester was not dissolved well in complete DMF) (Fig. 3a). Further, we attempted decarboxylative coupling of tripeptide Boc-Gly-Gly-Leu-OPht (**1u**) and pentapeptide Boc-Gly-Gly-Gly-Gly-Met-OPht (**1v**) with **2a** under the same conditions, and **3ae** and **3af** were obtained in 68% and 69% yields, respectively (Fig. 3b,c). The results above exhibited that Boc-protected peptide active esters also were effective radical precursors for the photoredox catalysis.

**Figure 4:**
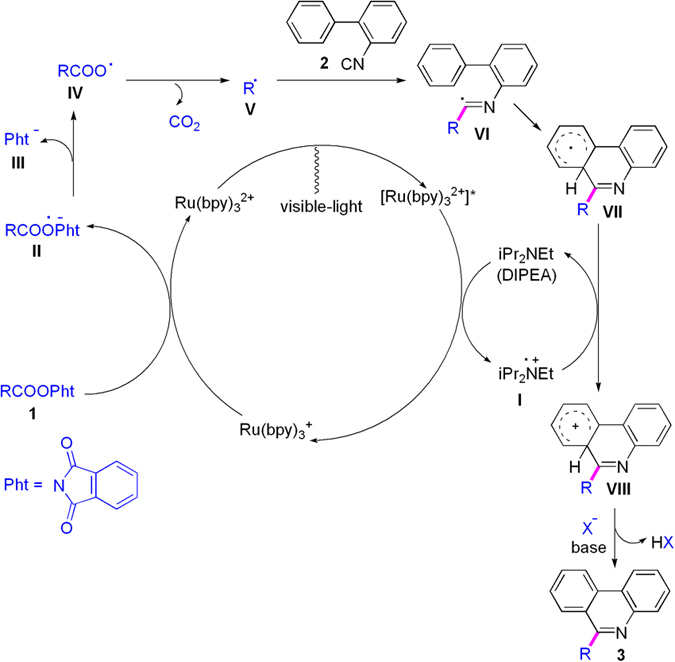
A plausible mechanism on installing *N*-protected amino acids and peptides on phenanthridines under visible-light assistance.

To explore the mechanism on this conjugation, a radical-trapping agent, 2,2,6,6-tetramethyl-1-piperidinyloxy (TEMPO), was added to the reaction system of *N*-Boc-Pro-OPht (**1r**) and isonitrile **2a**, and the reaction was completely inhibited, which shows that a free-radical intermediate process can be involved in the reaction. Therefore, a plausible mechanism on the visible-light photoredox synthesis of phenanthridines is suggested in Fig. 4 according to the results above and the previous references^20^,^21^,^22^,^23^,^24^,^25^,^26^,^27^,^28^,^29^,^30^,^31^,^32^,^33^,^34^,^35^,^36^,^37^,^38^,^39^. Irradiation of Ru(bpy)_3_^2+^ with visible light gives the excited-state [Ru (bpy)_3_^2+^]^*^, and the photoexcited catalyst was reduced by DIPEA to give Ru(bpy)_3_^+^, in which DIPEA forms **I**. Treatment of **1** with Ru(bpy)_3_^+^ produces **II** regenerating catalyst Ru(bpy)_3_^2+^, subsequent elimination of phthalimide anion (**III**) from **II** provides carboxyl radical **IV**, and release of carbon dioxide in **IV** yields α-amino radical **V**. Addition of **V** to substituted 2-isocyanobiphenyl (**2**) affords imidoyl radical **VI**, intramolecular hemolytic aromatic substitution of **VI** gives radical intermediate **VII**, and oxidation of **VII** with **I** affords cation **VIII** regenerating DIPEA. Finally, deprotonation of **VIII** in the presence of base leads to the target product (**3**).

**Figure 5:**
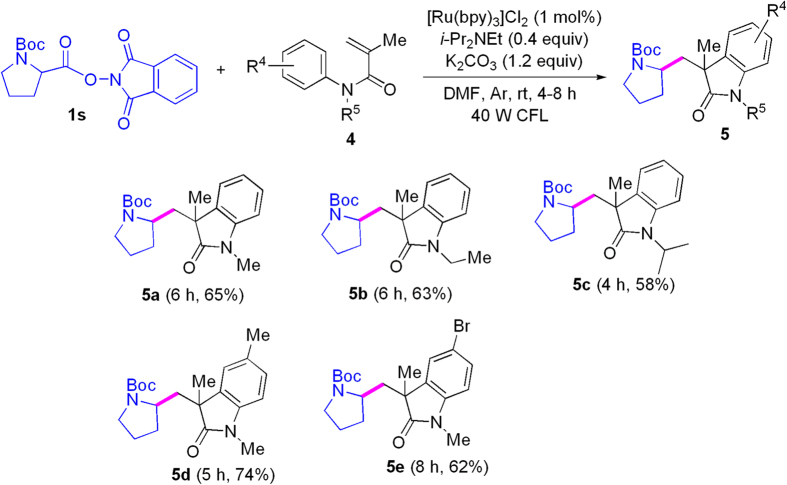
Installing *N*-Boc proline residue on oxindoles under visible-light assistance.

We next explored synthesis of oxindole derivatives under visible-light photoredox catalysis by using *N*-protected amino acid active esters. As shown in Fig. 5, decarboxylative couplings of *N*-Boc-Pro-OPht with *N*-alkyl-*N*-phenylalkacrylamides under the optimized reaction conditions provided the corresponding oxindoles in moderate yields. It is known that oxindoles widely occur in natural products with unique biological activity, and they are the privileged scaffolds for design and discovery of drugs^48^,^49^,^50^. Therefore, the present method affords a novel protocol for synthesis of oxindole derivatives.

In conclusion, we have developed an efficient mode of installing *N*-protected α-amino and peptide residues on *N*-heterocycles through the formation of C-C bonds with the assistant of the photocatalyst [Ru(bpy)_3_]Cl_2_ and visible-light, in which active esters of eighteen *N*-protected amino acids and three peptides were used. The photoredox-generated α-amino or peptide radicals were trapped with substituted 2-isocyanobiphenyls or *N*-alkyl-*N*-phenylalkacrylamides at room temperature, and the phenanthridine and oxindole derivatives with biological and pharmaceutical activity were prepared in good yields. The generation of reactive radicals under the mild photocatalytic conditions may reduce the incidence of undesired side reactions from *N*-protected amino acid and peptide derivatives. Most importantly, the obtained products contain amino acid and peptide fragments, and their further modification can provide diverse molecules after the protective groups on the amino acid and peptide fragments. The present findings pave the way for future synthesis of biological and pharmaceutical molecules containing amino acid and peptide fragments, and we believe that the present strategy will find wide applications in organic synthesis.

## Methods

### General procedure for synthesis of compounds 3a–af and 5a–e

[Ru(bpy)_3_]Cl_2_ (1.5 μmol, 1.2 mg), **1** (0.45 mmol for synthesis of 3**a–p**; 0.30 mmol for synthesis of the others), **2** (0.15 mmol) or **4** (0.15 mmol), DIPEA (0.06 mmol, 10 μL) and K_2_CO_3_ (0.18 mmol, 25 mg) were added to a 25-mL Schlenk tube with DMF (2.0 mL) or mixed solvent of CH_2_Cl_2 _ and DMF (2.0 mL, CH_2_Cl_2_/DMF (5:1)), and the tube was degassed by argon sparging for over 5 min. The tube was sealed, and then irradiated with a 40 W fluorescent lamp (approximately 2 cm away from the light source). After the complete conversion of the substrates (monitored by TLC), the reaction mixture was diluted with 20 L of EtOAc, and the solution was filtered by flash chromatography. The filtrate was evaporated by rotary evaporator, and the residue was purified by silica gel column chromatography to give the desired product (**3a–af** and **5a–e**).

## Additional Information

**How to cite this article**: Jin, Y. *et al*. Installing amino acids and peptides on *N*-heterocycles under visible-light assistance. *Sci. Rep*. **6**, 20068; doi: 10.1038/srep20068 (2016).

## Supplementary Material

Supplementary Information

## Figures and Tables

**Figure 1:**
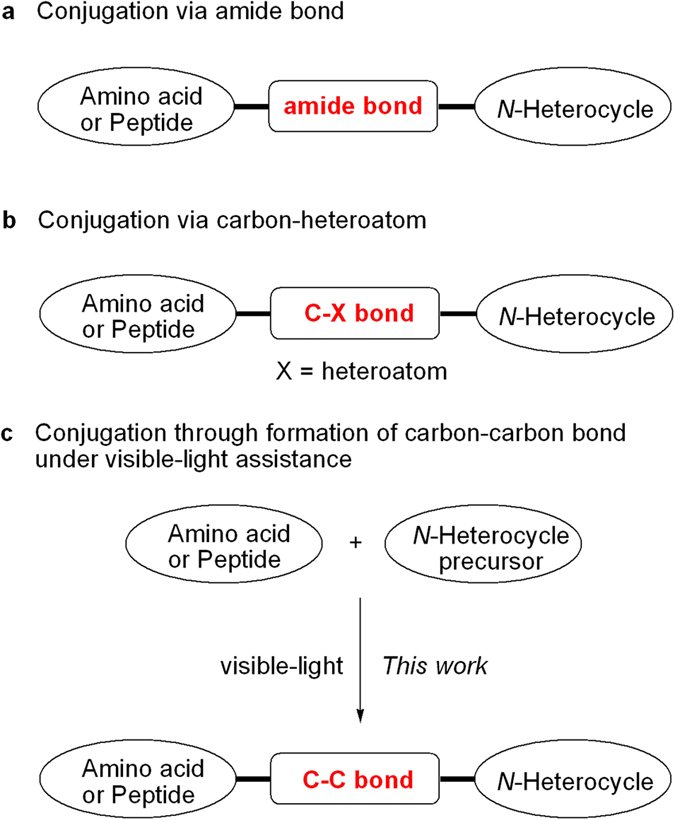
Design on installing amino acids and peptides on *N*-heterocycles. (**a**) The previous conjugation via amide bond. (**b**) The previous conjugation via carbon-heteroatom bond. (**c**) Our conjugation through formation of carbon-carbon bond under visible-light assistance.

**Table 1:**
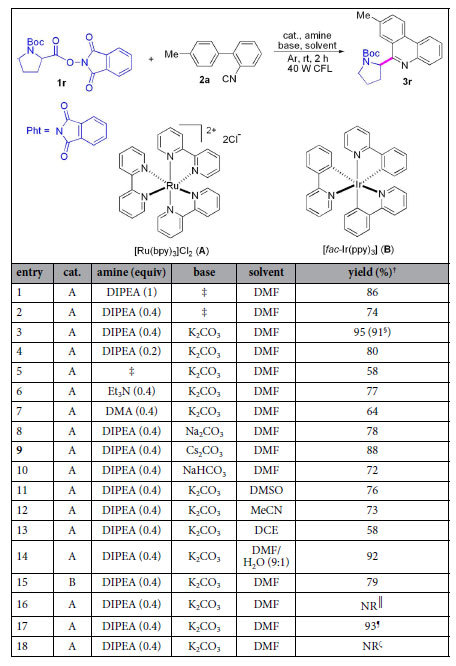
Development of a method for installing *N*-Boc proline residue on 8-methylphenanthridine*.

^*^Reaction conditions: under Ar atmosphere and irradiation of visible light, *N*-Boc-Pro-OPht (**1r**) (0.30 mmol), 1-isocyano-(*p*-phenyl)-benzene (**2a**) (0.15 mmol), catalyst (1.5 μmol), amine (0.03–0.15 mmol), base (0.18 mmol), solvent (2.0 mL), temperature (rt, ~25 ^o^C), time (2 h) in a sealed Schlenk tube. ^†^Conversion yield by ^1^H NMR determination using trichloroethylene as the internal standard. ^‡^No addition of reagent. ^§^Isolated yield. ^║^Under air. ^¶^Under irradiation of blue LED. ^ζ^No light. DIPEA = diisopropylethylamine. DCE = 1,2-dichloroethane. DMA = *N, N*- Dimethylaniline. CFL = compact fluorescent light.
