# Supplementary material for: Installing amino acids and peptides on N-heterocycles under visible-light assistance
Source: Sci Rep. 2016 Feb 2;6:20068. doi: 10.1038/srep20068 (PMC4735789; doi:10.1038/srep20068)
Supplement: Supplementary Information [file srep20068-s1.pdf]

# Supporting Information

## Installing amino acids and peptides on *N*-heterocycles under visible-light assistance

Yunhe Jin, Min Jiang, Hui Wang and Hua Fu*

Key Laboratory of Bioorganic Phosphorus Chemistry and Chemical Biology (Ministry of Education), Department of Chemistry, Tsinghua University, Beijing 100084, P. R. China. Fax: (+86) 10-62781695.

*E-mail: fuhua@mail.tsinghua.edu.cn

**Table of contents**

General experimental procedures P2

General procedure for synthesis of *N*-protected amino acid and peptide active esters (**1**) P2

General procedure for synthesis of substituted 2-isocyanobiphenyls (**2**) P2

General procedure for synthesis of *N*-alkyl-*N*-phenylalkacrylamides (**4**) P3

General procedure for synthesis of compounds **3a-af** and **5a-e** P3

Characterization data of compounds **3a-af** and **5a-e** P4

References P21

The $^{1}$H and $^{13}$C NMR spectra of compounds **3a-af** and **5a-e** P22

**General experimental procedures**

Reactions were carried out under $N_2$ or Ar atmosphere. Proton magnetic resonance spectra ($^1$H NMR) were recorded in the solvent of $CDCl_3$ using tetramethylsilane (TMS) as the internal standard ($^1$H NMR: TMS at 0.00 ppm) and referencing to the residual proton resonance of $CDCl_3$ (7.26 ppm), and carbon magnetic resonance spectra ($^{13}$C NMR) were recorded in the solvent of $CDCl_3$ referencing to the carbon resonance of $CDCl_3$ (77.2 ppm).

**General procedure for synthesis of *N*-protected amino acid and peptide active esters (1)**[1]

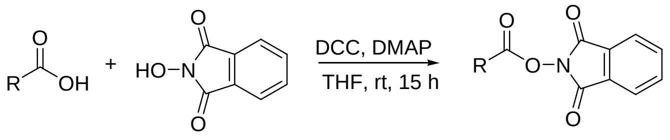


**1**

DCC (1.2 mmol) was added to a solution of *N*-protected amino acid or peptide (1 mmol), *N*-hydroxyphthalimide (1.1 mmol) and DMAP (0.1 mmol) in THF (5 mL). Reaction mixture was stirred at room temperature for 15 h, and the resulting mixture was filtered. The filtrate was evaporated by rotary evaporator, and the residue was purified by column chromatography on flash silica gel ($CH_2Cl_2$ or $CH_2Cl_2$/EtOAc) to provide *N*-protected amino acid or peptide active ester (**1**).

**General procedure for synthesis of substituted 2-isocyanobiphenyls (2)**[2]

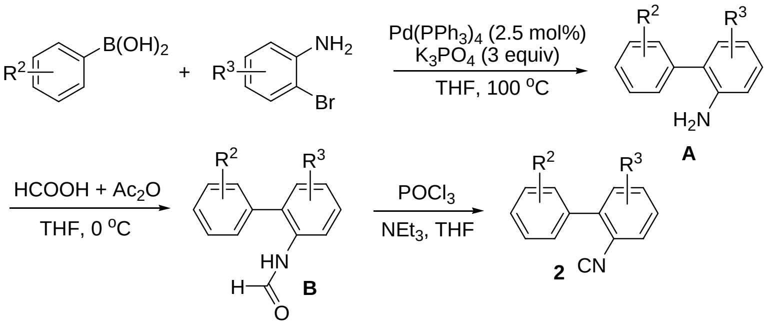


$Pd(PPh_3)_4$ (0.025 mmol, 29 mg), aryl boronic acid (1.5 mmol), substituted 2-bromoaniline (1.0 mmol), $K_3PO_4$ (3.0 mmol, 636 mg) and THF (5.0 mL) were added to a 25 mL two-neck flask under $N_2$ atmosphere. The reaction mixture was stirred at 100 °C for 12 h. After completion of the reaction, the resulting mixture was cooled to room temperature and filtered through a short path of silica gel, eluting with PE : EA (5: 1). The volatile compounds were removed in vacuo, and the residue was

dissolved in 4.0 mL of THF. After being cooled to 0 °C, acetic formic anhydride (10 mmol, 0.9 mL), which was prepared from the reaction of acetic anhydride with formic acid at 55 °C for 2 h, was added dropwise to the solution at 0 °C. After the addition was completed, the mixture was warmed to room temperature and stirred for 1 h. The mixture was treated with saturated aqueous solution of $NaHCO_3$ and extracted with EtOAc three times (3×5 mL). The combined organic phase was dried over anhydrous $Na_2SO_4$ and concentrated under vacuum. The residue was dissolved in 3.0 mL of THF, and $NEt_3$ (6 mmol, 830 μL) was added. The solution was added to an over-dried two-neck flask under $N_2$ atmosphere. After the reaction mixture was cooled to 0 °C with ice bath, $POCl_3$ (8.7 mmol, 0.8 mL) was added *via* syring pump for a period of 2 h. The resulting mixture was stirred at 0 °C for an additional 1 h, and then the reaction was quenched with saturated aqueous solution of $NaHCO_3$. The resulting solution was extracted with EtOAc (3×10 mL). The combined organic layer was dried over anhydrous $Na_2SO_4$, filtered and evaporated, and the residue was purified by a silica gel column chromatography to give substituted 2-isocyanobiphenyls (**2**).

**General procedure for synthesis of *N*-alkyl-*N*-phenylalkacrylamides (4)**

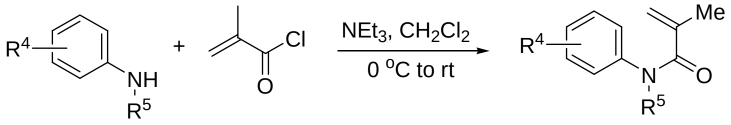

**4**

*N*-Alkyl phenylamine (1.0 mmol) and $NEt_3$ (1.5 mmol) was dissolved in anhydrous $CH_2Cl_2$ (10 mL), and the solution was cooled to 0 °C. Methacryloyl chloride (1.1 mmol) in $CH_2Cl_2$ (1.0 mL) was dropped to the solution, and the mixture was allowed to warm to room temperature and stirred overnight. The resulting solution was sequentially washed with 1N HCl (two times), saturated $NaHCO_3$ solution (two times) and brine. The organic layer was dried over $Na_2SO_4$, filtered and evaporated under vacuum. The residue was purified by a silica gel column chromatography to give *N*-alkyl-*N*-phenylalkacrylamides (**4**).

**General procedure for synthesis of compounds 3a-af and 5a-e**

$[Ru(bpy)_3]Cl_2$ (1.5 μmol, 1.2 mg), **1** (0.45 mmol for synthesis of 3**a-p**; 0.30 mmol for synthesis of the others), **2** (0.15 mmol) or **4** (0.15 mmol), DIPEA (0.6 mmol, 10 μL)

and $K_2CO_3$ (0.18 mmol, 25 mg) were added to a 25-mL Schlenk tube with DMF (2.0 mL) or mixed solvent of $CH_2Cl_2$ and DMF (2.0 mL, $CH_2Cl_2$/DMF (5:1)), and the tube was degassed by argon sparging for over 5 min. The tube was sealed, and then irradiated with a 40 W fluorescent lamp (approximately 2 cm away from the light source). After the complete conversion of the substrates (monitored by TLC), the reaction mixture was diluted with 20 mL of EtOAc, and the solution was filtered by flash chromatography. The filtrate was evaporated by rotary evaporator, and the residue was purified by silica gel column chromatography to give the desired product (**3a-af** and **5a-e**).

**Characterization data of compounds 3a-ae and 5a-e**

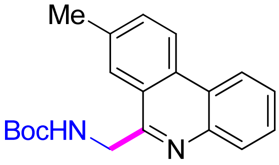


**3a**

***tert*-Butyl ((8-methylphenanthridin-6-yl)methyl)carbamate (3a).** Eluent: PE/EtOAc (15:1). Yield 30.9 mg (64%). White solid, mp 134-136 °C. $^1$H NMR ($CDCl_3$, 400 MHz) δ 8.46 (d, *J* = 8.2 Hz, 2H), 8.11 (d, *J* = 8.2 Hz, 1H), 7.88 (s, 1H), 7.70-7.60 (m, 3H), 6.62 (s, 1H), 4.97 (s, 1H), 2.56 (s, 2H), 1.56 (s, 9H). $^{13}$C NMR ($CDCl_3$, 100 MHz) δ 156.2, 154.4, 142.6, 137.8, 132.6, 130.6, 129.6, 128.3, 126.9, 124.2, 124.1, 122.4, 121.9, 79.5, 43.7, 28.6, 21.8. HRMS (ESI$^+$): Calcd for $C_{20}H_{22}N_2O_2$, $[M+H]^+$ m/z 323.1754. Found 323.1752.

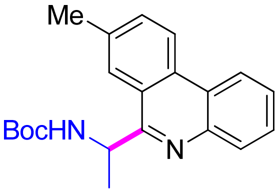


**3b**

***tert*-Butyl-(1-(8-methylphenanthridin-6-yl)ethyl)carbamate (3b).** Eluent: PE/EtOAc (15:1). Yield 43.0 mg (85%). White solid, mp 162-164 °C. $^1$H NMR ($CDCl_3$, 400 MHz) δ 8.48 (t, *J* = 7.8 Hz, 2H), 8.11 (d, *J* = 7.8 Hz, 1H), 8.00 (s, 1H), 7.69-7.58 (m, 3H), 6.75 (d, *J* = 7.3 Hz, 1H), 5.73 (q, *J* = 6.9 Hz, 1H), 2.57 (s, 3H), 1.61 (d, *J* = 6.9 Hz, 3H), 1.52 (s, 9H). $^{13}$C NMR ($CDCl_3$, 100 MHz) δ 160.1, 155.6, 142.7, 137.6, 132.4, 131.1, 129.7, 128.3, 126.8, 124.9, 124.0, 123.5, 122.6, 121.9,

79.3, 47.6, 28.6, 23.2, 21.9. HRMS (ESI+): Calcd for $C_{21}H_{24}N_2O_2$, $[M+H]^+$ m/z 337.1911. Found 337.1916.

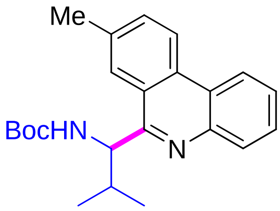

**3c**

***tert*-Butyl-(2-methyl-1-(8-methylphenanthridin-6-yl)propyl)carbamate (3c).** Eluent: PE/EtOAc (15:1). Yield 44.4 mg (81%). White solid, mp 177-179 °C. $^1H$ NMR ($CDCl_3$, 400 MHz) δ 8.47 (dd, $J_1$ = 6.0 Hz, $J_2$ = 7.3 Hz, 2H), 8.11 (d, $J$ = 8.2 Hz, 1H), 8.06 (s, 1H), 7.68-7.57 (m, 3H), 6.38 (d, $J$ = 8.7 Hz, 1H), 5.63 (dd, $J_1$ = 8.7 Hz, $J_2$ = 5.0 Hz, 1H), 2.58 (s, 3H), 2.32-2.25 (m, 1H), 1.48 (s, 9H), 1.08 (d, $J$ = 6.8 Hz, 3H), 0.87 (d, $J$ = 6.4 Hz, 3H). $^{13}C$ NMR ($CDCl_3$, 100 MHz) δ 159.7, 156.4, 142.7, 137.4, 132.3, 130.9, 129.9, 128.2, 126.7, 125.3, 124.4, 123.9, 122.5, 121.8, 79.1, 55.5, 34.6, 28.6, 22.0, 20.7, 17.2. HRMS (ESI+): Calcd for $C_{23}H_{28}N_2O_2$, $[M+H]^+$ m/z 365.2224. Found 365.2220.

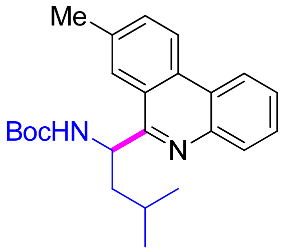

**3d**

***tert*-Butyl-(3-methyl-1-(8-methylphenanthridin-6-yl)butyl)carbamate (3d).** Eluent: PE/EtOAc (15:1). Yield 48.9 mg (86%). White solid, mp 175-177 °C. $^1H$ NMR ($CDCl_3$, 400 MHz) δ 8.48 (t, $J$ = 7.8 Hz, 2H), 8.11-8.06 (m, 2H), 7.68-7.57 (m, 3H), 6.34 (d, $J$ = 8.7 Hz, 1H), 5.81 (dd, $J_1$ = 7.8 Hz, $J_2$ = 6.0 Hz, 1H), 2.58 (s, 3H), 1.95-1.88 (m, 1H), 1.73-1.70 (m, 1H), 1.48 (s, 9H), 1.20 (d, $J$ = 6.4 Hz, 3H), 0.91 (d, $J$ = 6.9 Hz, 3H). $^{13}C$ NMR ($CDCl_3$, 100 MHz) δ 160.6, 156.1. 142.9. 137.5. 132.3. 131.0. 129.8. 128.2. 126.7. 124.9. 124.0. 123.8. 122.5. 121.9. 79.2. 49.7. 47.1. 28.6. 25.4. 23.8. 22.3. 22.0. HRMS (ESI+): Calcd for $C_{24}H_{30}N_2O_2$, $[M+H]^+$ m/z 379.2380. Found 379.2375.

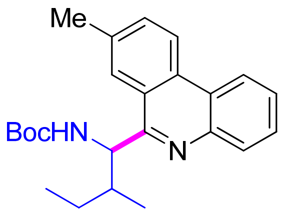


**3e**

***tert*-Butyl-(2-methyl-1-(8-methylphenanthridin-6-yl)butyl)carbamate (3e).** Eluent: PE/EtOAc (15:1). Yield 50.1 mg (88%). White solid, mp 145-147 °C. $^{1}$H NMR ($CDCl_3$, 400 MHz) δ 8.47 (m, 2H), 8.12-8.04 (m, 2H), 7.68-7.57 (m, 3H), 6.43 and 6.31 (d, $J$ = 8.7 Hz, 1H), 5.79 and 5.65 (dd, $J_1$ = 8.7 Hz, $J_2$ = 5.5 Hz, 1H), 2.58 and 2.57 (s, 3H), 2.02 (m, 1H), 1.69 and 1.36 (m, 1H), 1.49 and 1.47 (s, 9H), 1.25-1.17 (m, 1H), 1.12-0.98 (m, 3H), 0.82-0.76 (m, 3H). $^{13}$C NMR ($CDCl_3$, 100 MHz) δ 160.0, 159.6, 156.5, 156.3, 142.7, 142.6, 137.7, 137.4, 132.3, 132.2, 131.0, 130.9, 129.9, 128.2, 126.7, 126.6, 125.3, 125.2, 124.6, 124.2, 123.9, 122.5, 122.4, 121.9, 79.1, 55.2, 53.9, 41.4, 41.2, 28.6, 27.8, 23.8, 22.0, 16.9, 13.8, 12.4, 11.8. HRMS (ESI$^{+}$): Calcd for $C_{24}H_{30}N_2O_2$, $[M+H]^{+}$ m/z 379.2380. Found 379.2378.

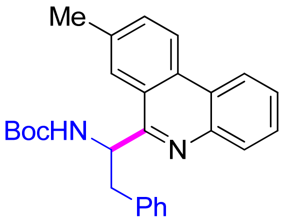


**3f**

***tert*-Butyl-(1-(8-methylphenanthridin-6-yl)-2-phenylethyl)carbamate (3f).** Eluent: PE/EtOAc (15:1). Yield 48.3 mg (78%). White solid, mp 116-118 °C. $^{1}$H NMR ($CDCl_3$, 400 MHz) δ 8.45 (t, $J$ = 7.3 Hz, 2H), 8.06 (d, $J$ = 7.8 Hz, 1H), 7.80 (s, 1H), 7.65-7.56 (m, 3H), 7.07 (s, 3H), 7.00 (s, 2H), 6.40 (d, $J$ = 7.8 Hz, 1H), 5.93 (q, $J$ = 6.9 Hz, 1H), 3.38 (dd, $J_1$ = 6.9 Hz, $J_2$ = 13.3 Hz, 1H), 3.26 (dd, $J_1$ = 5.5 Hz, $J_2$ = 13.3 Hz, 1H), 2.46 (s, 3H), 1.46 (s, 9H). $^{13}$C NMR ($CDCl_3$, 100 MHz) δ 158.9, 155.5, 142.8, 137.6, 137.3, 132.3, 130.8, 129.9, 129.8, 128.2, 128.0, 126.9, 126.4, 125.2, 124.2, 124.0, 122.3, 121.9, 79.4, 52.5, 43.1, 28.6, 21.8. HRMS (ESI$^{+}$): Calcd for $C_{27}H_{28}N_2O_2$, $[M+H]^{+}$ m/z 413.2224. Found 413.2219.

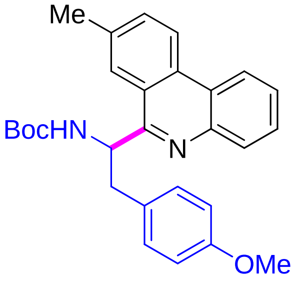

**3g**

***tert*-Butyl-(2-(4-methoxyphenyl)-1-(8-methylphenanthridin-6-yl)ethyl)carbamate (3g).** Eluent: PE/EtOAc (5:1). Yield 55.2 mg (83%). White solid, mp 138-140 °C. $^1$H NMR ($CDCl_3$, 400 MHz) δ 8.49 (dd, $J_1$ = 5.0 Hz, $J_2$ = 8.2 Hz, 2H), 8.07 (d, $J$ = 7.3 Hz, 1H), 7.82 (s, 1H), 7.69-7.59 (m, 3H), 6.85 (d, $J$ = 8.2 Hz, 2H), 6.62 (d, $J$ = 8.2 Hz, 2H), 6.40 (d, $J$ = 8.6 Hz, 1H), 5.88 (q, $J$ = 6.4 Hz, 1H), 3.70 (s, 3H), 3.28 (dd, $J_1$ = 6.9 Hz, $J_2$ = 13.3 Hz, 1H), 3.21 (dd, $J_1$ = 6.0 Hz, $J_2$ = 13.3 Hz, 1H), 2.48 (s, 3H), 1.46 (s, 9H). $^{13}$C NMR ($CDCl_3$, 100 MHz) δ 159.0, 158.2, 155.5, 142.8, 137.3, 134.3, 132.3, 130.8, 129.8, 129.7, 128.2, 126.8, 125.2, 124.2, 124.0, 122.3, 121.9, 113.4, 79.3, 55.2, 52.6, 42.1, 28.6, 21.8. HRMS (ESI$^+$): Calcd for $C_{28}H_{30}N_2O_3$, $[M+H]^+$ m/z 443.2329. Found 443.2324.

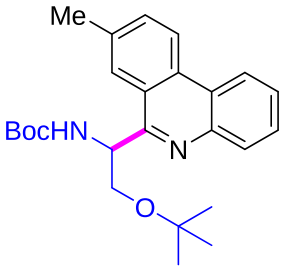

**3h**

***tert*-Butyl-(2-(tert-butoxy)-1-(8-methylphenanthridin-6-yl)ethyl)carbamate (3h).** Eluent: PE/EtOAc (15:1). Yield 19.6 mg (32%). White solid, mp 195-197 °C. $^1$H NMR ($CDCl_3$, 400 MHz) δ 8.51 (d, $J$ = 8.7 Hz, 2H), 8.24 (s, 1H), 8.12 (d, $J$ = 8.7 Hz, 1H), 7.71-7.59 (m, 3H), 6.41 (d, $J$ = 7.3 Hz, 1H), 5.81 (q, $J$ = 6.6 Hz, 1H), 3.84 (dd, $J_1$ = 5.5 Hz, $J_2$ = 9.2 Hz, 1H), 3.68 (dd, $J_1$ = 6.9 Hz, $J_2$ = 9.2 Hz, 1H), 2.60 (s, 3H), 1.49 (s, 9H), 0.98 (s, 9H). $^{13}$C NMR ($CDCl_3$, 100 MHz) δ 158.7, 155.7, 142.9, 137.0, 132.3, 130.7, 129.8, 128.1, 126.8, 126.5, 125.0, 124.1, 122.0, 121.9, 79.3, 73.2, 66.1, 51.8, 28.6, 27.3, 21.8. HRMS (ESI$^+$): Calcd for $C_{25}H_{32}N_2O_3$, $[M+H]^+$ m/z 409.2486. Found 409.2483.

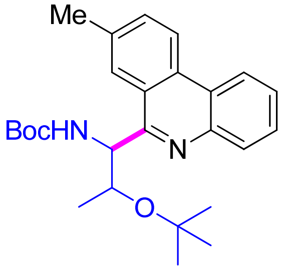


**3i**

***tert*-Butyl-(2-(tert-butoxy)-1-(8-methylphenanthridin-6-yl)propyl)carbamate (3i).** Eluent: PE/EtOAc (15:1). Yield 56.5 mg (89%). White solid, mp 163-165 °C. $^{1}H$ NMR ($CDCl_3$, 400 MHz) δ 8.55-8.47 (m, 2H), 8.28-8.22 (m, 1H), 8.17-8.06 (m, 1H) 7.71-7.58 (m, 3H), 6.54 and 6.36 (d, *J* = 7.3 Hz, 1H), 5.73-5.61 (m, 1H), 4.24-3.83 (m, 1H), 2.60 (s, 1H), 1.49 and 1.46 (s, 9H), 1.35 (brd, *J* = 6.9 Hz, 2H), 1.14 (brd, *J* = 5.5 Hz 1H), 1.05 (brs, 2.7H), 0.62 (brs, 6.3H). $^{13}C$ NMR ($CDCl_3$, 100 MHz) δ 160.8, 157.8, 156.0, 155.9, 142.9, 142.7, 136.8, 132.2, 131.0, 130.3, 130.0, 129.7, 128.1, 128.0, 127.3, 126.8, 126.7, 126.5, 126.0, 125.2, 124.0, 122.2, 121.9, 121.8, 121.7, 79.2, 77.3, 74.2, 73.7, 72.7, 70.4, 55.7, 55.5, 28.6, 28.5, 28.2, 28.0, 22.0, 21.8, 20.5, 19.4. HRMS (ESI$^{+}$): Calcd for $C_{26}H_{34}N_2O_3$, $[M+H]^{+}$ m/z 423.2642. Found 423.2639.

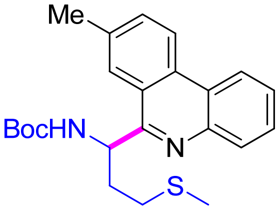


**3j**

***tert*-Butyl-(1-(8-methylphenanthridin-6-yl)-3-(methylthio)propyl)carbamate (3j).** Eluent: PE/EtOAc (15:1). Yield 55.4 mg (93%). White solid, mp 161-163 °C. $^{1}H$ NMR ($CDCl_3$, 400 MHz) δ 8.48 (t, *J* = 7.8 Hz, 2H), 8.11 (s, 1H), 8.09 (d, *J* = 7.8 Hz, 1H), 7.69-7.58 (m, 3H), 6.55 (d, *J* = 7.8 Hz, 1H), 5.92-5.85 (m, 1H), 2.78-2.69 (m, 1H), 2.58 (s, 3H), 2.55-2.46 (m, 1H), 2.38-2.28 (m, 1H), 2.12-2.00 (m, 4H), 1.50 (s, 9H). $^{13}C$ NMR ($CDCl_3$, 100 MHz) δ 158.9, 156.0, 142.6, 137.7, 132.5, 131.0, 129.8, 128.3, 126.9, 124.9, 124.0, 123.7, 122.6, 121.9, 79.5, 50.4, 36.7, 30.6, 28.6, 21.9, 15.6. HRMS (ESI$^{+}$): Calcd for $C_{23}H_{28}N_2O_2S$, $[M+H]^{+}$ m/z 397.1944. Found 397.1939.

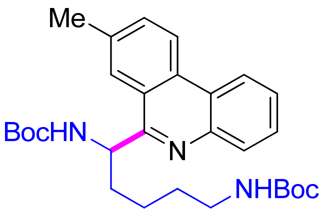


**3k**

**Di-*tert*-butyl-(1-(8-methylphenanthridin-6-yl)pentane-1,5-diyl)dicarbamate (3k).** Eluent: PE/EtOAc (5:1). Yield 60.1 mg (81%). White solid, mp 146-148 °C. $^1$H NMR ($CDCl_3$, 400 MHz) δ 8.48 (t, $J$ = 8.7 Hz, 2H), 8.09 (d, $J$ = 7.8 Hz, 1H), 7.99 (s, 1H), 7.68-7.57 (m, 3H), 6.54 (d, $J$ = 7.8 Hz, 1H), 5.76-5.66 (m, 1H), 4.70 (s, 1H), 3.16-2.98 (m, 2H), 2.58 (s, 3H), 2.10-1.97 (m, 1H), 1.84-1.72 (m, 1H), 1.68-1.32 (m, 4H), 1.49 (s, 9H) , 1.40 (s, 9H). $^{13}$C NMR ($CDCl_3$, 100 MHz) δ 159.5, 156.1, 142.6, 137.6, 132.5, 131.0, 129.8, 128.2, 126.8, 124.8, 124.0, 123.7, 122.6, 121.9, 79.4, 78.9, 50.7, 40.6, 36.9, 29.5, 28.6, 28.5, 22.8, 22.0. HRMS (ESI$^+$): Calcd for $C_{29}H_{39}N_3O_4$, $[M+H]^+$ m/z 494.3013. Found 494.3010.

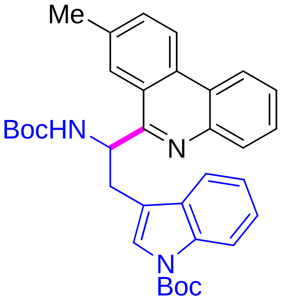


**3l**

***tert*-Butyl-3-(2-((tert-butoxycarbonyl)amino)-2-(8-methylphenanthridin-6-yl)ethyl)-1H-indole-1-carboxylate (3l).** Eluent: PE/EtOAc (15:1). Yield 50.5 mg (61%). Clear oil. $^1$H NMR ($CDCl_3$, 400 MHz) δ 8.51-8.42 (m, 2H), 8.09-8.01 (m, 2H), 7.74 (s, 1H), 7.69-7.58 (m, 2H), 7.55 (d, $J$ = 9.2 Hz, 1H), 7.41 (d, $J$ = 7.3 Hz, 1H), 7.21 (t, $J$ = 8.2 Hz, 1H), 7.08 (t, $J$ = 7.3 Hz, 1H), 6.90 (s, 1H), 6.53 (d, $J$ = 7.3 Hz, 1H), 6.02 (q, $J$ = 7.8 Hz, 1H), 3.40 (d, $J$ = 6.9 Hz, 2H), 2.32 (s, 3H), 1.50 (s, 9H), 1.49 (s, 9H). $^{13}$C NMR ($CDCl_3$, 100 MHz) δ 158.9, 155.6, 149.5, 142.7, 137.2, 135.4, 132.3, 131.2, 130.7, 129.8, 128.2, 126.9, 125.0, 124.3, 124.2, 124.1, 124.0, 122.5, 122.2, 121.8, 119.1, 116.4, 115.0, 83.0, 79.4, 51.1, 32.4, 28.6, 28.1, 21.6. HRMS (ESI$^+$): Calcd for $C_{34}H_{37}N_3O_4$, $[M+H]^+$ m/z 552.2857. Found 552.2856.

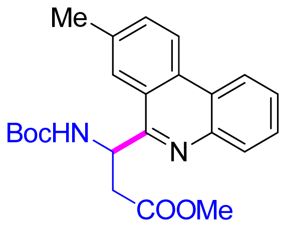


**3m**

**Methyl-3-((*tert*-butoxycarbonyl)amino)-3-(8-methylphenanthridin-6-yl)propanoate (3m).** Eluent: PE/EtOAc (5:1). Yield 48.6 mg (82%). White solid, mp 162-164 °C. $^{1}$H NMR ($CDCl_3$, 400 MHz) δ 8.48 (t, $J$ = 8.2 Hz, 2H), 8.18 (s, 1H), 8.06 (d, $J$ = 7.8 Hz, 1H), 7.68-7.59 (m, 3H), 6.22 (d, $J$ = 8.7 Hz, 1H), 6.15-6.07 (m, 1H), 3.66 (s, 3H), 3.11 (dd, $J_1$ = 5.5 Hz, $J_2$ = 14.6 Hz, 1H), 2.91 (dd, $J_1$ = 6.9 Hz, $J_2$ = 14.6 Hz, 1H), 2.59 (s, 3H), 1.48 (s, 9H). $^{13}$C NMR ($CDCl_3$, 100 MHz) δ 171.9, 157.8, 155.3, 142.6, 137.8, 132.6, 131.2, 129.9, 128.3, 127.1, 125.0, 124.2, 123.9, 122.5, 121.9, 79.8, 51.9, 49.0, 40.7, 28.5, 22.0. HRMS (ESI$^+$): Calcd for $C_{23}H_{26}N_2O_4$, $[M+H]^+$ m/z 395.1965. Found 395.1966.

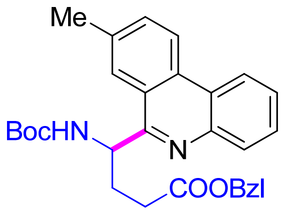


**3n**

**Benzyl-4-((*tert*-butoxycarbonyl)amino)-4-(8-methylphenanthridin-6-yl)butanoate (3n).** Eluent: PE/EtOAc (5:1). Yield 55.3 mg (76%). Clear oil. $^{1}$H NMR ($CDCl_3$, 400 MHz) δ 8.48 (t, $J$ = 7.3 Hz, 2H), 8.17 (s, 1H), 8.09 (d, $J$ = 7.8 Hz, 1H), 7.68-7.59 (m, 3H), 7.33-7.25 (m, 5H), 6.61 (d, $J$ = 7.3 Hz, 1H), 5.88-5.81 (m, 1H), 5.08 (d, $J$ = 12.4 Hz, 1H), 5.00 (d, $J$ = 12.4 Hz, 1H), 2.69-2.60 (m, 1H), 2.59 (s, 3H), 2.55-2.37 (m, 1H), 2.10-2.01 (m, 1H), 1.49 (s, 9H). $^{13}$C NMR ($CDCl_3$, 100 MHz) δ 173.3, 158.8, 156.0, 142.6, 137.8, 136.1, 132.6, 131.0, 129.8, 128.7, 128.6, 128.3, 128.2, 127.0, 125.1, 124.1, 123.8, 122.5, 121.9, 79.5, 66.3, 50.3, 31.7, 30.1, 28.6, 22.0. HRMS (ESI$^+$): Calcd for $C_{30}H_{32}N_2O_4$, $[M+H]^+$ m/z 485.2435. Found 485.2434.

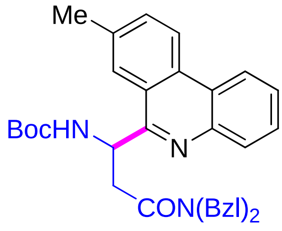


**3o**

***tert*-Butyl-(3-(dibenzylamino)-1-(8-methylphenanthridin-6-yl)-3-oxopropyl)carbamate (3o).** Eluent: PE/EtOAc (5:1). Yield 56.3 mg (67%). White solid, mp 181-183 °C. $^{1}$H NMR ($CDCl_3$, 400 MHz) δ 8.53 (d, *J* = 7.9 Hz, 2H), , 8.40 (s, 1H), 8.01 (d, *J* = 7.6 Hz, 1H), 7.68-7.61 (m, 3H), 7.37-6.98 (m, 10H), 6.39-6.33 (m, 1H), 5.80 (m, 1H), 4.63-4.35 (m, 4H), 3.62-3.55 (m, 1H), 3.16-3.09 (m, 1H), 2.61 (s, 3H), 1.44 (s, 9H). $^{13}$C NMR ($CDCl_3$, 100 MHz) δ 171.6, 159.2, 155.2, 142.7, 137.9, 137.2, 136.8, 132.6, 131.2, 129.8, 129.0, 128.5, 128.2, 128.1, 127.6, 127.2, 127.0, 126.7, 125.9, 124.6, 124.5, 122.3, 122.0, 79.7, 50.2, 49.4, 48.1, 37.9, 28.5, 22.0. HRMS (ESI$^+$): Calcd for $C_{36}H_{37}N_3O_3$, $[M+H]^+$ m/z 560.2908. Found 560.2905.

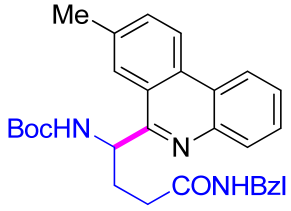


**3p**

***tert*-Butyl-(4-(benzylamino)-1-(8-methylphenanthridin-6-yl)-4-oxobutyl)carbamate (3p).** Eluent: PE/EtOAc (1:1). Yield 54.5 mg (75%). White solid, mp 176-178 °C. $^{1}$H NMR ($CDCl_3$, 400 MHz) δ 8.51 (dd, $J_1$ = 4.1 Hz, $J_2$ = 7.8 Hz, 2H), 8.08 (d, *J* = 7.8 Hz, 1H), 7.94 (s, 1H), 7.70-7.61 (m, 3H), 7.39-7.23 (m, 5H), 7.11(s, 1H), 6.81 (d, *J* = 7.3 Hz, 1H), 5.74 (t, *J* = 7.3 Hz, 1H), 4.56 (dd, $J_1$ = 5.5 Hz, $J_2$ = 15.1 Hz, 1H), 4.50 (dd, $J_1$ = 5.5 Hz, $J_2$ = 15.1 Hz, 1H), 2.59-2.41 (m, 2H), 2.54 (s, 3H), 2.38-2.27 (m, 1H), 2.02-1.88 (m, 1H), 1.50 (s, 9H). $^{13}$C NMR ($CDCl_3$, 100 MHz) δ 172.8, 158.7, 156.8, 142.4, 138.7, 138.0, 132.7, 131.0, 129.7, 128.7, 128.3, 127.9, 127.4, 127.0, 125.0, 124.2, 123.5, 122.5, 122.0, 79.8, 50.6, 43.8, 34.1, 33.2, 28.5, 22.0. HRMS (ESI$^+$): Calcd for $C_{30}H_{33}N_3O_3$, $[M+H]^+$ m/z 484.2595. Found 484.2594.

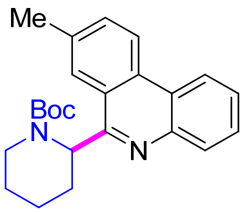

**3q**

***tert*-Butyl-2-(8-methylphenanthridin-6-yl)piperidine-1-carboxylate (3q).** Eluent: PE/EtOAc (15:1). Yield 51.5 mg (91%). White solid, mp 123-125 °C. $^{1}$H NMR ($CDCl_3$, 400 MHz) δ 8.48 (t, *J* = 8.7 Hz, 2H), 8.09 (d, *J* = 8.2 Hz, 2H), 7.66-7.56 (m, 3H), 6.15 (m, 1H), 4.00 (m, 1H), 3.60 (m, 1H), 2.56 (s, 3H), 2.34 (m, 1H), 2.11-1.82 (m, 2H), 1.78-1.50 (m, 3H), 1.36 (s, 9H). $^{13}$C NMR ($CDCl_3$, 100 MHz) δ 160.7, 156.2, 142.8, 137.1, 131.8, 131.0, 130.4, 128.0, 126.6, 125.3, 124.5, 123.8, 122.5, 121.7, 79.4, 52.9, 42.6, 28.6, 28.5, 25.6, 22.0, 19.8. HRMS (ESI$^+$): Calcd for $C_{24}H_{28}N_2O_2$, $[M+H]^+$ m/z 377.2224. Found 377.2224.

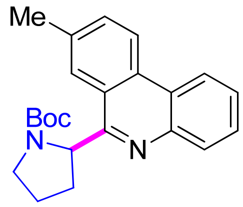

**3r**

***tert*-Butyl-2-(8-methylphenanthridin-6-yl)pyrrolidine-1-carboxylate (3r).** Eluent: PE/EtOAc (5:1). Yield 49.5 mg (91%). White solid, mp 201-203 °C. The product gives two sets of NMR signals, owing to the presence of rotamers around the tertiary amide.[3] $^{1}$H NMR ($CDCl_3$, 400 MHz) δ 8.56-8.49 (m, 2H), 8.09-8.00 (m, 2H), 7.67-7.59 (m, 3H), 5.90-5.70 (m, 1H), 4.01-3.90 (m, 1H), 3.79-3.58 (m, 1H), 2.61-2.42 (m, 4H), 2.08-1.95 (m, 3H), 1.47 (brs, 3.5H), 0.94 (brs, 5.5H). $^{13}$C NMR ($CDCl_3$, 100 MHz) δ 160.9, 160.0, 154.9, 154.8, 143.3, 143.2, 137.0, 131.9, 131.8, 131.2, 130.9, 130.3, 128.2, 127.8, 126.4, 126.3, 124.8, 124.6, 124.3, 124.1, 123.7, 122.6, 121.7, 79.0, 78.8, 59.9, 59.3, 47.2, 47.0, 33.4, 32.2, 28.7, 28.1, 23.8, 23.5, 22.0. HRMS (ESI$^+$): Calcd for $C_{23}H_{26}N_2O_2$, $[M+H]^+$ m/z 363.2067. Found 363.2063.

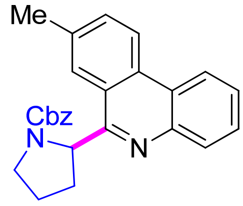

**3s**

**Benzyl-2-(8-methylphenanthridin-6-yl)pyrrolidine-1-carboxylate (3s).** Eluent: PE/EtOAc (5:1). Yield 54.8 mg (92%). White solid, mp 111-113 °C. $^1$H NMR ($CDCl_3$, 400 MHz) δ 8.52-8.42 (m, 2H), 8.06-7.94 (m, 2H), 7.79-7.54 (m, 4H), 7.42-7.28 (m, 2H), 6.79 and 6.65 (d, *J* = 7.3 Hz, 2H), 5.96 and 5.85 (d, *J* = 7.8 Hz, 1H), 5.22 and 5.12 (d, *J* = 12.8 Hz, 1H), 4.98 and 4.82 (d, *J* = 12.8 Hz, 1H), 4.10-3.99 (m, 1H), 3.84-3.69 (m, 1H), 2.60-2.43 (m, 4H), 2.15-1.91 (m, 3H). $^{13}$C NMR ($CDCl_3$, 100 MHz) δ 160.1, 159.7, 156.2, 143.2, 137.1, 136.9, 134.2, 132.0, 131.2, 130.6, 130.5, 128.5, 128.2, 127.9, 127.8, 127.7, 127.1, 126.9, 126.6, 126.5, 124.8, 124.6, 124.2, 124.1, 124.0, 123.9, 123.5, 122.7, 122.6, 121.7, 121.6, 66.6, 66.3, 59.6, 59.5, 47.7, 47.0, 33.4, 32.3, 23.8, 23.2, 22.0. HRMS (ESI$^+$): Calcd for $C_{26}H_{24}N_2O_2$, $[M+H]^+$ m/z 397.1911 Found 397.1909.

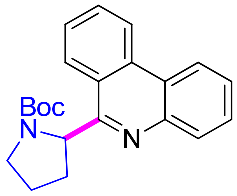


**3t**

***tert*-Butyl-2-(phenanthridin-6-yl)pyrrolidine-1-carboxylate (3t).** Eluent: PE/EtOAc (5:1). Yield 48.2 mg (92%). White solid, mp 146-148 °C. $^1$H NMR ($CDCl_3$, 400 MHz) δ 8.69-8.60 (m, 1H), 8.57-8.48 (m, 1H), 8.29-8.23 (m, 1H), 8.12-8.04 (m, 1H), 7.85-7.76 (m, 1H), 7.72-7.56 (m, 3H), 5.92-5.68 (m, 1H), 4.00-3.87 (m, 1H), 3.76-3.59 (m, 1H), 2.58-2.44 (m, 1H), 2.15-1.86 (m, 3H), 1.47 (brs, 2.9H), 0.93 (brs, 6.1H). $^{13}$C NMR ($CDCl_3$, 100 MHz) δ 161.3, 160.2, 154.9, 154.8, 143.6, 143.5, 133.4, 133.2, 130.4, 130.2, 130.1, 128.6, 128.3, 127.2, 127.1, 126.5, 126.4, 125.3, 125.0, 124.1, 124.0, 123.9, 123.6, 122.8, 122.7, 121.9, 79.1, 78.8, 60.2, 59.4, 47.2, 47.0, 33.4, 32.3, 28.7, 28.1, 23.8, 23.5. HRMS (ESI$^+$): Calcd for $C_{22}H_{24}N_2O_2$, $[M+H]^+$ m/z 349.1911. Found 349.1915.

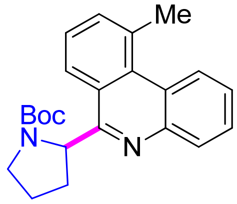


**3u**

***tert*-Butyl-2-(10-methylphenanthridin-6-yl)pyrrolidine-1-carboxylate (3u).** Eluent:

PE/EtOAc (5:1). Yield 27.2 mg (50%). White solid, mp 131-133 °C. $^1$H NMR ($CDCl_3$, 400 MHz) δ 8.81-8.73 (m, 1H), 8.20-8.09 (m, 2H), 7.71-7.53 (m, 4H), 5.92-5.71 (m, 1H), 4.01-3.88 (m, 1H), 3.76-3.59 (m, 1H), 3.14 and 3.10 (s, 3H), 2.60-2.43 (m, 1H), 2.12-1.87 (m, 3H), 1.48 (brs, 3.1H), 0.95 (brs, 5.9H). $^{13}$C NMR ($CDCl_3$, 100 MHz) δ 161.4, 160.4, 155.0, 154.9, 144.8, 144.7, 135.9, 135.8, 134.4, 134.3, 132.7, 130.8, 127.9, 127.6, 126.6, 126.5, 126.4, 125.7, 125.5, 125.4, 125.3, 125.1, 123.7, 123.4, 79.0, 78.7, 60.3, 59.8, 47.2, 47.0, 33.3, 32.3, 28.7, 28.2, 27.1, 27.0, 23.7, 23.4. HRMS (ESI$^+$): Calcd for $C_{23}H_{26}N_2O_2$, $[M+H]^+$ m/z 363.2067. Found 363.2066.

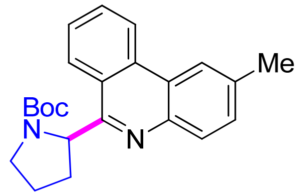


**3v**

***tert*-Butyl-2-(2-methylphenanthridin-6-yl)pyrrolidine-1-carboxylate (3v).** Eluent: PE/EtOAc (5:1). Yield 50.2 mg (92%). White solid, mp 178-180 °C. $^1$H NMR ($CDCl_3$, 400 MHz) δ 8.67-8.59 (m, 1H), 8.33-8.20 (m, 2H), 8.01-7.93 (m, 1H), 7.82-7.74 (m, 1H), 7.67-7.61 (m, 1H), 7.53-7.46 (m, 1H), 5.89-5.66 (m, 1H), 3.98-3.88 (m, 1H), 3.76-3.59 (m, 1H), 2.63-2.43 (m, 4H), 2.14-1.87 (m, 3H), 1.47 (brs, 2.7H), 0.92 (brs, 6.3H). $^{13}$C NMR ($CDCl_3$, 100 MHz) δ 160.2, 159.2, 154.9, 154.8, 142.0, 141.8, 136.3, 136.1, 133.2, 132.9, 130.3, 130.1, 130.0, 129.8, 127.0, 126.9, 125.2, 125.0, 124.0, 123.8, 123.4, 122.7, 122.6, 121.5, 79.0, 78.8, 60.2, 59.4, 47.2, 47.0, 33.4, 32.2, 28.7, 28.1, 23.8, 23.5, 22.0. HRMS (ESI$^+$): Calcd for $C_{23}H_{26}N_2O_2$, $[M+H]^+$ m/z 363.2067. Found 363.2070.

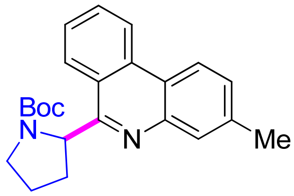


**3w**

***tert*-Butyl-2-(3-methylphenanthridin-6-yl)pyrrolidine-1-carboxylate (3w).** Eluent: PE/EtOAc (5:1). Yield 51.2 mg (94%). White solid, mp 132-134 °C. $^1$H NMR ($CDCl_3$, 400 MHz) δ 8.64-8.53 (m, 1H), 8.43-8.34 (m, 1H), 8.27-8.19 (m, 1H), 7.93-7.57 (m, 3H), 7.46-7.37 (m, 1H), 5.92-5.68 (m, 1H), 4.00-3.87 (m, 1H), 3.77-3.59 (m, 1H), 2.59-2.42 (m, 4H), 2.14-1.87 (m, 3H), 1.48 (brs, 3.1H), 0.94 (brs, 5.9H). $^{13}$C NMR

(CDCl$_3$, 100 MHz) δ 161.2, 160.3, 154.9, 154.8, 143.8, 143.6, 138.8, 138.4, 134.2, 134.0, 133.4, 133.2, 130.1, 130.0, 129.9, 128.3, 128.1, 126.7, 126.6, 125.3, 125.0, 123.8, 123.6, 122.5, 122.4, 121.7, 121.3, 79.0, 78.8, 60.1, 59.4, 47.2, 47.0, 33.4, 32.3, 28.4, 28.1, 23.8, 23.5, 21.6. HRMS (ESI$^+$): Calcd for $C_{23}H_{26}N_2O_2$, $[M+H]^+$ m/z 363.2067. Found 363.2067.

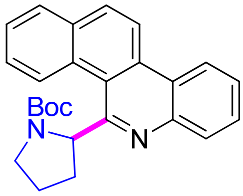


**3x**

***tert*-Butyl-2-(benzo[i]phenanthridin-5-yl)pyrrolidine-1-carboxylate (3x).** PE/EtOAc (5:1). Yield 52.7 mg (88%). White solid, mp 150-152 °C. $^1$H NMR (CDCl$_3$, 400 MHz) δ 8.62-8.51 (m, 3H), 8.15-7.95 (m, 3H), 7.76-7.57 (m, 4H), 6.31-5.99 (m, 1H), 4.13-3.99 (m, 1H), 3.73-3.60 (m, 1H), 2.88-2.54 (m, 2H), 2.39-2.04 (m, 2H), 1.38 (brs, 2.7H), 0.53 (brs, 6.3H). $^{13}$C NMR (CDCl$_3$, 100 MHz) δ 162.6, 160.8, 154.8, 154.1, 144.3, 143.9, 134.0, 133.5, 133.2, 133.1, 131.8, 131.5, 130.0, 129.6, 129.2, 128.9, 128.8, 128.5, 127.6, 127.5, 127.0, 126.8, 126.5, 126.4, 126.3, 126.2, 123.3, 123.0, 122.6, 122.3, 122.2, 121.8, 120.6, 120.4, 78.8, 78.2, 62.7, 61.8, 47.9, 47.8, 34.1, 33.1, 28.4, 27.6, 23.6, 23.4. HRMS (ESI$^+$): Calcd for $C_{26}H_{26}N_2O_2$, $[M+H]^+$ m/z 399.2067. Found 399.2071.

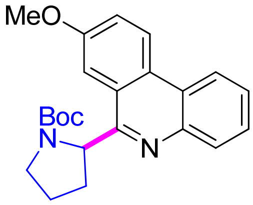


**3y**

***tert*-Butyl-2-(8-methoxyphenanthridin-6-yl)pyrrolidine-1-carboxylate (3y).** PE/EtOAc (4:1). Yield 54.1 mg (95%). White solid, mp 159-161 °C. $^1$H NMR (CDCl$_3$, 400 MHz) δ 8.60-8.51 (m, 1H), 8.47-8.40 (m, 1H), 8.09-8.02 (m, 1H), 7.65-7.52 (m, 3H), 7.48-7.40 (m, 1H), 5.85-5.61 (m, 1H), 4.02-3.87 (m, 4H), 2.58-2.43 (m, 1H), 2.17-1.89 (m, 3H), 1.47 (brs, 3.2H), 0.93 (brs, 5.8H). $^{13}$C NMR (CDCl$_3$, 100 MHz) δ 160.3, 159.3, 158.5, 155.0, 154.7, 142.8, 142.7, 130.4, 130.3, 127.8, 127.7, 127.5, 127.3, 126.6, 126.4, 125.3, 125.1, 124.4, 124.3, 124.1, 123.7, 121.4, 120.5, 120.3,

105.8, 79.1, 78.8, 60.6, 59.6, 55.7, 55.6, 47.1, 47.0, 33.1, 31.9, 28.7, 28.1, 23.8, 23.5. HRMS (ESI$^+$): Calcd for $C_{23}H_{26}N_2O_3$, $[M+H]^+$ m/z 379.2016. Found 379.2016.

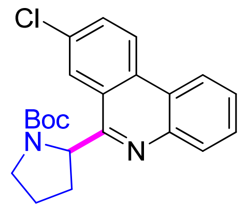


**3z**

***tert*-Butyl-2-(8-chlorophenanthridin-6-yl)pyrrolidine-1-carboxylate (3z).** Eluent: PE/EtOAc (5:1). Yield 54.1 mg (94%). White solid, mp 188-190 °C. $^1$H NMR ($CDCl_3$, 400 MHz) δ 8.62-8.41 (m, 2H), 8.22 (m, 2H), 8.13-8.04 (m, 1H), 7.80-7.55 (m, 3H), 5.82-5.62 (m, 1H), 3.99-3.86 (m, 1H), 3.77-3.60 (m, 1H), 2.61-2.43 (m, 1H), 2.14-1.97 (m, 3H), 1.47 (brs, 3.6H), 0.96 (brs, 5.4H). $^{13}$C NMR ($CDCl_3$, 100 MHz) δ 160.4, 159.4, 154.8, 154.6, 143.6, 143.4, 133.2, 131.8, 131.5, 130.8, 130.7, 130.5, 129.0, 128.7, 127.0, 126.8, 124.9, 124.7, 124.5, 124.4, 123.4, 123.0, 121.8, 79.2, 78.9, 59.6, 59.3, 47.2, 47.0, 33.4, 32.2, 28.7, 28.1, 23.9, 23.5. HRMS (ESI$^+$): Calcd for $C_{22}H_{23}N_2O_2Cl$, $[M+H]^+$ m/z 383.1521. Found 383.1517.

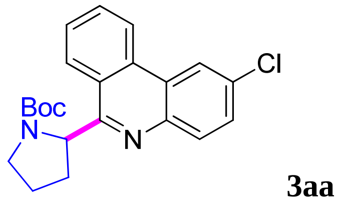


**3aa**

***tert*-Butyl-2-(2-chlorophenanthridin-6-yl)pyrrolidine-1-carboxylate (3aa).** Eluent: PE/EtOAc (5:1). Yield 53.5 mg (93%). White solid, mp 198-200 °C. $^1$H NMR ($CDCl_3$, 400 MHz) δ 8.59-8.45 (m, 2H), 8.29-8.24 (m, 1H), 8.06-7.97 (m, 1H), 7.87-7.78 (m, 1H), 7.74-7.57 (m, 2H), 5.88-5.69 (m, 1H), 3.97-3.87 (m, 1H), 3.76-3.59 (m, 1H), 2.58-2.46 (m, 3H), 1.47 (brs, 3.1H), 0.92 (brs, 5.9H). $^{13}$C NMR ($CDCl_3$, 100 MHz) δ 161.8, 160.7, 154.9, 124.6, 142.1, 142.0, 132.4, 132.2, 132.1, 131.8, 130.5, 130.4, 129.2, 128.8, 127.9, 127.8, 125.4, 125.1, 124.7, 124.3, 124.1, 122.8, 122.7, 121.6, 79.2, 78.8, 60.0, 59.4, 47.2, 47.0, 33.3, 32.2, 28.7, 28.1, 23.8, 23.5. HRMS (ESI$^+$): Calcd for $C_{22}H_{23}N_2O_2Cl$, $[M+H]^+$ m/z 383.1521. Found 383.1523.

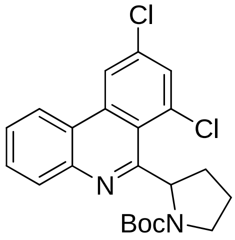


**3ab**

***tert*-Butyl-2-(7,9-dichlorophenanthridin-6-yl)pyrrolidine-1-carboxylate (3ab).** Eluent: PE/EtOAc (8:1). Yield 39.4 mg (63%). White solid, mp 139-141 °C. $^{1}$H NMR ($CDCl_3$, 400 MHz) δ 8.57 and 8.53 (s, 1H), 8.47-8.32 (m, 1H), 8.09-7.96 (m, 1H), 7.82-7.55 (m, 3H), 6.52-6.32 (m, 1H), 4.02-3.85 (m, 1H), 3.70-3.53 (m, 1H), 2.68-2.46 (m, 1H), 2.32-1.83 (m, 3H), 1.46 (brs, 3.4H), 0.93 (brs, 5.6H). $^{13}$C NMR ($CDCl_3$, 100 MHz) δ 160.6, 159.0, 155.0, 154.5, 143.1, 143.0, 137.4, 137.0, 135.7, 135.6, 133.0, 132.8, 130.7, 130.4, 130.3, 130.0, 129.7, 127.4, 127.2, 122.3, 122.2, 122.1, 121.7, 121.6, 121.3, 121.2, 78.9, 78.5, 62.4, 62.0, 47.4, 47.3, 34.0, 33.3, 28.7, 28.1, 22.7, 22.5. HRMS (ESI$^{+}$): Calcd for $C_{22}H_{22}N_2O_2Cl_2$, $[M+H]^{+}$ m/z 417.1131. Found 417.1131.

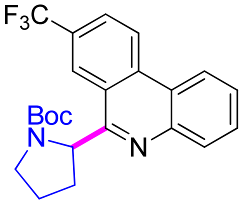


**3ac**

***tert*-Butyl-2-(8-(trifluoromethyl)phenanthridin-6-yl)pyrrolidine-1-carboxylate (3ac).** PE/EtOAc (5:1). Yield 56.3 mg (90%). White solid, mp 164-166 °C. $^{1}$H NMR ($CDCl_3$, 400 MHz) δ 8.81-8.68 (m, 1H), 8.59-8.47 (m, 2H), 8.17-8.08 (m, 1H), 8.06-7.94 (m, 1H), 7.81-7.59 (m, 2H), 5.91-5.72 (m, 1H), 4.01-3.87 (m, 1H), 3.80-3.63 (m, 1H), 2.64-2.47 (m, 1H), 2.19-1.92 (m, 3H), 1.46 (brs, 3.8H), 0.93 (brs, 5.2H). $^{13}$C NMR ($CDCl_3$, 100 MHz) δ 161.5, 160.4, 154.8, 154.5, 144.2, 135.6, 135.3, 130.6, 129.9, 129.6, 129.0 (q, $J$ = 33.6 Hz), 127.2, 127.0, 126.1, 124.1 (q, $J$ = 272.2 Hz), 123.9, 123.8, 123.3, 123.0, 122.6, 122.4, 122.3, 79.3, 79.0, 59.6, 59.4, 47.2, 47.1, 33.6, 32.4, 28.6, 28.1, 23.9, 23.6. HRMS (ESI$^{+}$): Calcd for $C_{23}H_{23}N_2O_2F_3$, $[M+H]^{+}$ m/z 417.1784. Found 417.1782.

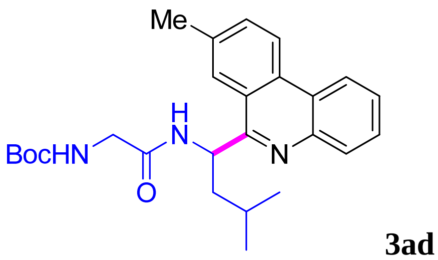

**3ad**

***tert*-Butyl-(2-((3-methyl-1-(8-methylphenanthridin-6-yl)butyl)amino)-2-oxoethyl) carbamate (3ad).** Eluent: PE/EtOAc (2:1). Yield 47.1 mg (72%). White solid, mp 99-101 °C. $^1$H NMR ($CDCl_3$, 400 MHz) δ 8.53 (t, *J* = 8.7 Hz, 2H), 8.10-8.06 (m, 1H), 7.82 (s, 1H), 7.71-7.63 (m, 3H), 6.16-6.08 (m, 1H), 5.24 (s, 1H), 3.95 (s, 2H), 2.62 (s, 3H), 1.90-1.69 (m, 3H), 1.47 (s, 9H), 1.20 (d, *J* = 6.0 Hz, 3H), 0.87 (d, *J* = 6.4 Hz, 3H). $^{13}$C NMR ($CDCl_3$, 100 MHz) δ 168.8, 159.7, 155.9, 142.6, 137.7, 132.6, 131.1, 129.6, 128.3, 126.9, 124.8, 124.0, 123.7, 122.6, 121.9, 80.0, 48.3, 46.8, 44.5, 28.4, 25.3, 23.7, 22.2, 22.0. HRMS (ESI$^+$): Calcd for $C_{26}H_{33}N_3O_3$, $[M+H]^+$ m/z 436.2595. Found 436.2593.

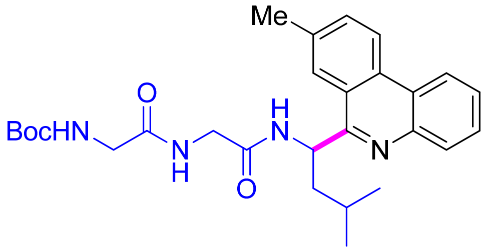

**3ae**

***tert*-Butyl-(2-((2-((3-methyl-1-(8-methylphenanthridin-6-yl)butyl)amino)-2-oxoethyl)amino)-2-oxoethyl)carbamate (3ae).** Eluent: DCM/MeOH (20:1). Yield 50.3 mg (68%). White solid, mp 81-83 °C. $^1$H NMR ($CDCl_3$, 400 MHz) δ 8.51 (dd, $J_1$ = 8.7 Hz, $J_2$ = 11.0 Hz, 2H), 8.09 (d, *J* = 7.8 Hz, 1H), 8.02 (s, 1H), 7.06 (s, 1H), 7.72-7.61 (m, 3H), 6.94 (s, 1H), 6.14-6.06 (m, 1H), 5.26 (s, 1H), 4.13 (d, *J* = 5.0 Hz, 2H), 3.90 (d, *J* = 5.0 Hz, 2H), 2.61 (s, 3H), 1.83-1.72 (m, 3H), 1.44 (s, 9H), 1.19 (d, *J* = 6.0 Hz, 3H), 0.87 (d, *J* = 6.4 Hz, 3H). $^{13}$C NMR ($CDCl_3$, 100 MHz) δ 169.7, 167.9, 159.5, 156.0, 137.7, 132.7, 132.6, 131.2, 129.5, 128.5, 127.0, 124.7, 124.0, 123.6, 122.7, 121.9, 80.2, 48.6, 46.6, 44.2, 43.0, 28.4, 25.4, 23.7, 22.1, 22.0. HRMS (ESI$^+$): Calcd for $C_{28}H_{36}N_4O_4$, $[M+H]^+$ m/z 493.2809. Found 493.2808.

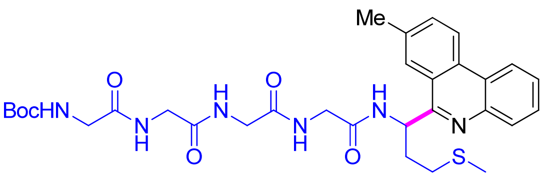


***tert*-Butyl-(5-(8-methylphenanthridin-6-yl)-7,10,13,16-tetraoxo-2-thia-6,9,12,15-tetraazaheptadecan-17-yl)carbamate (3af).** Eluent: DCM/MeOH (10:1). Yield 64.4 mg (69 %). White solid, mp 125-127 °C. $^{1}$H NMR (DMSO-$d_6$, 400 MHz) δ 8.80-8.72 (m, 2H), 8.49 (d, $J$ = 8.7 Hz, 1H), 8.29 (s, 1H), 8.22-8.15 (m, 2H), 8.12-8.03 (m, 2H), 7.82-7.67 (m, 3H), 7.02 (t, $J$ = 6.0 Hz, 1H), 6.10-5.99 (m, 1H), 3.89-3.73 (m, 6H), 3.60 (d, $J$ = 6.0 Hz, 2H), 2.68-2.54 (m, 5H), 2.39-2.28 (m, 1H), 2.21-2.11 (m, 1H), 2.08 (s, 3H), 1.38 (s, 9H). $^{13}$C NMR (DMSO-$d_6$, 100 MHz) δ 170.3, 169.8, 169.6, 168.9, 159.8, 156.4, 142.8, 138.1, 133.2, 130.9, 129.9, 129.0, 127.6, 125.3, 124.4, 124.1, 123.4, 123.0, 78.6, 49.4, 43.9, 42.6, 34.4, 30.7, 28.7, 22.0, 15.2. HRMS (ESI$^+$): Calcd for $C_{31}H_{40}N_6O_6S$, [M+H]$^+$ 625.2803. Found m/z 625.2794.

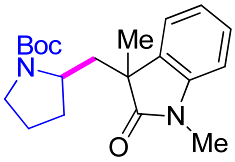

**5a**

***tert*-Butyl-2-((1,3-dimethyl-2-oxoindolin-3-yl)methyl)pyrrolidine-1-carboxylate (5a).** Eluent: PE/EtOAc (5:1). Yield 33.7 mg (65%). Clear oil. $^{1}$H NMR ($CDCl_3$, 400 MHz) δ 7.26 (t, $J$ = 7.6 Hz, 1H), 7.19 (d, $J$ = 7.2 Hz, 1H), 7.05 (dd, $J_1$ = 7.6 Hz, $J_2$ = 7.2 Hz, 1H), 6.83 (d, $J$ = 7.6 Hz, 1H), 3.88-3.71 (m, 1H), 3.39-2.98 (m, 5H), 2.61-1.92 (m, 2H), 1.80-1.70 (m, 1H), 1.67-1.53 (m, 2H), 1.45 (s, 9H), 1.40 (s, 3H), 1.22-1.13 (m, 1H). $^{13}$C NMR ($CDCl_3$, 100 MHz) δ 179.8, 154.5, 143.1, 134.6, 127.9, 122.8, 122.4, 108.1, 79.6, 54.6, 46.7, 45.6, 42.3, 31.1, 28.6, 26.3, 24.8, 23.0. HRMS (ESI$^+$): Calcd for $C_{20}H_{28}N_2O_3$, [M+H]$^+$ m/z 345.2173. Found 345.2169.

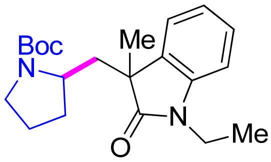

**5b**

***tert*-Butyl-2-((1-ethyl-3-methyl-2-oxoindolin-3-yl)methyl)pyrrolidine-1-carboxyla**

**te (5b).** Eluent: PE/EtOAc (5:1). Yield 34.0 mg (63%). Clear oil. $^{1}$H NMR ($CDCl_3$, 400 MHz) δ 7.23 (dd, $J_1$ = 7.6 Hz, $J_2$ = 7.2 Hz, 1H), 7.17 (d, $J$ = 7.2 Hz, 1H), 7.02 (t, $J$ = 7.6 Hz, 1H), 6.82 (d, $J$ = 7.6 Hz, 1H), 3.84-3.67 (m, 3H), 3.35-2.93 (m, 2H), 2.48-1.86 (m, 2H), 1.77-1.68 (m, 1H), 1.62-1.51 (m, 2H), 1.43 (s, 9H), 1.37 (s, 3H), 1.23 (t, $J$ = 7.2 Hz, 3H), 1.15-1.06 (m, 1H). $^{13}$C NMR ($CDCl_3$, 100 MHz) δ 179.3, 154.4, 142.0, 134.9, 127.8, 123.0, 122.2, 108.2, 79.6, 54.6, 46.6, 45.6, 42.3, 34.6, 30.9, 28.6, 24.8, 23.0, 12.8. HRMS (ESI$^+$): Calcd for $C_{21}H_{30}N_2O_3$, $[M+H]^+$ m/z 359.2329. Found 359.2324.

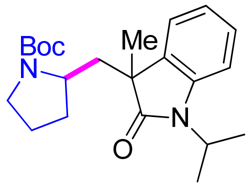


**5c**

***tert*-Butyl-2-((1-isopropyl-3-methyl-2-oxoindolin-3-yl)methyl)pyrrolidine-1-carboxylate (5c).** Eluent: PE/EtOAc (5:1). Yield 32.5 mg (58%). Clear oil. $^{1}$H NMR ($CDCl_3$, 400 MHz) δ 7.25-7.17 (m, 2H), 7.05-6.98 (m, 2H), 4.71-4.62 (m, 1H), 3.92-3.71 (m, 1H), 3.35-2.91 (m, 2H), 2.54-2.21 (m, 1H), 1.90-1.66 (m, 2H), 1.64-1.51 (m, 2H), 1.47 (d, $J$ = 6.9 Hz, 6H), 1.46 (s, 9H), 1.38 (s, 3H), 1.13-1.05 (m, 1H). $^{13}$C NMR ($CDCl_3$, 100 MHz) δ 179.3, 154.4, 141.6, 135.1, 127.5, 123.1, 121.8, 109.8, 79.4, 54.6, 46.3, 45.6, 43.5, 42.5, 30.7, 28.6, 24.9, 23.0, 19.6, 19.4. HRMS (ESI$^+$): Calcd for $C_{22}H_{32}N_2O_3$, $[M+H]^+$ m/z 373.2486. Found 373.2485.

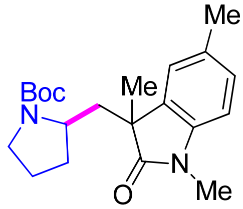


**5d**

***tert*-Butyl-2-((1,3,5-trimethyl-2-oxoindolin-3-yl)methyl)pyrrolidine-1-carboxylate (5d).** Eluent: PE/EtOAc (5:1). Yield 39.9 mg (74%). Clear oil. $^{1}$H NMR ($CDCl_3$, 400 MHz) δ 7.06 (d, $J$ = 7.2 Hz, 1H), 7.00 (s, 1H), 6.71 (d, $J$ = 7.2 Hz, 1H), 3.88-3.74 (m, 1H), 3.41-3.23 (m, 1H), 3.22-2.99 (m, 4H), 2.50-2.26 (m, 5H), 1.79-1.69 (m, 1H), 1.68-1.55 (m, 2H), 1.45 (s, 9H), 1.38 (s, 3H), 1.26-1.18 (m, 1H). $^{13}$C NMR ($CDCl_3$, 100 MHz) δ 179.7, 154.3, 140.7, 134.8, 131.9, 128.1, 123.7, 107.8, 79.5, 54.6, 46.8,

45.7, 42.4, 31.2, 28.6, 26.3, 24.8, 23.3, 21.2. HRMS (ESI$^+$): Calcd for $C_{21}H_{30}N_2O_3$, $[M+H]^+$ m/z 359.2329. Found 359.2329.

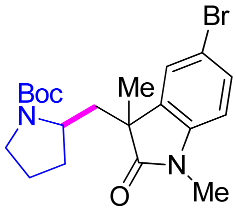

**5e**

***tert*-Butyl-2-((5-bromo-1,3-dimethyl-2-oxoindolin-3-yl)methyl)pyrrolidine-1-carboxylate (5e).** Eluent: PE/EtOAc (5:1). Yield 39.5 mg (62%). Clear oil. $^1$H NMR ($CDCl_3$, 400 MHz) δ 7.39 (d, *J* = 8.2 Hz, 1H), 7.30 (s, 1H), 6.71 (d, *J* = 8.2 Hz, 1H), 3.84-3.73 (m, 1H), 3.42-3.23 (m, 1H), 3.22-2.99 (m, 4H), 2.49-2.23 (m, 2H), 1.79-1.71 (m, 1H), 1.70-1.56 (m, 2H), 1.44 (s, 9H), 1.39 (s, 3H), 1.26-1.18 (m, 1H). $^{13}$C NMR ($CDCl_3$, 100 MHz) δ 179.3, 154.4, 142.2, 136.7, 130.7, 126.2, 115.1, 109.5, 79.7, 54.4, 47.0, 45.7, 42.3, 31.5, 28.6, 26.4, 24.8, 23.3. HRMS (ESI$^+$): Calcd for $C_{20}H_{27}N_2O_3Br$, $[M+H]^+$ m/z 425.1260. Found 425.1262.

## References


1. Kachkovskyi, G., Faderl, C. & Reiser, O. *Adv. Synth. Catal.* **355**, 2240-2248 (2013).
2. Wang, Q., Dong, X., Xiao, T. & Zhou, L. *Org. Lett.* **15**, 4846-4849 (2013).

The $^{1}$H and $^{13}$C NMR spectra of compounds **3a-af** and **5a-e**

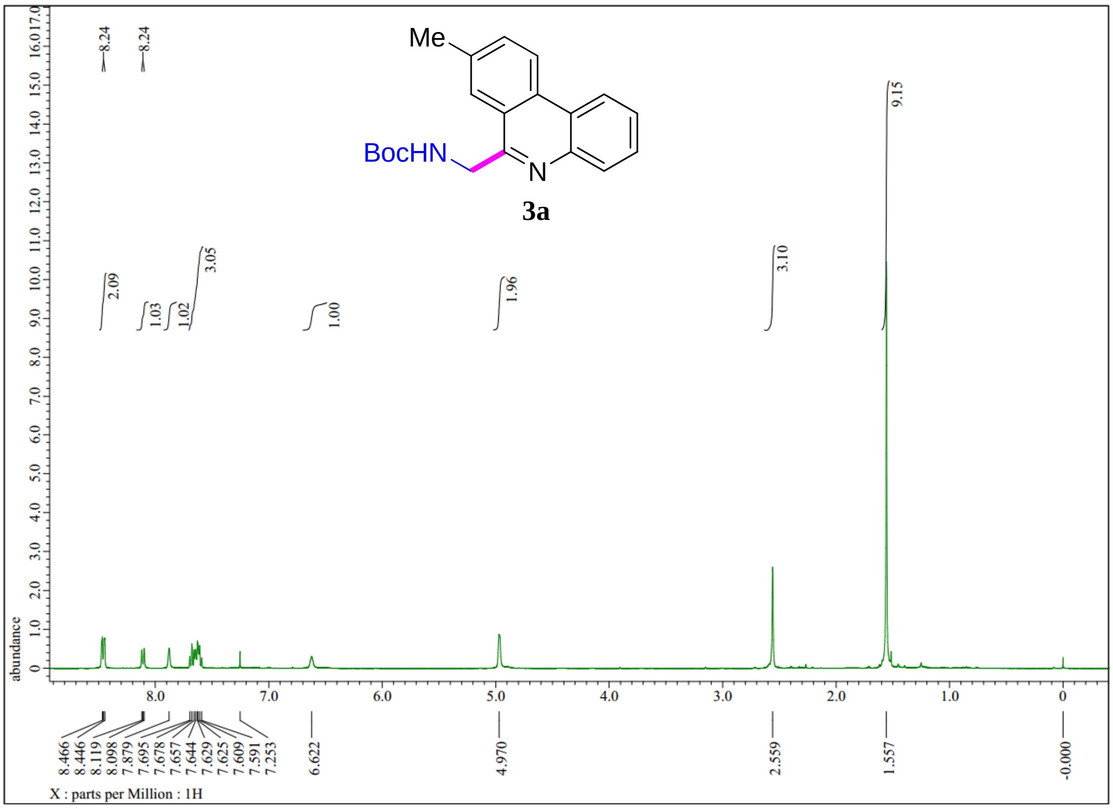

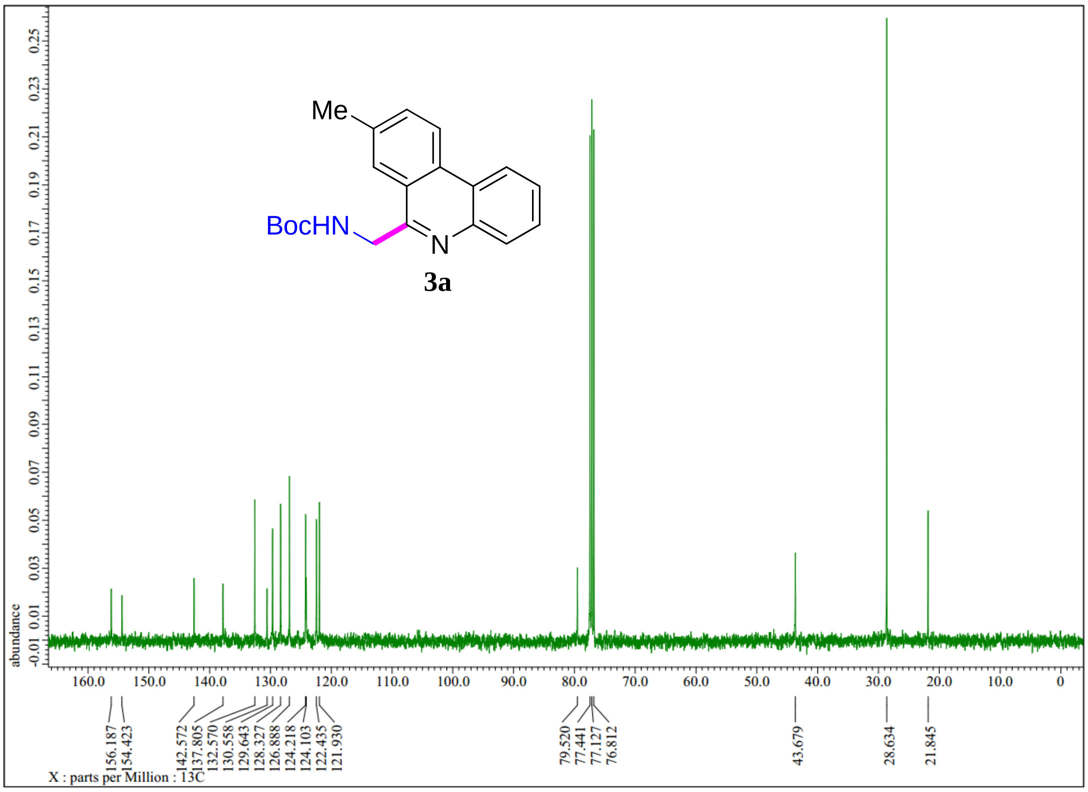

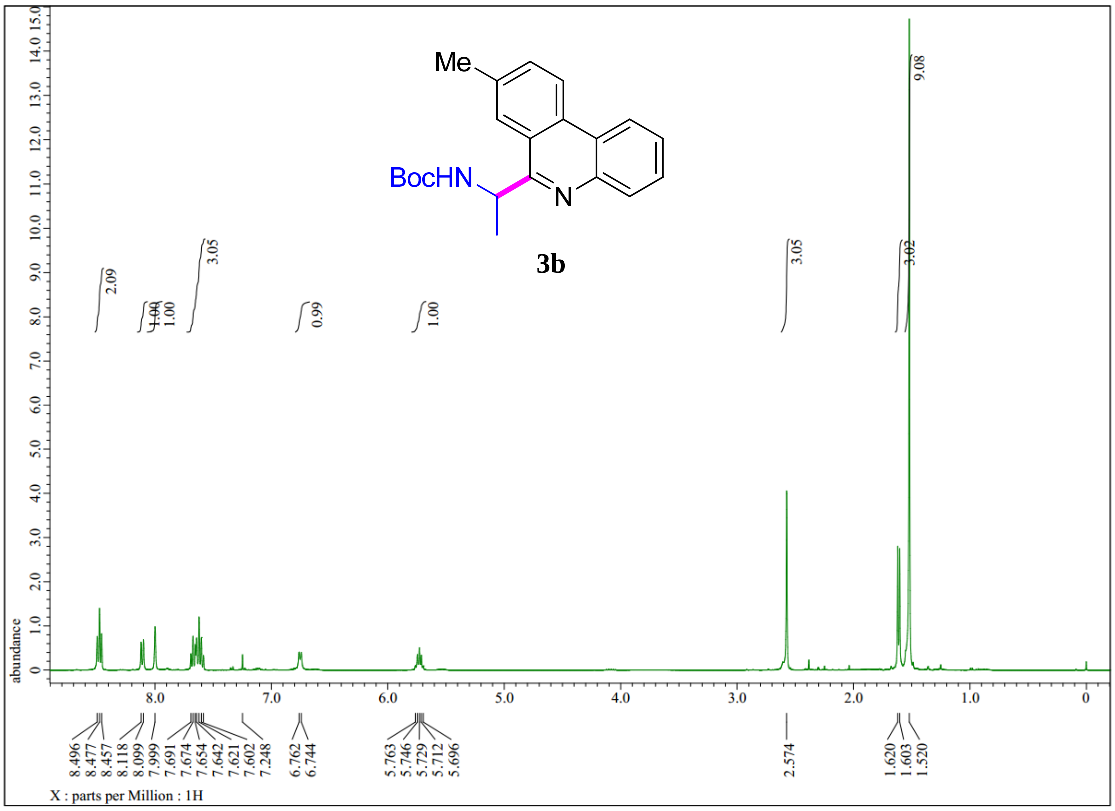
Me
BocHN
N
3b
X : parts per Million : 1H
abundance

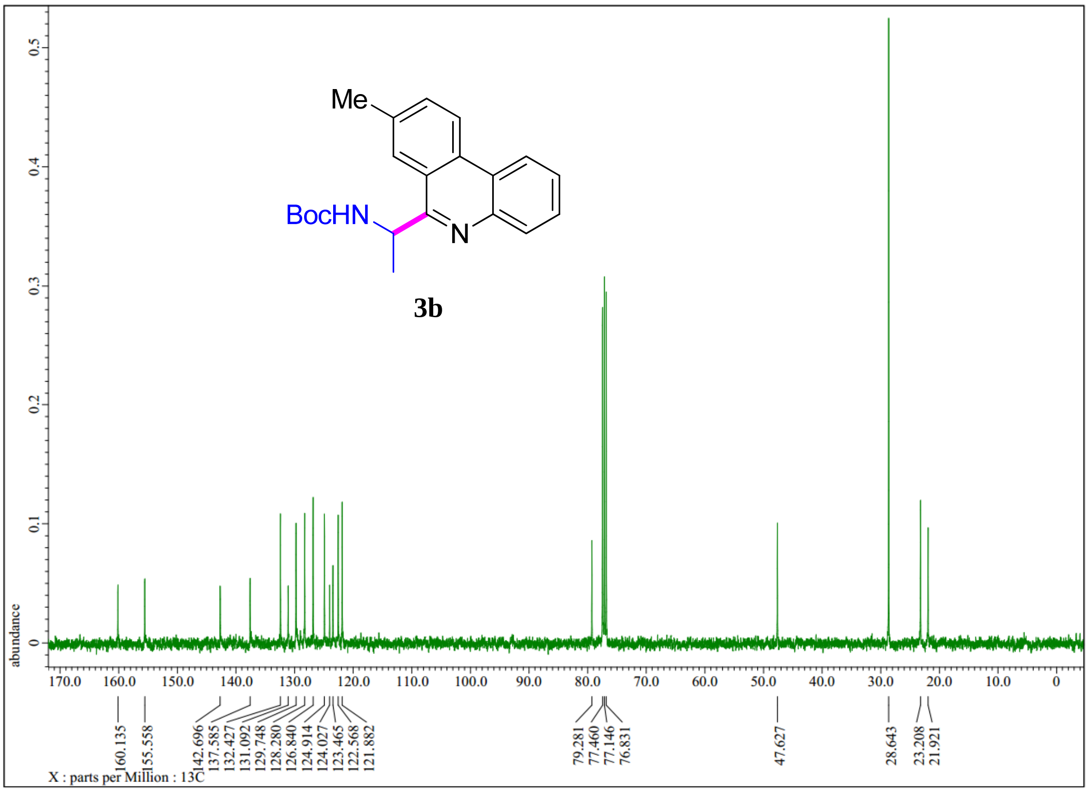
Me
BocHN
N
3b
X : parts per Million : 13C
abundance

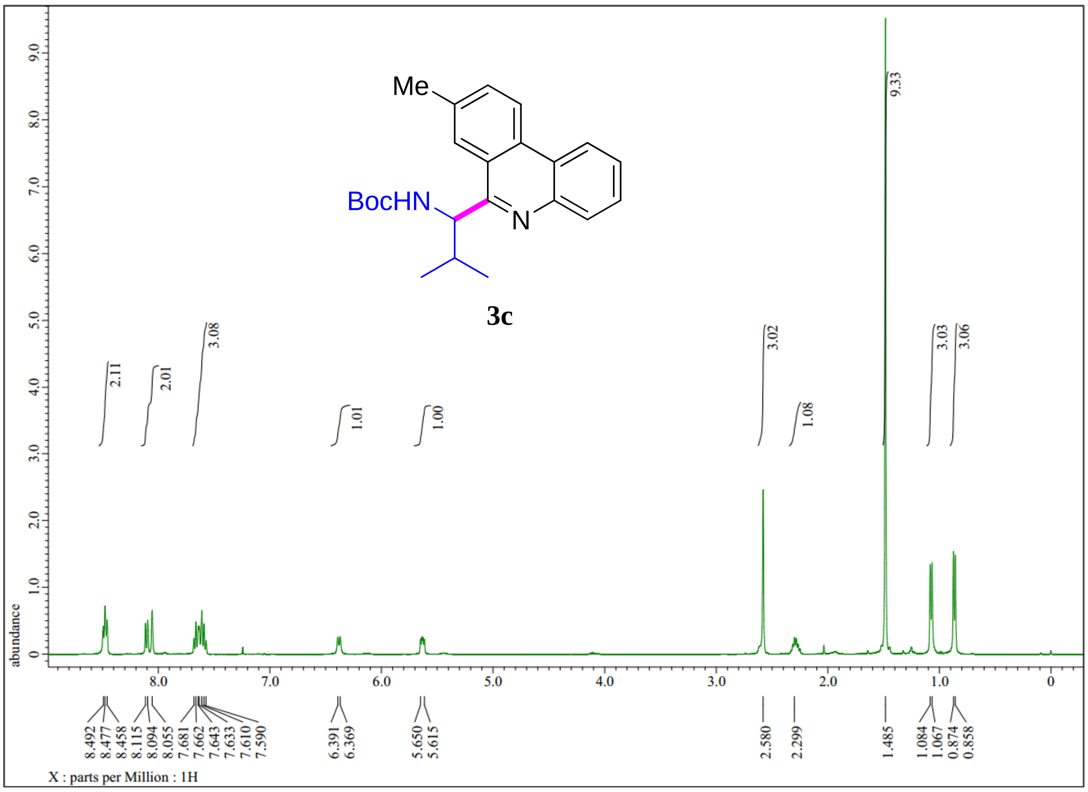
Me
BocHN
N
3c
abundance
X : parts per Million : 1H
2.11
2.01
3.08
1.01
1.00
3.02
1.08
9.33
3.03
3.06
8.492
8.477
8.458
8.115
8.094
8.055
7.681
7.662
7.643
7.633
7.610
7.590
6.391
6.369
5.650
5.615
2.580
2.299
1.485
1.084
1.067
0.874
0.858

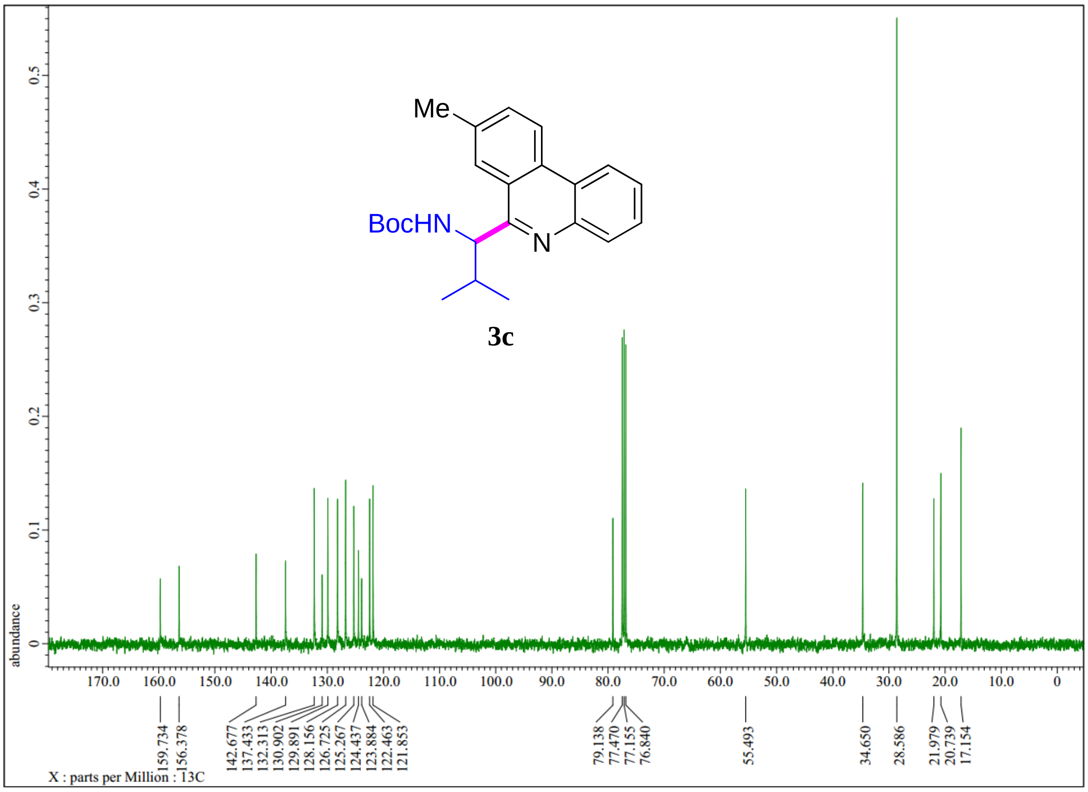
Me
BocHN
N
3c
abundance
X : parts per Million : 13C
159.734
156.378
142.677
137.433
132.313
130.902
129.891
128.156
126.725
125.267
124.437
123.884
122.463
121.853
79.138
77.470
77.155
76.840
55.493
34.650
28.586
21.979
20.739
17.154

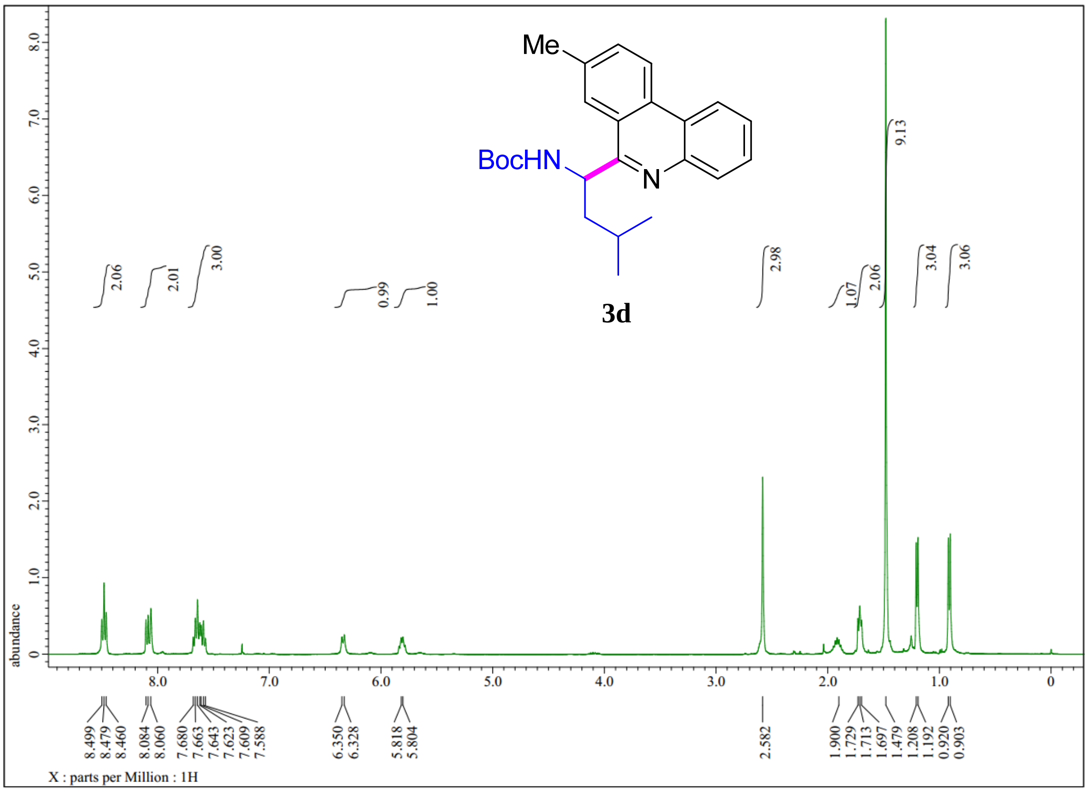
Me
BocHN
N
3d
abundance
X : parts per Million : 1H
2.06
2.01
3.00
0.99
1.00
2.98
1.07
2.06
9.13
3.04
3.06
8.499
8.479
8.460
8.084
8.060
7.680
7.663
7.643
7.623
7.609
7.588
6.350
6.328
5.818
5.804
2.582
1.900
1.729
1.713
1.697
1.479
1.208
1.192
0.920
0.903

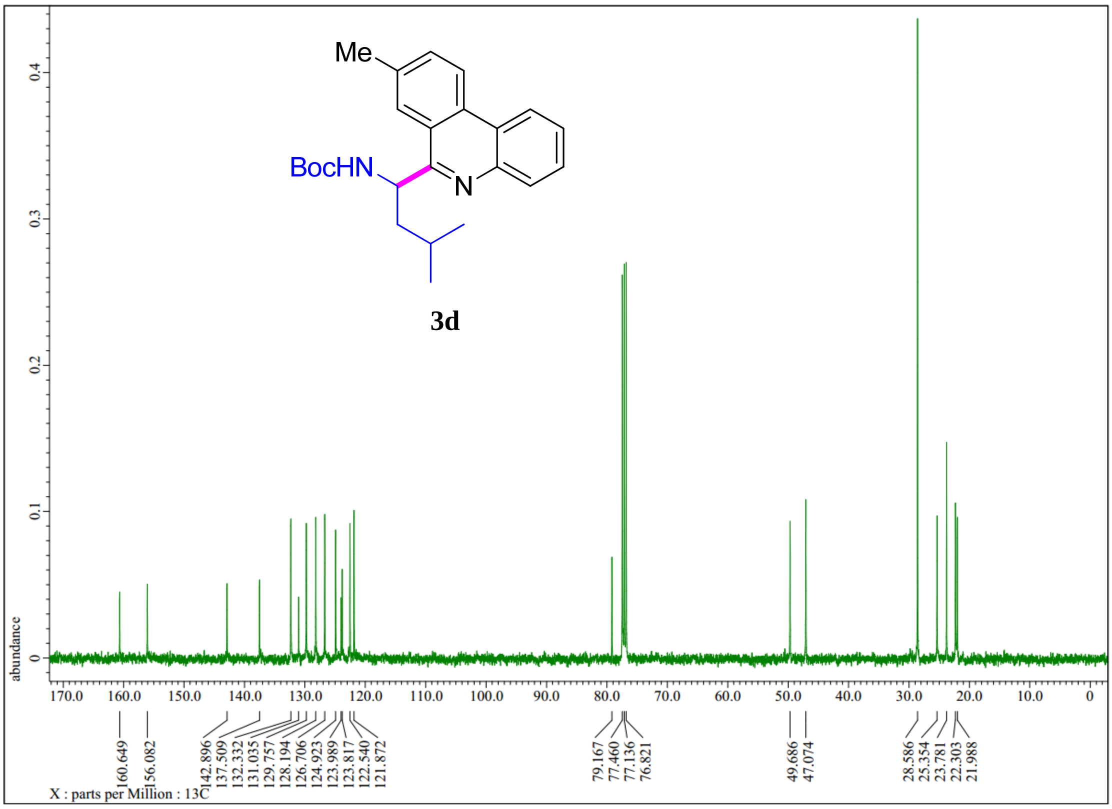
Me
BocHN
N
3d
abundance
X : parts per Million : 13C
160.649
156.082
142.896
137.509
132.332
131.035
129.757
128.194
126.706
124.923
123.989
123.817
122.540
121.872
79.167
77.460
77.136
76.821
49.686
47.074
28.586
25.354
23.781
22.303
21.988

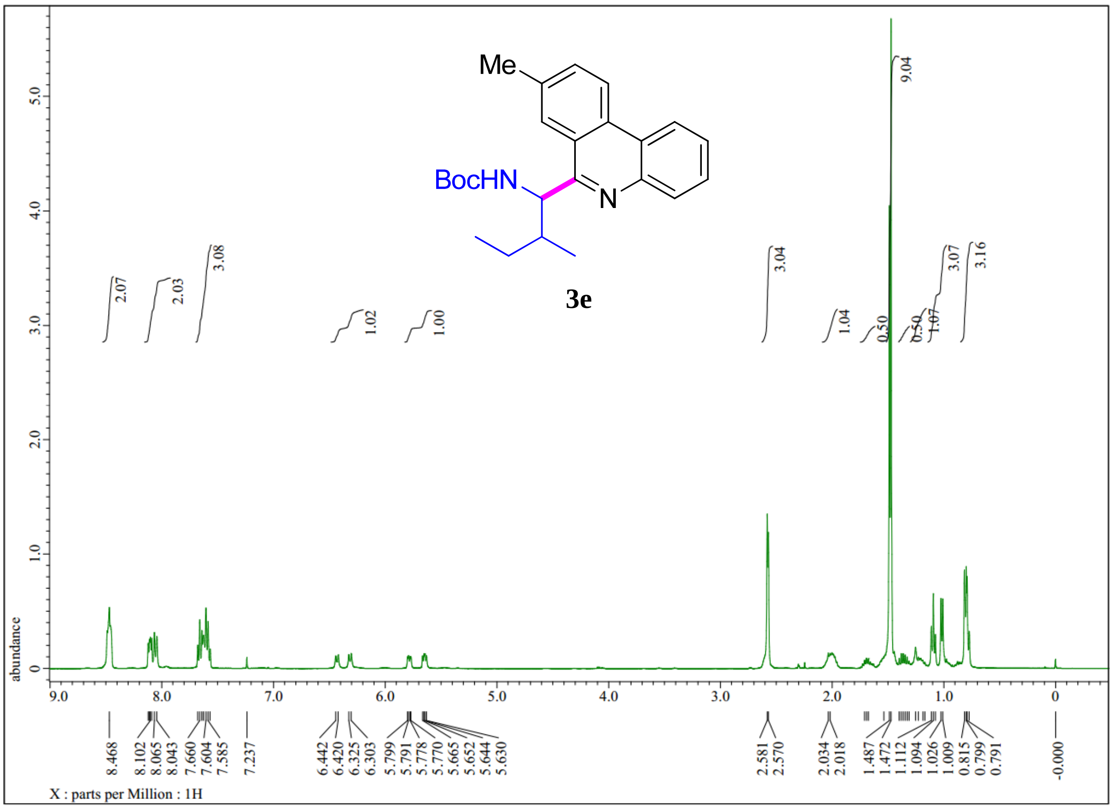
Me
BocHN
N
3e
X : parts per Million : 1H
abundance

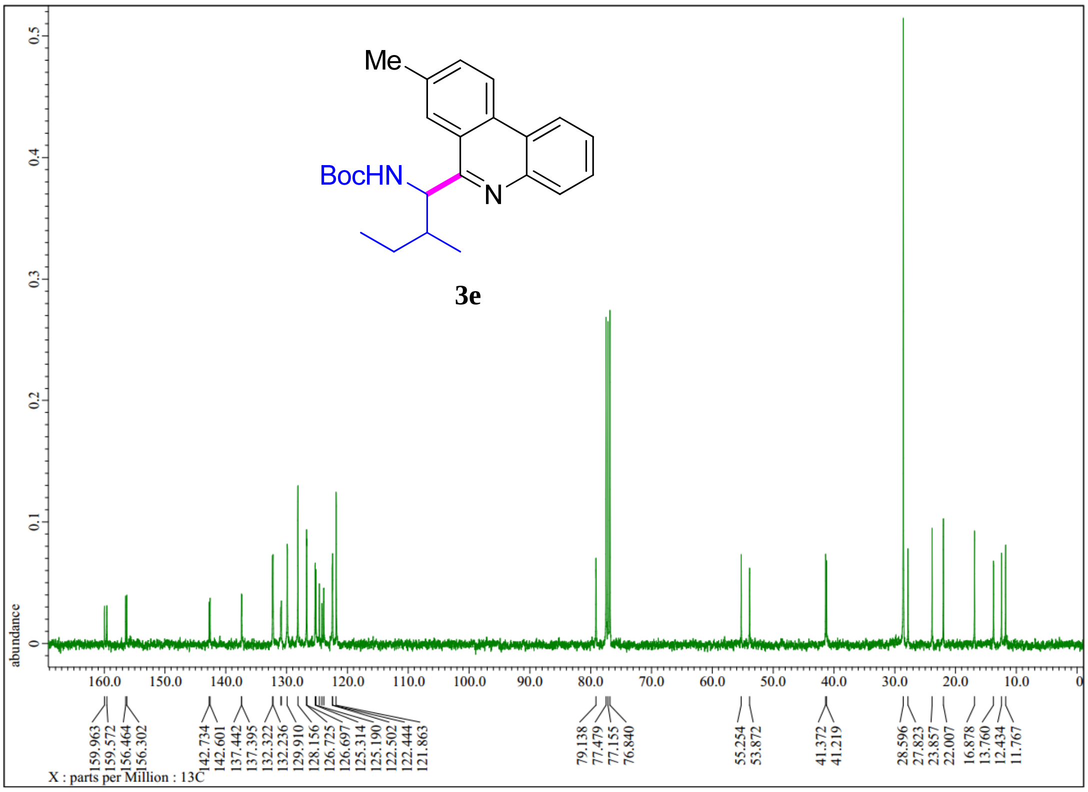
Me
BocHN
N
3e
X : parts per Million : 13C
abundance

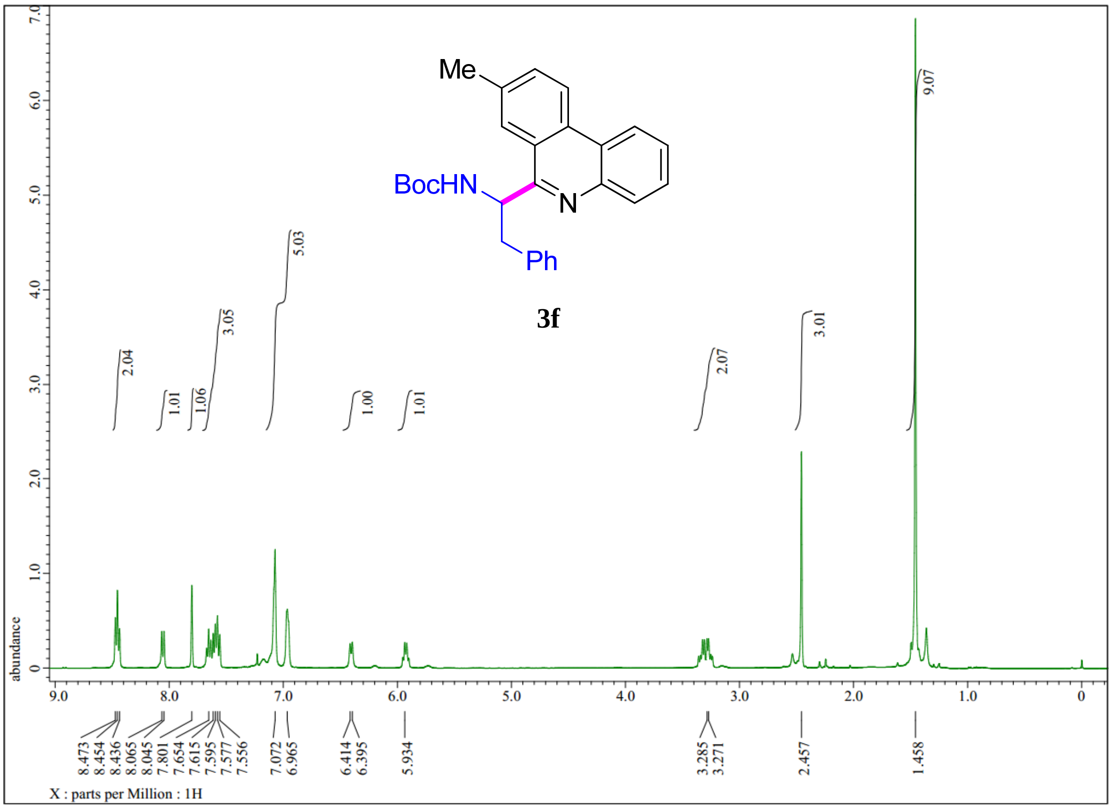
Me
BocHN
N
Ph
3f
X : parts per Million : 1H
abundance

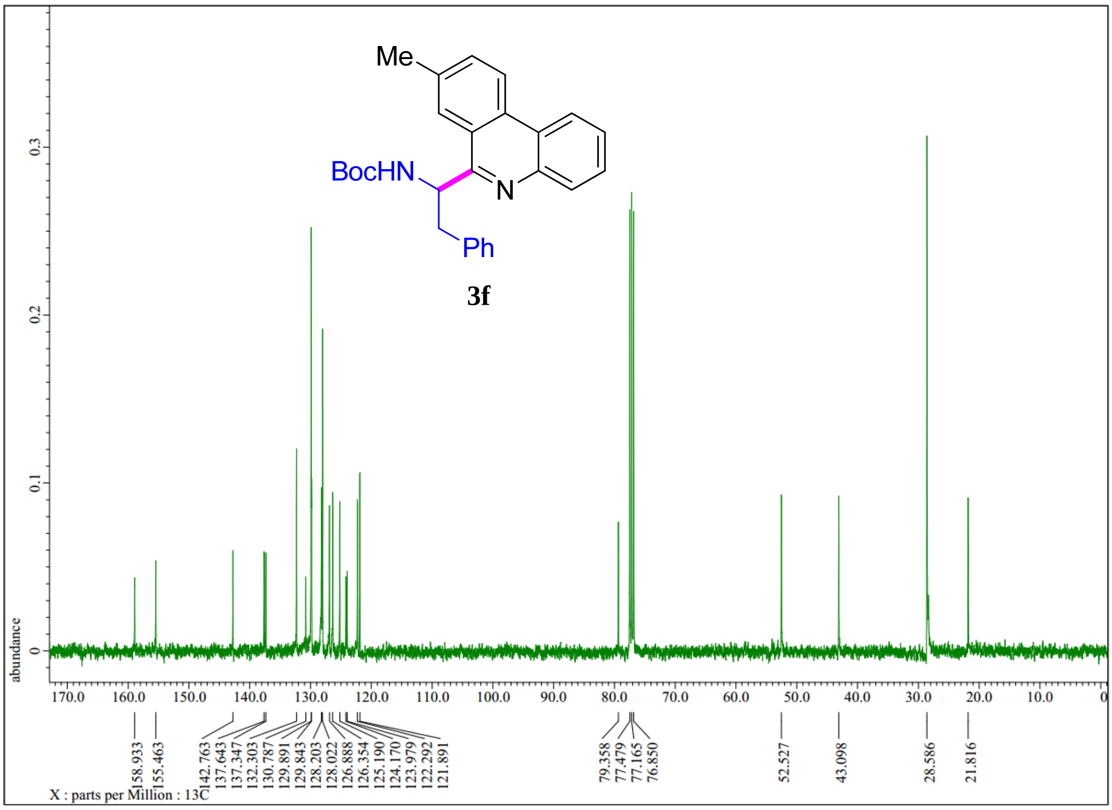
Me
BocHN
N
Ph
3f
X : parts per Million : 13C
abundance

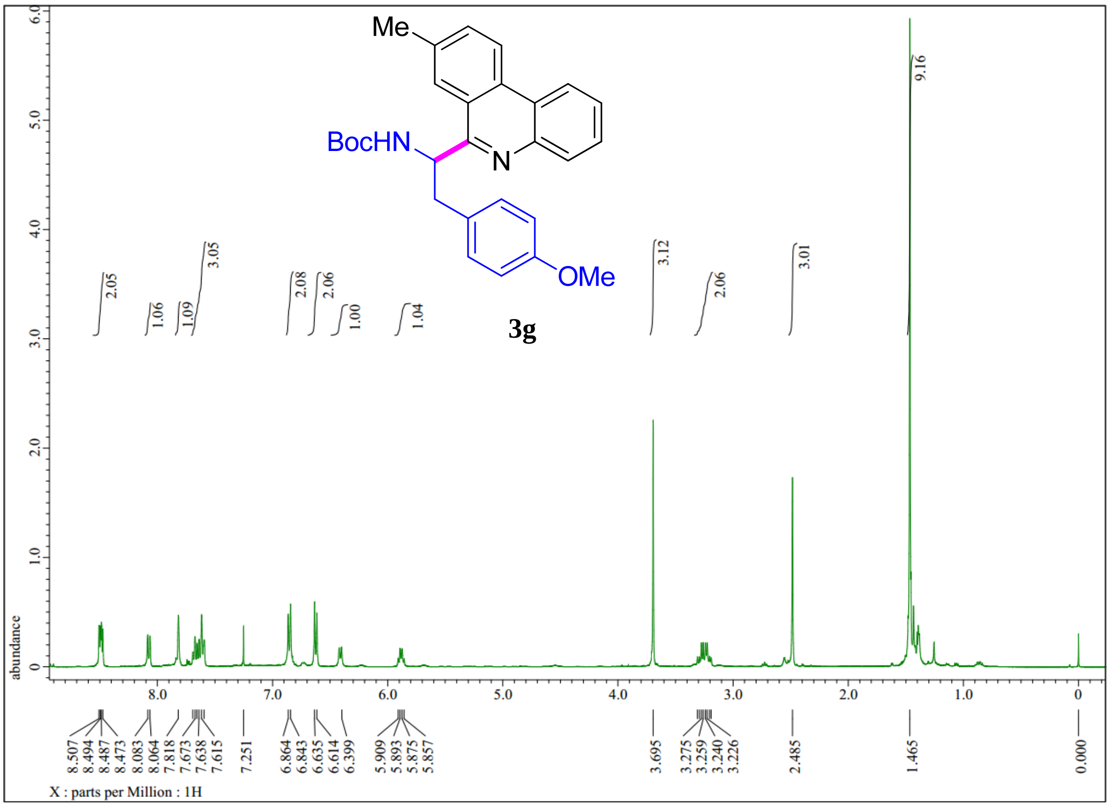

Me
BocHN
N
OMe
3g
abundance
X : parts per Million : 1H


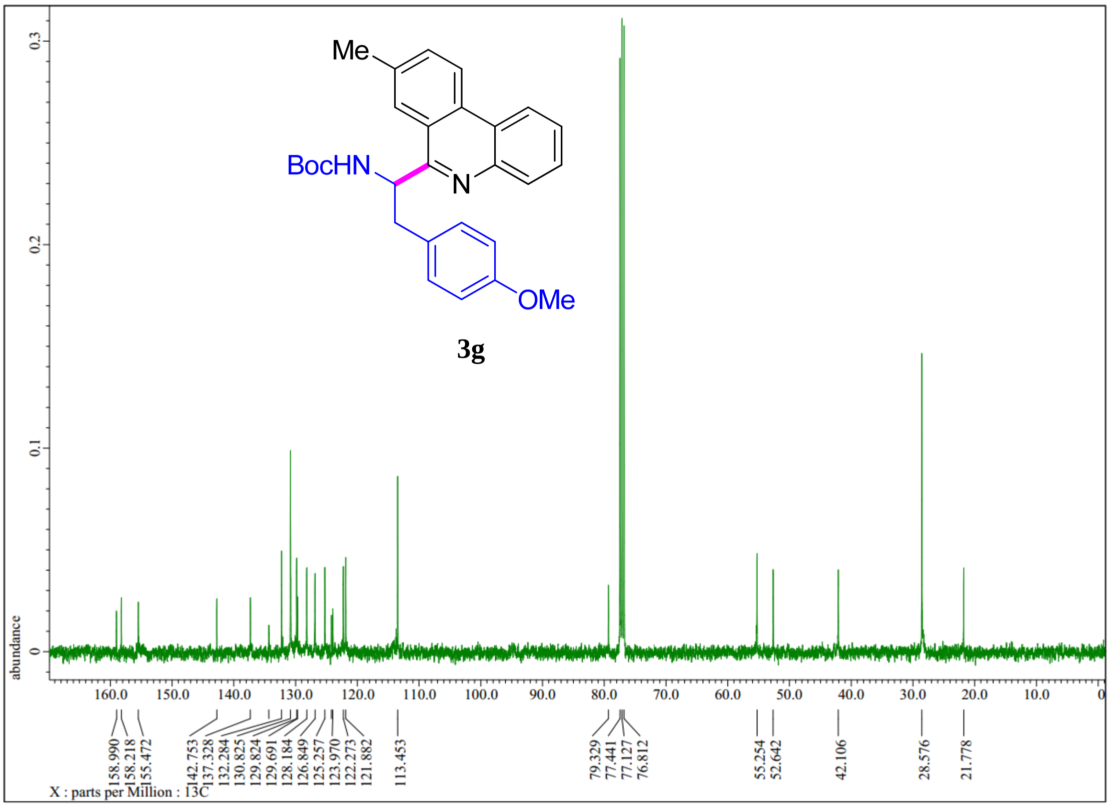

Me
BocHN
N
OMe
3g
abundance
X : parts per Million : 13C

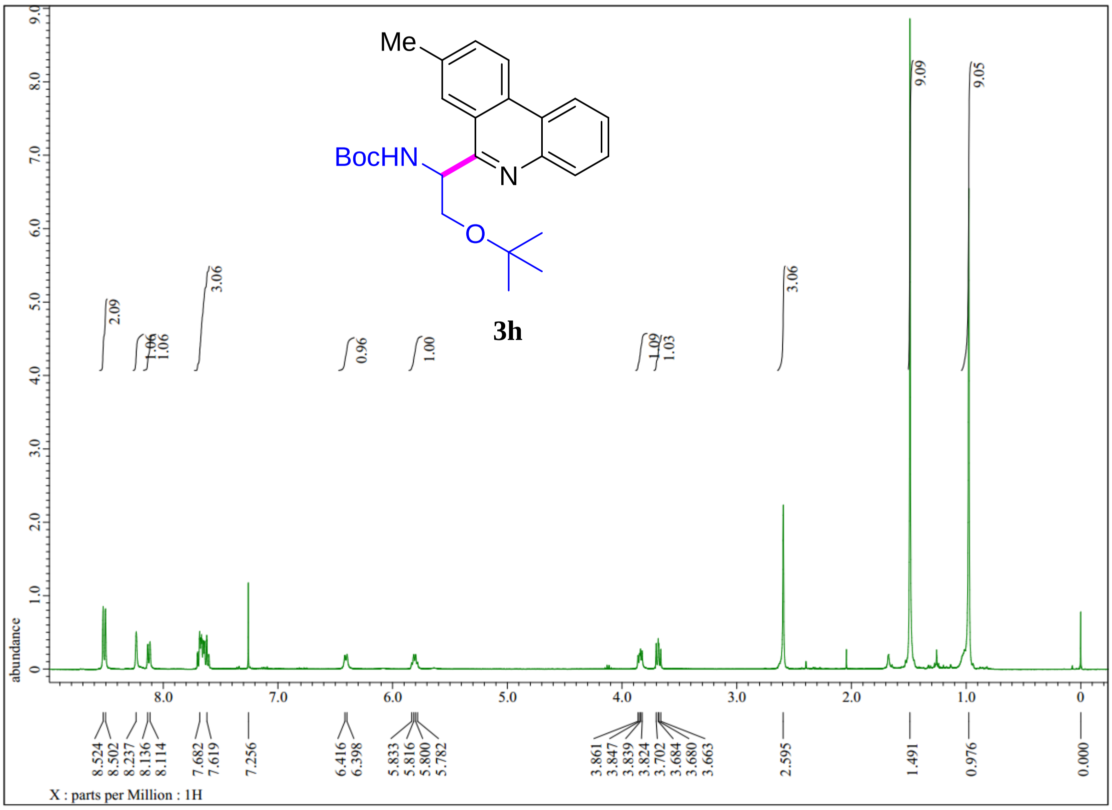
Me
BocHN
N
O
3h
abundance
X : parts per Million : 1H
8.524
8.502
8.237
8.136
8.114
7.682
7.619
7.256
6.416
6.398
5.833
5.816
5.800
5.782
3.861
3.847
3.839
3.824
3.702
3.684
3.680
3.663
2.595
1.491
0.976
0.000
2.09
1.06
1.06
3.06
0.96
1.00
1.09
1.03
3.06
9.09
9.05

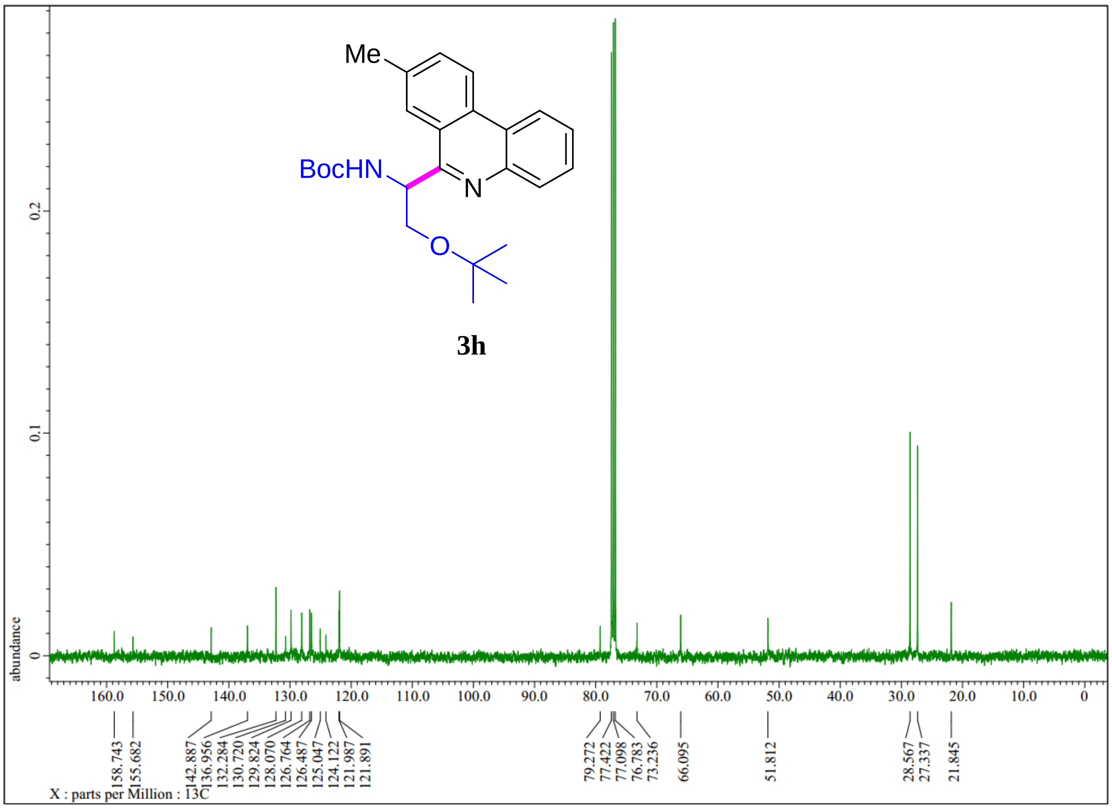
Me
BocHN
N
O
3h
abundance
X : parts per Million : 13C
158.743
155.682
142.887
136.956
132.284
130.720
129.824
128.070
126.764
126.487
125.047
124.122
121.987
121.891
79.272
77.422
77.098
76.783
73.236
66.095
51.812
28.567
27.337
21.845

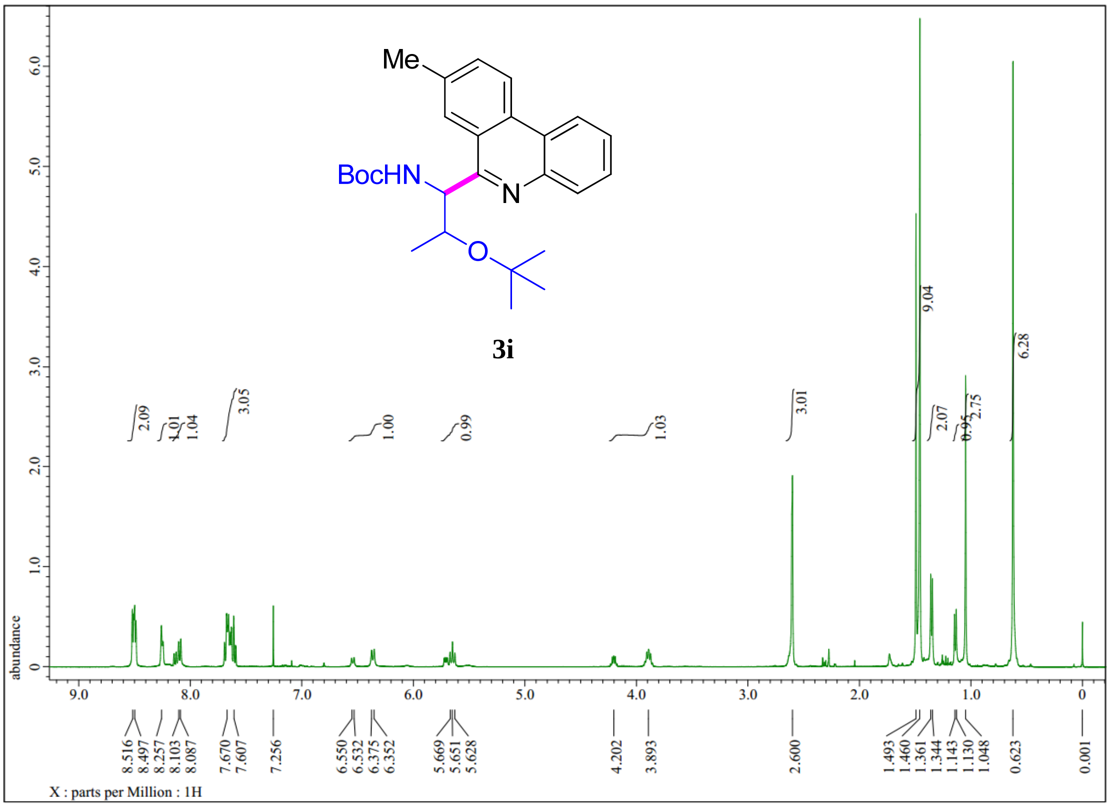
Me
BocHN
N
O
3i
abundance
X : parts per Million : 1H
2.09
1.01
1.04
3.05
1.00
0.99
1.03
3.01
9.04
2.07
0.95
2.75
6.28
8.516
8.497
8.257
8.103
8.087
7.670
7.607
7.256
6.550
6.532
6.375
6.352
5.669
5.651
5.628
4.202
3.893
2.600
1.493
1.460
1.361
1.344
1.143
1.130
1.048
0.623
0.001

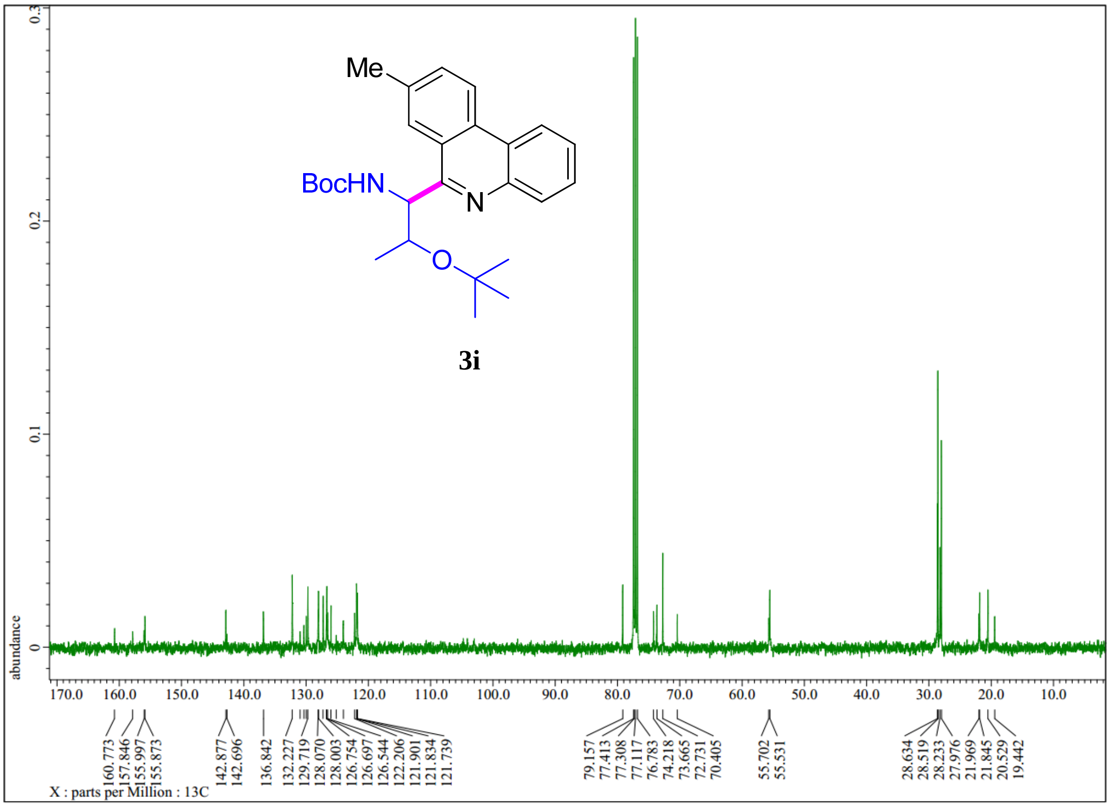
Me
BocHN
N
O
3i
abundance
X : parts per Million : 13C
160.773
157.846
155.997
155.873
142.877
142.696
136.842
132.227
129.719
128.070
128.003
126.754
126.697
126.544
122.206
121.901
121.834
121.739
79.157
77.413
77.308
77.117
76.783
74.218
73.665
72.731
70.405
55.702
55.531
28.634
28.519
28.233
27.976
21.969
21.845
20.529
19.442

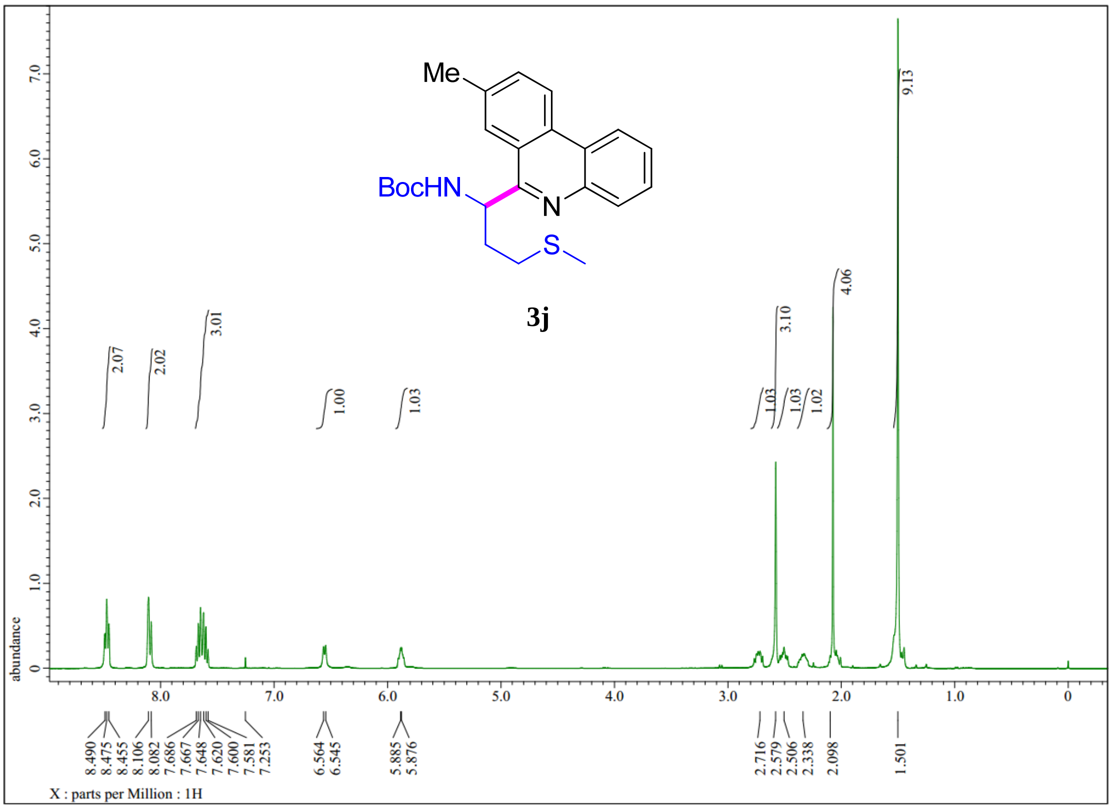
Me
BocHN
N
S
3j
X : parts per Million : 1H
abundance

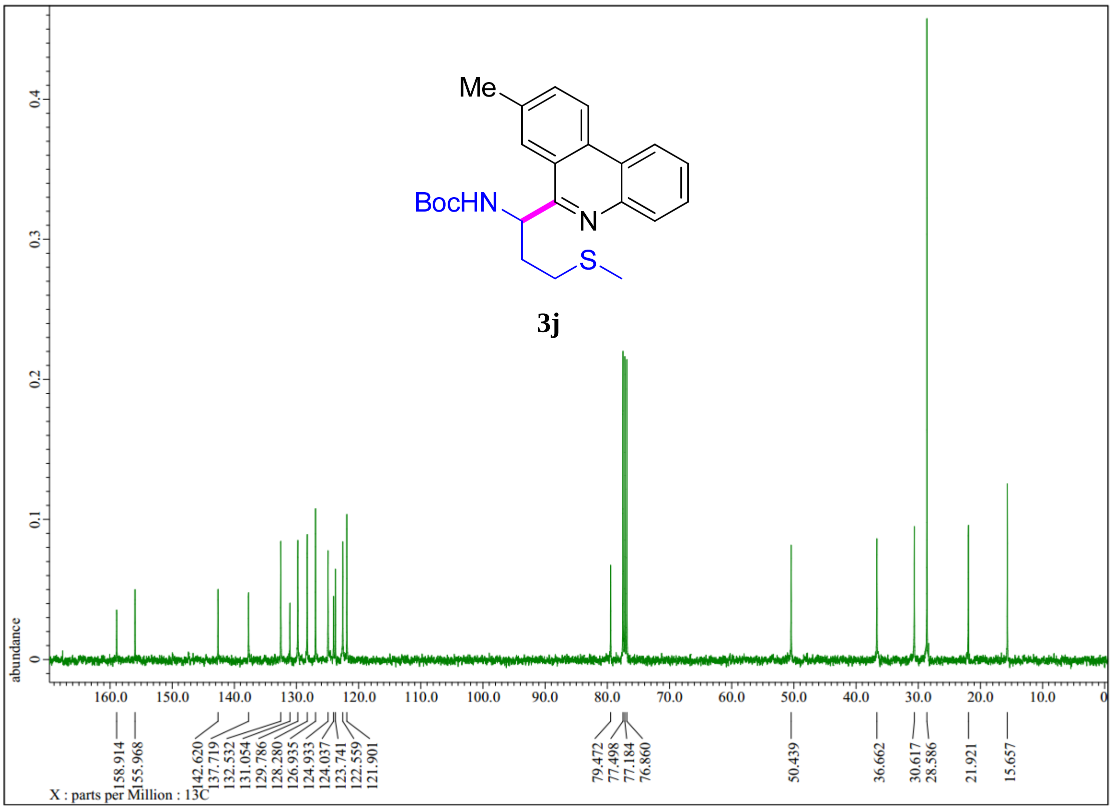
Me
BocHN
N
S
3j
X : parts per Million : 13C
abundance

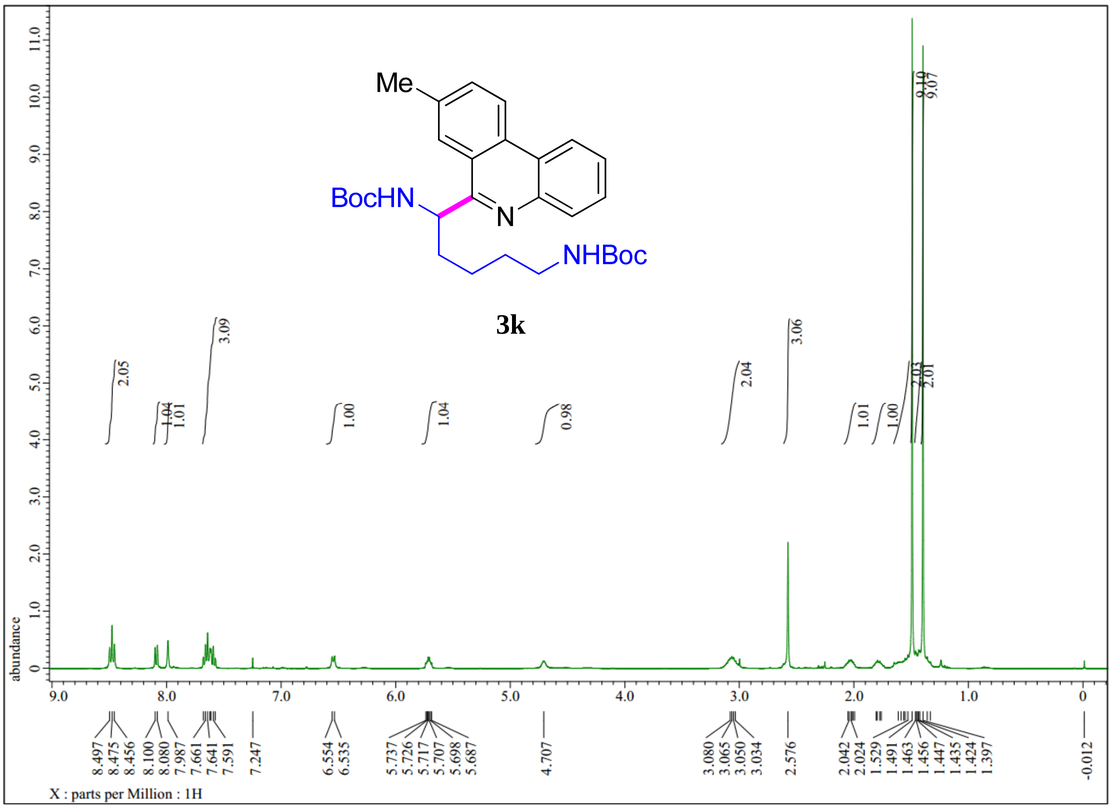
Me
BocHN
N
NHBoc
3k
abundance
X : parts per Million : 1H

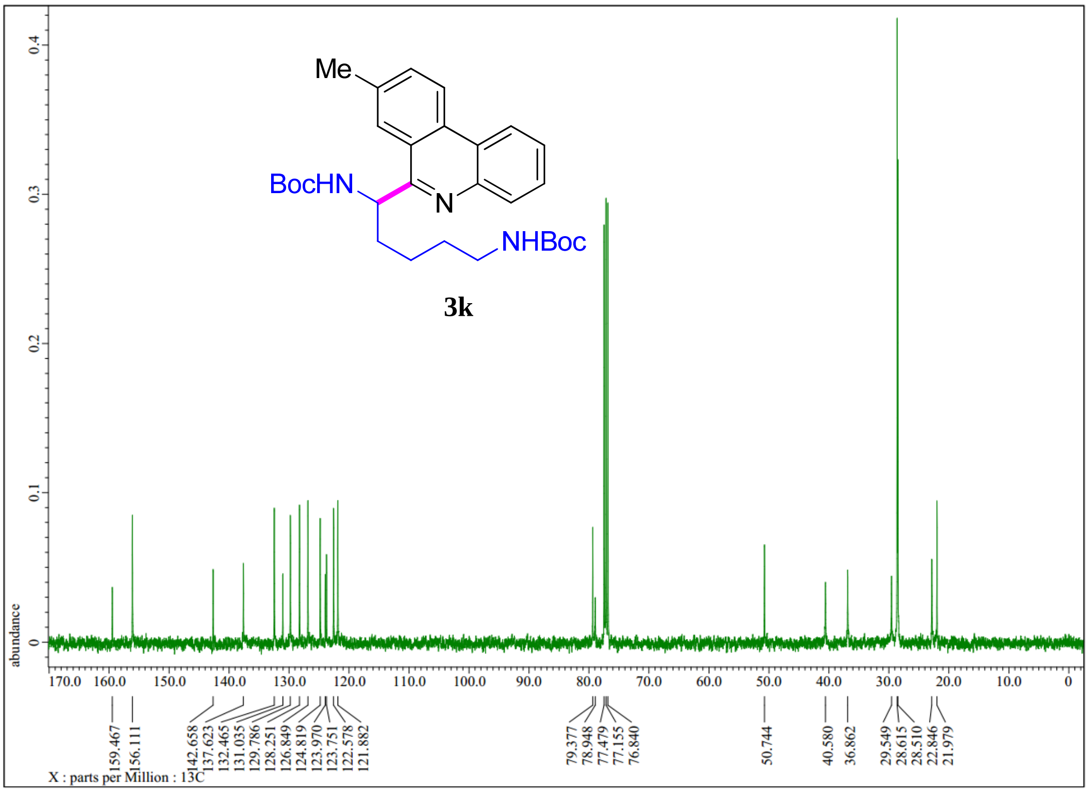
Me
BocHN
N
NHBoc
3k
abundance
X : parts per Million : 13C

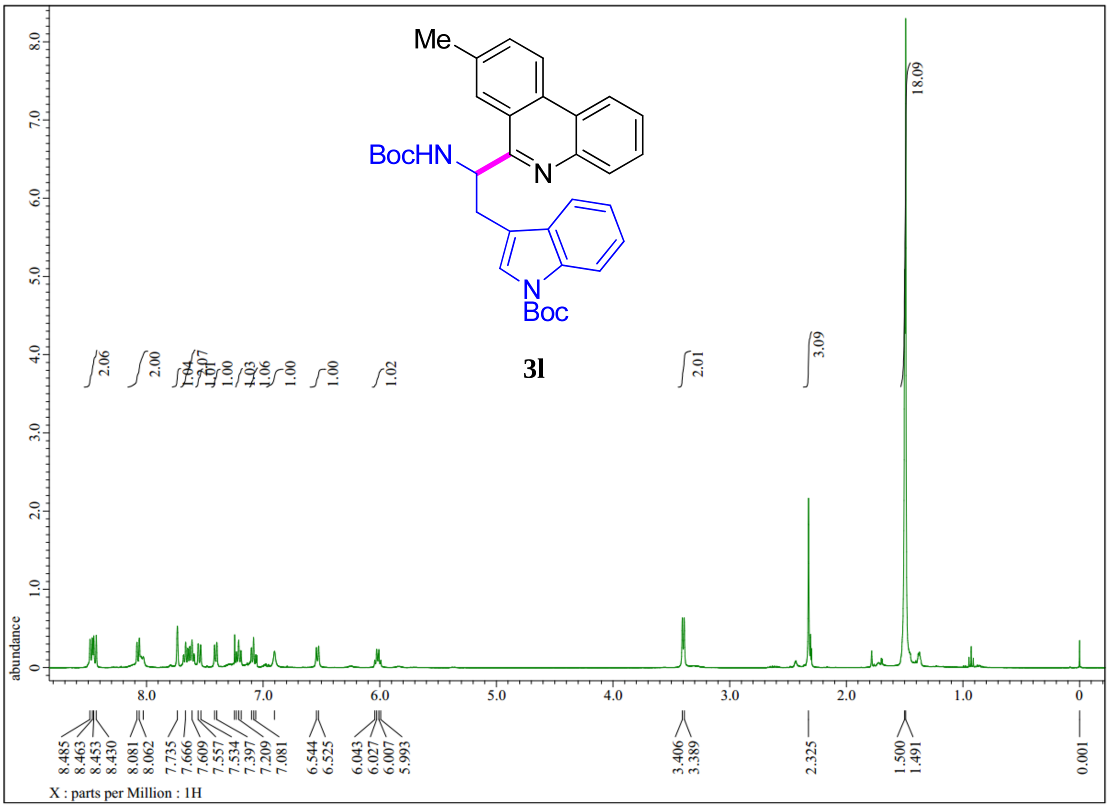

3l

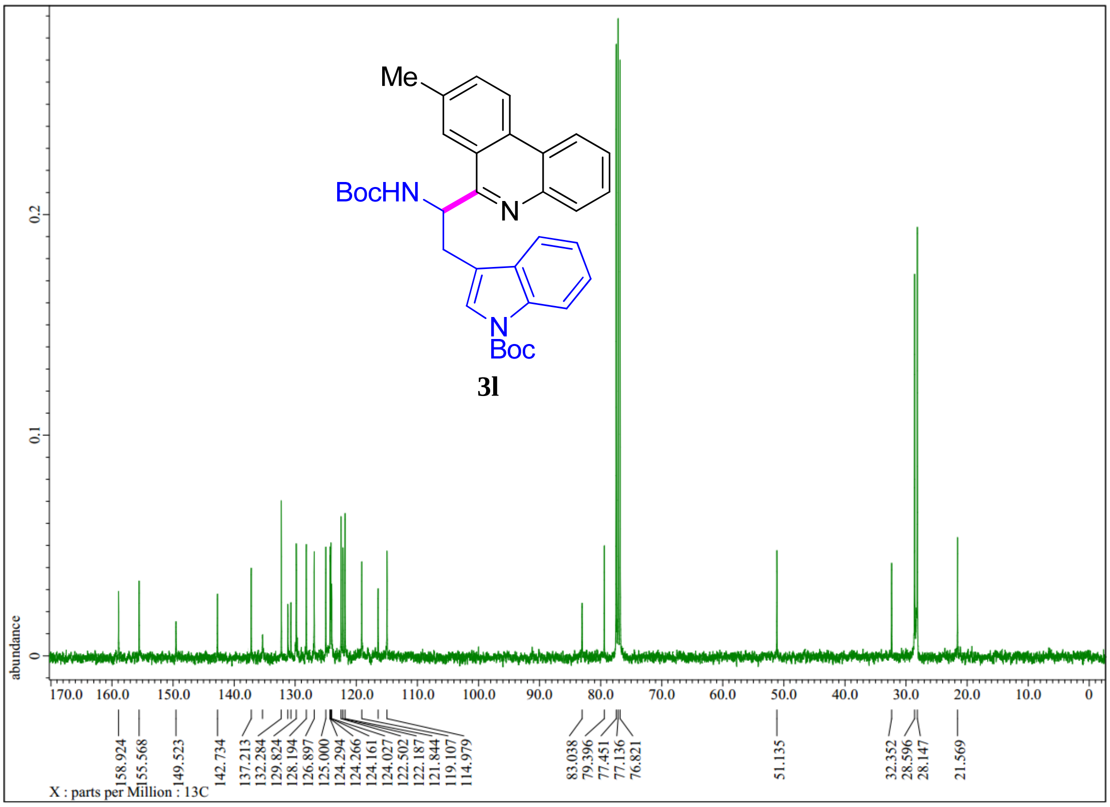

3l

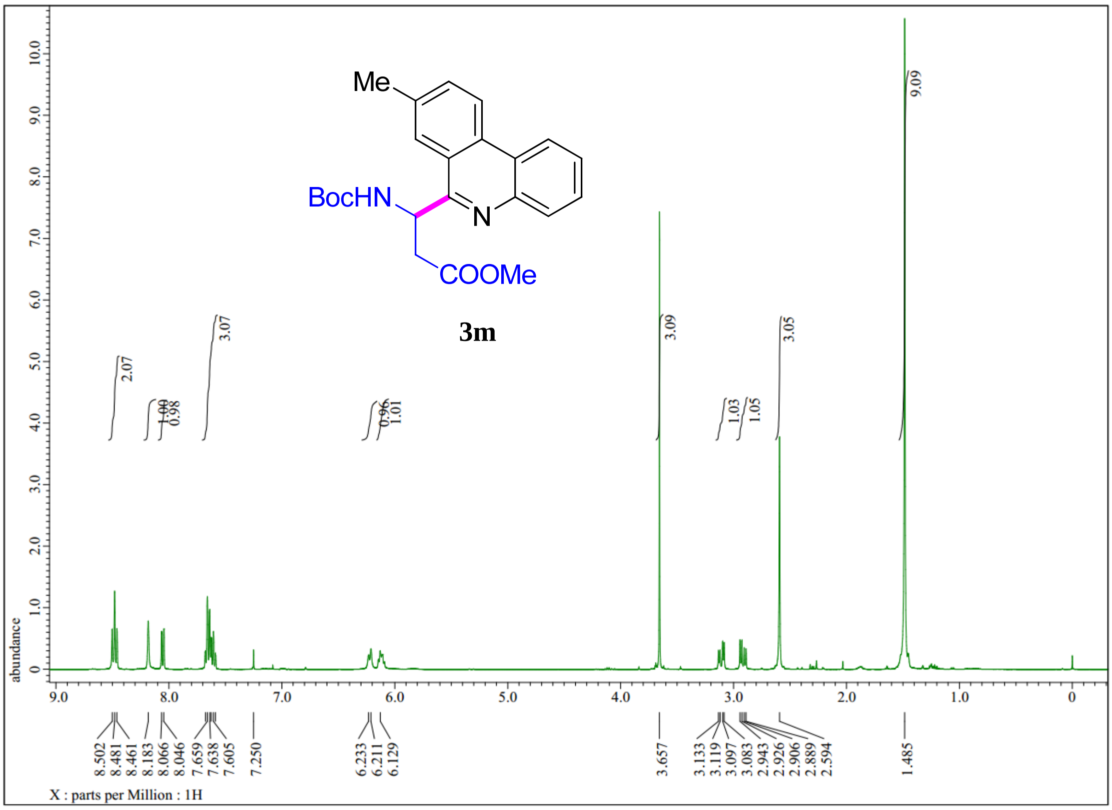

Me
BocHN
N
COOMe
3m
X : parts per Million : 1H
abundance


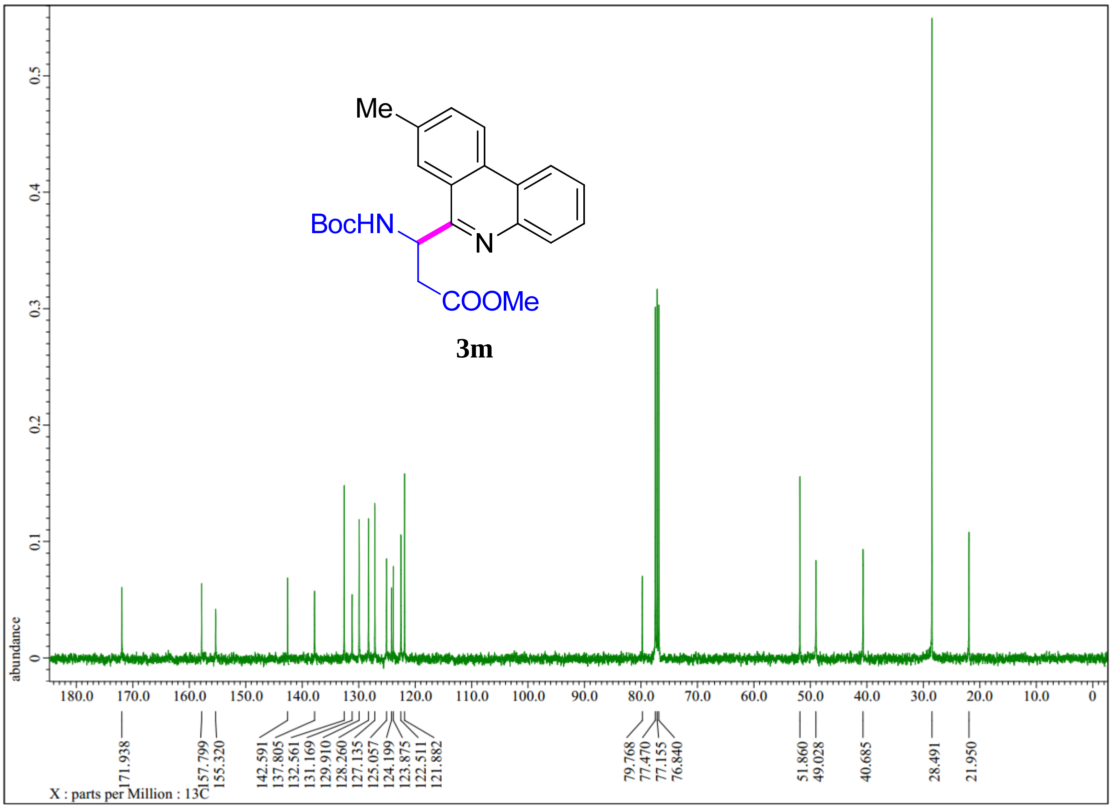

Me
BocHN
N
COOMe
3m
X : parts per Million : 13C
abundance

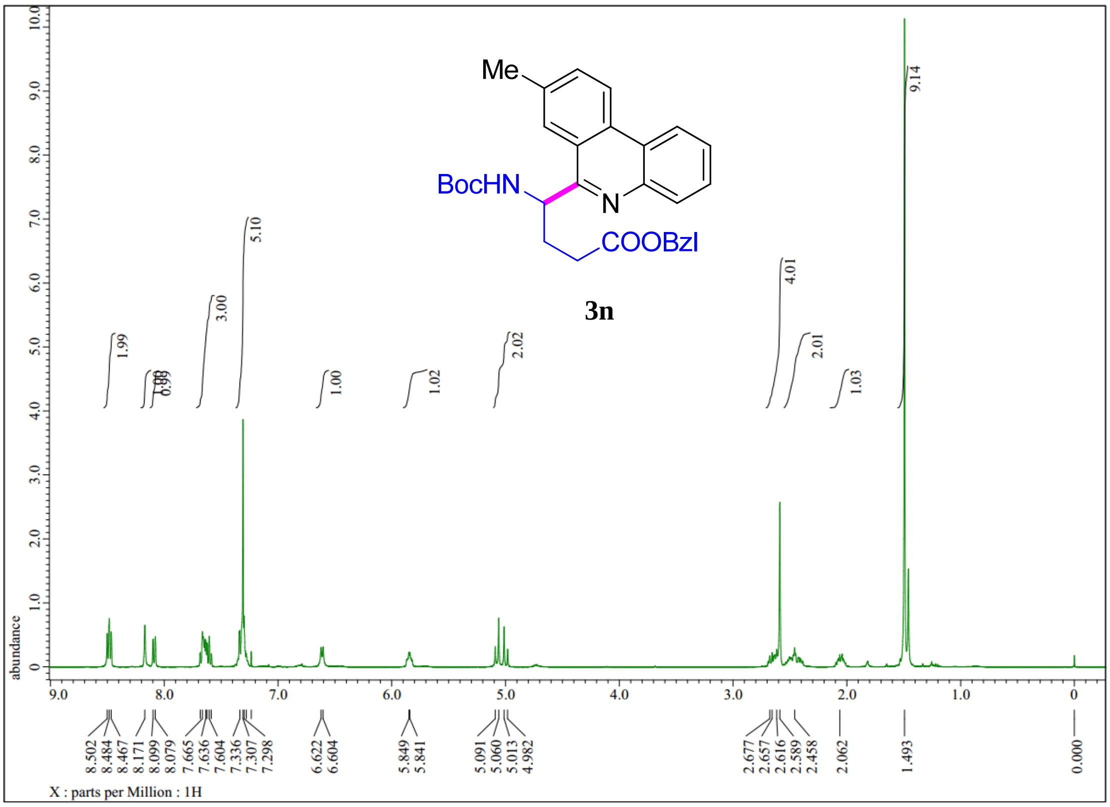
Me
BocHN
N
COOBzl
3n
X : parts per Million : 1H
abundance

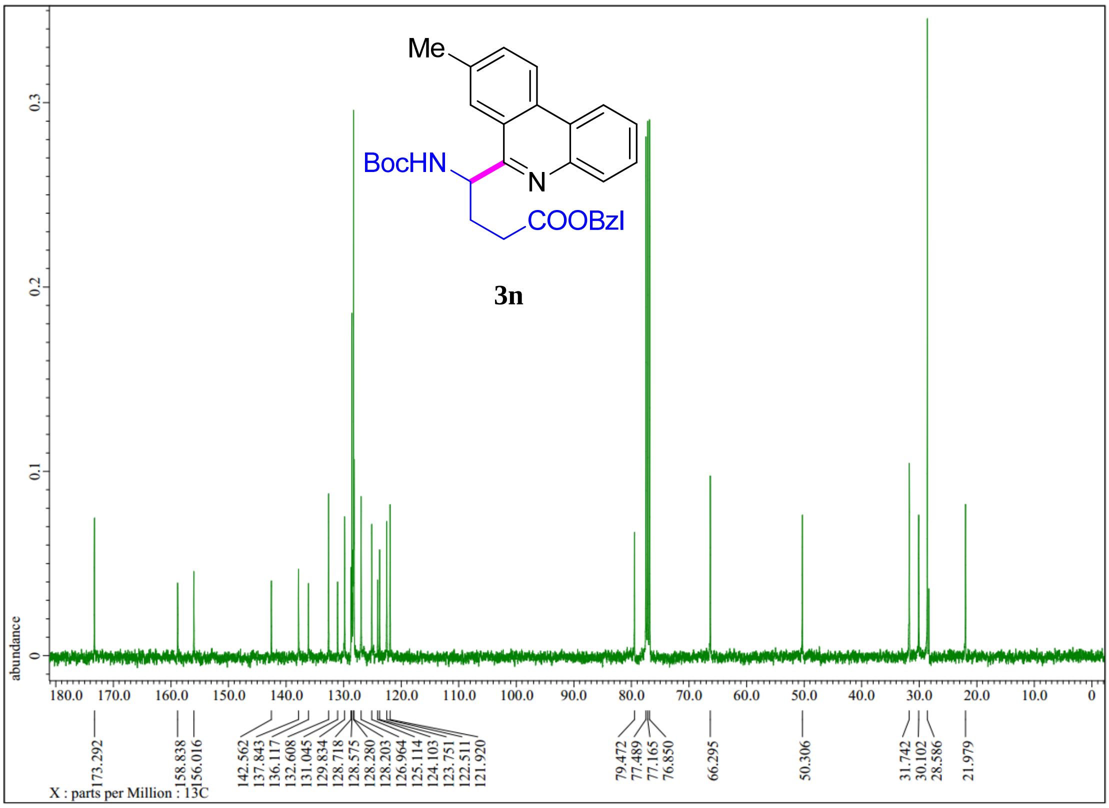
Me
BocHN
N
COOBzl
3n
X : parts per Million : 13C
abundance

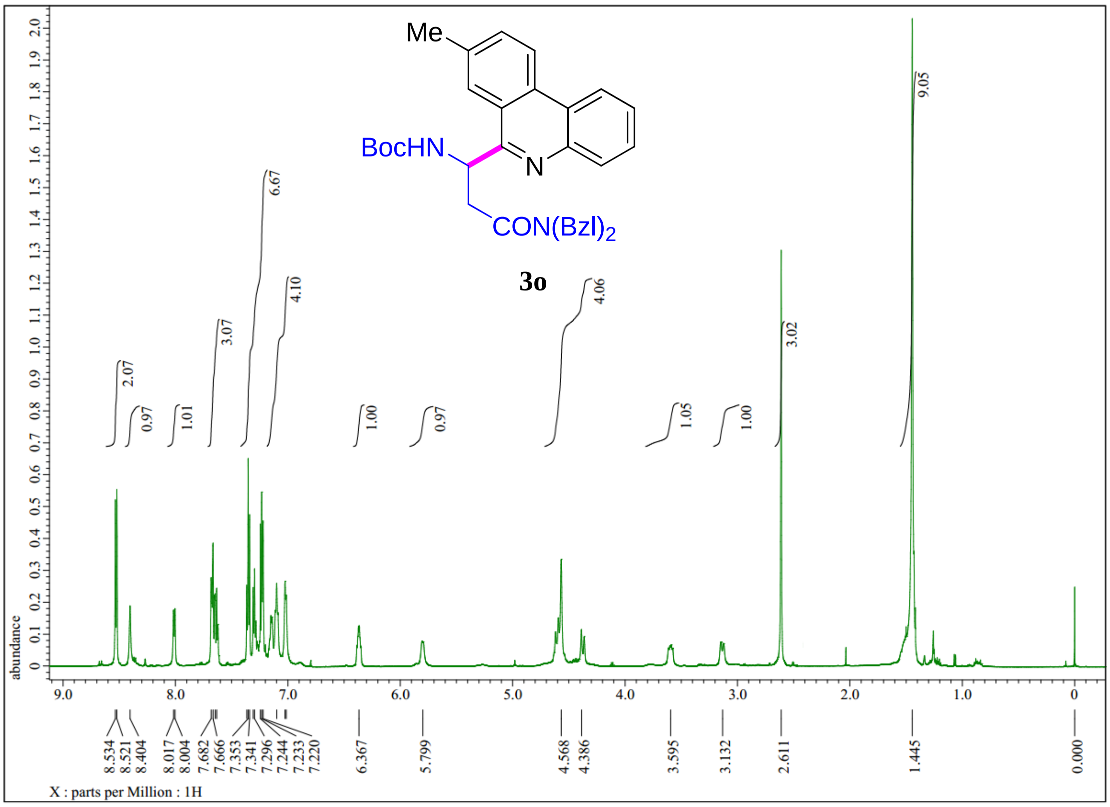

Me
BocHN
N
CON(Bzl)2
3o
X : parts per Million : 1H
abundance


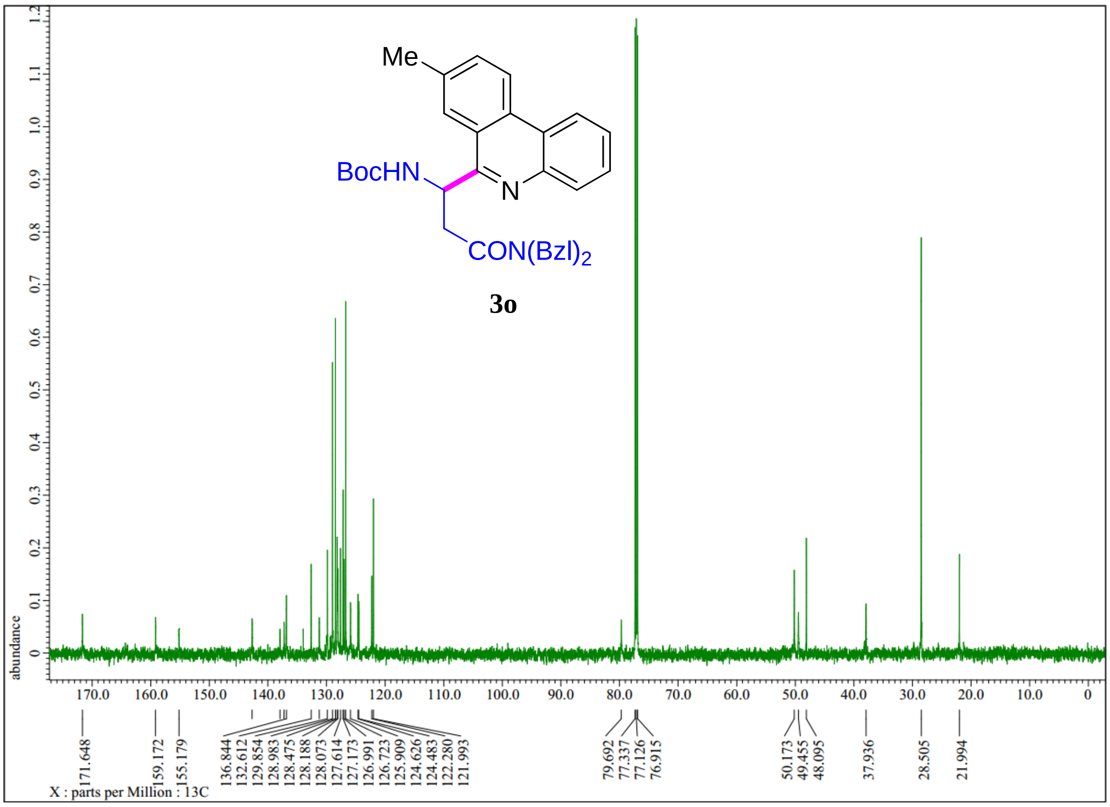

Me
BocHN
N
CON(Bzl)2
3o
X : parts per Million : 13C
abundance

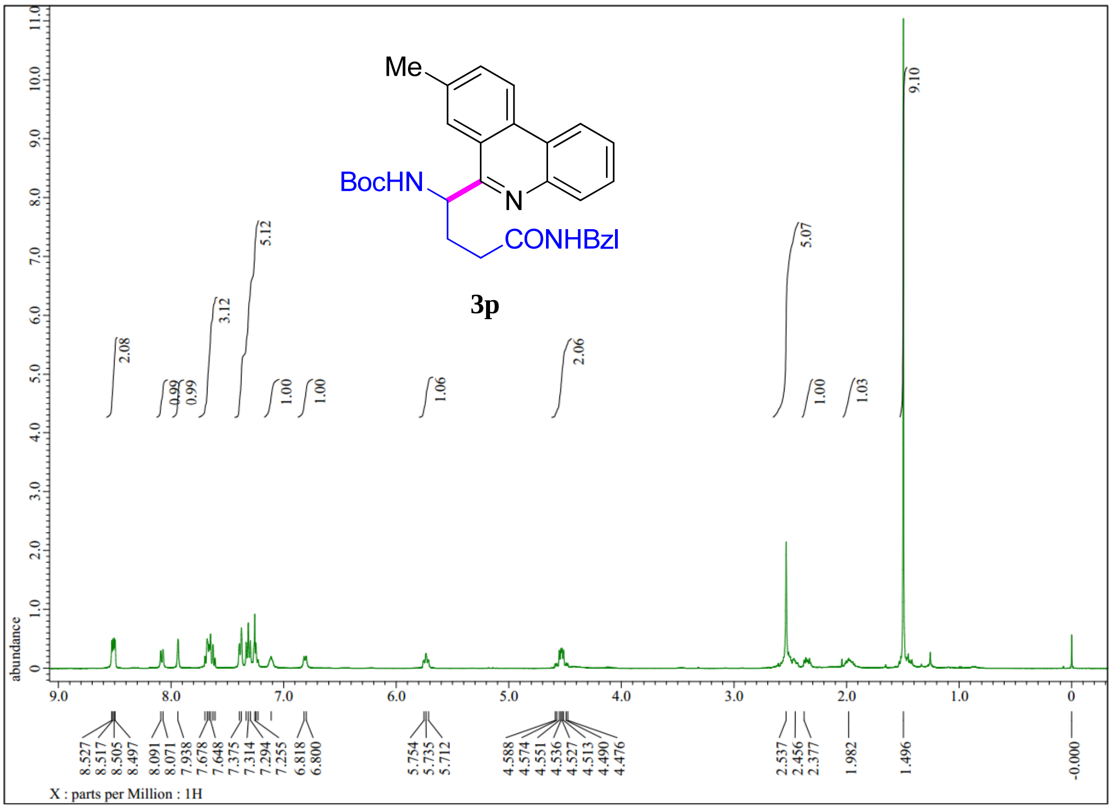

Me
BocHN
N
CONHBzl
3p
X : parts per Million : 1H
abundance


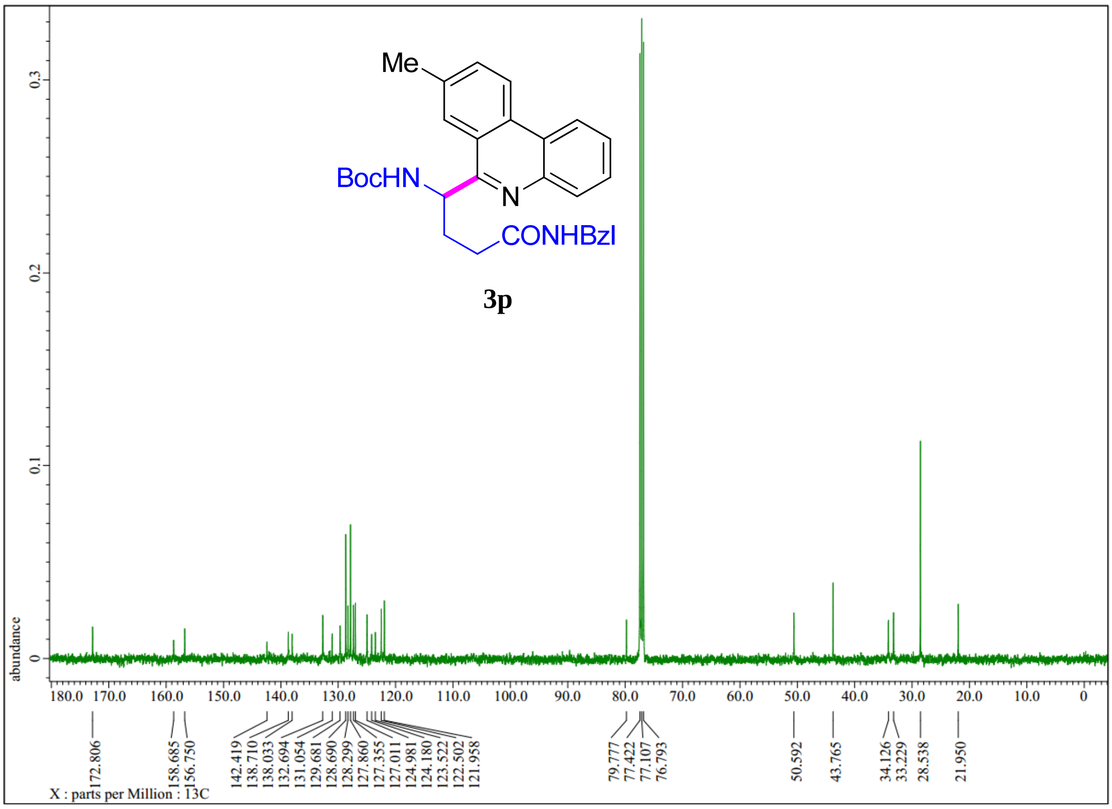

Me
BocHN
N
CONHBzl
3p
X : parts per Million : 13C
abundance

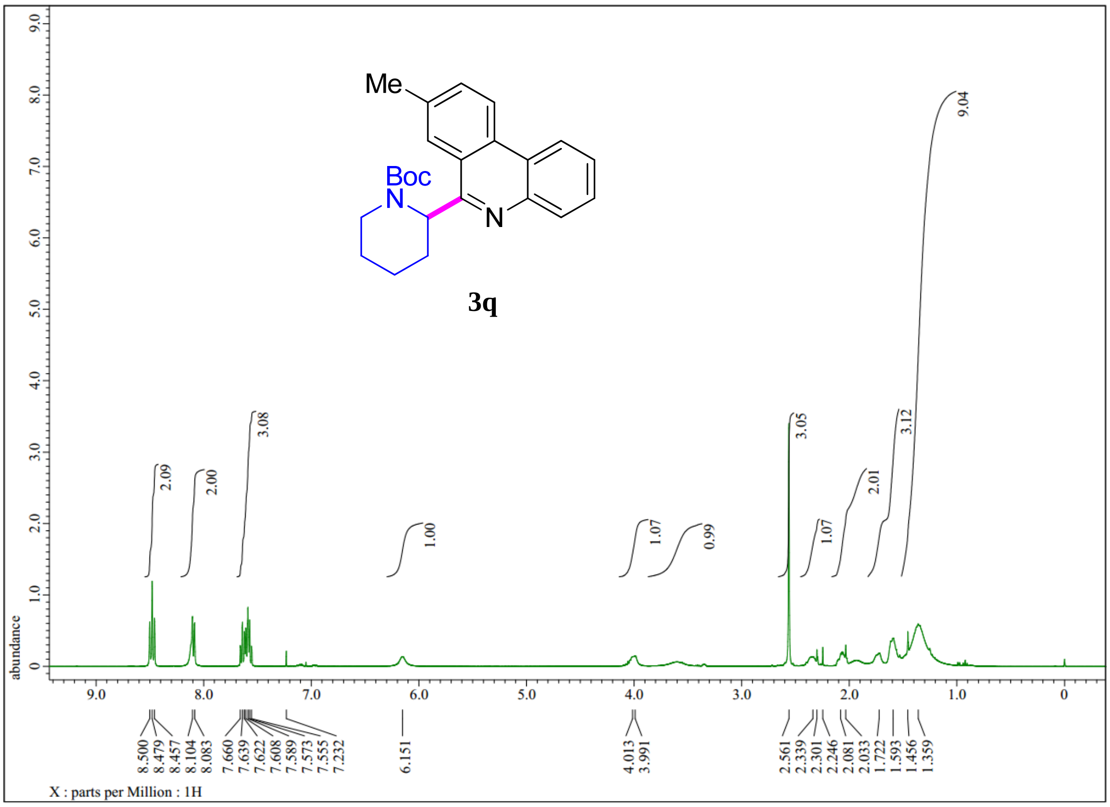

Me
Boc
N
N
3q
X : parts per Million : 1H
abundance


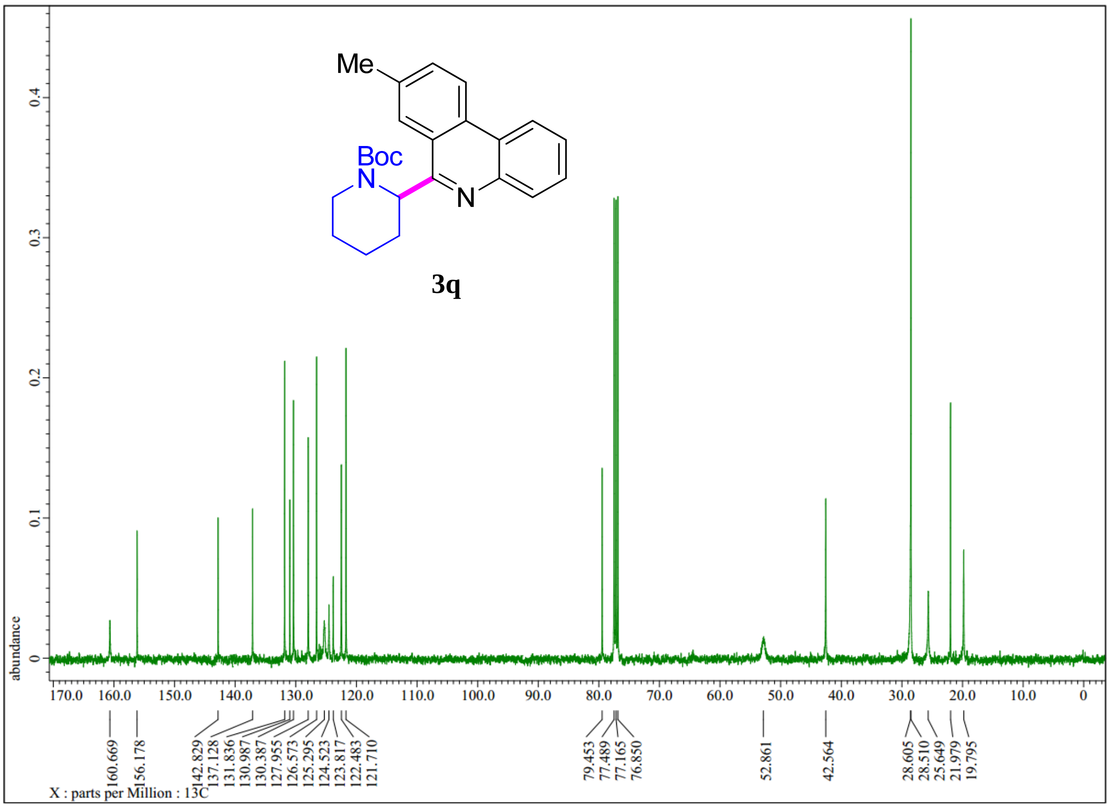

Me
Boc
N
N
3q
X : parts per Million : 13C
abundance

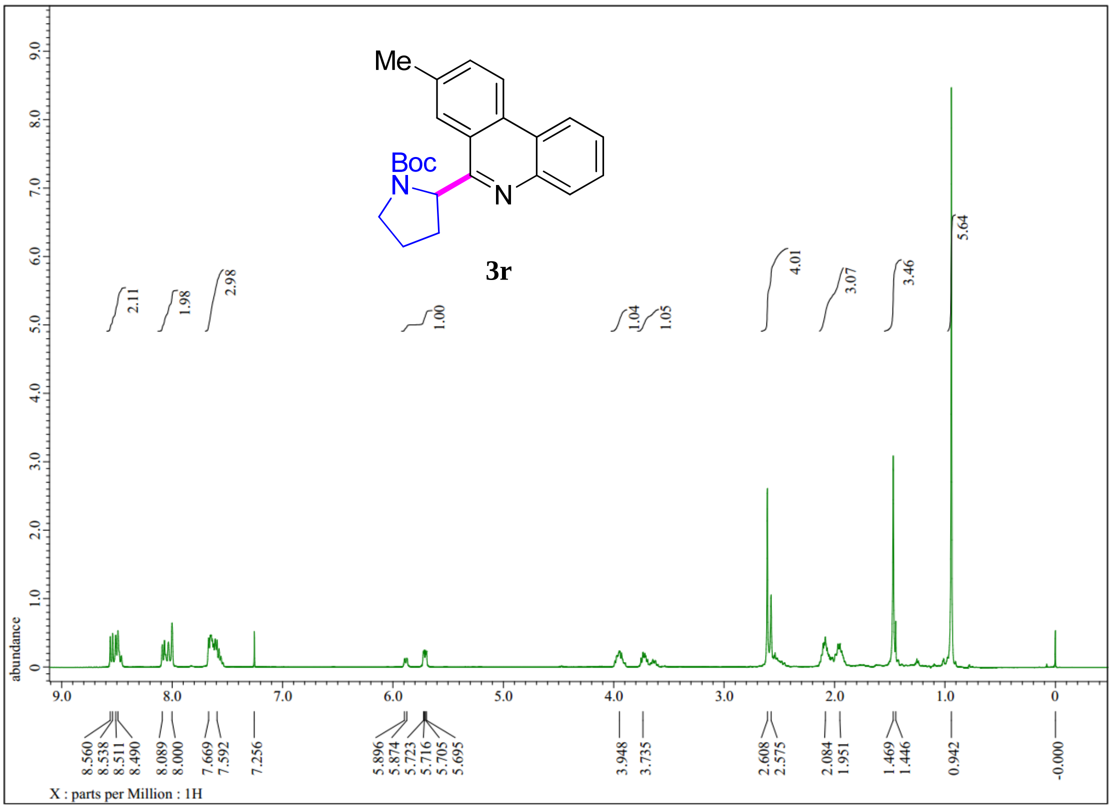
Me
Boc
N
N
3r
abundance
X : parts per Million : 1H

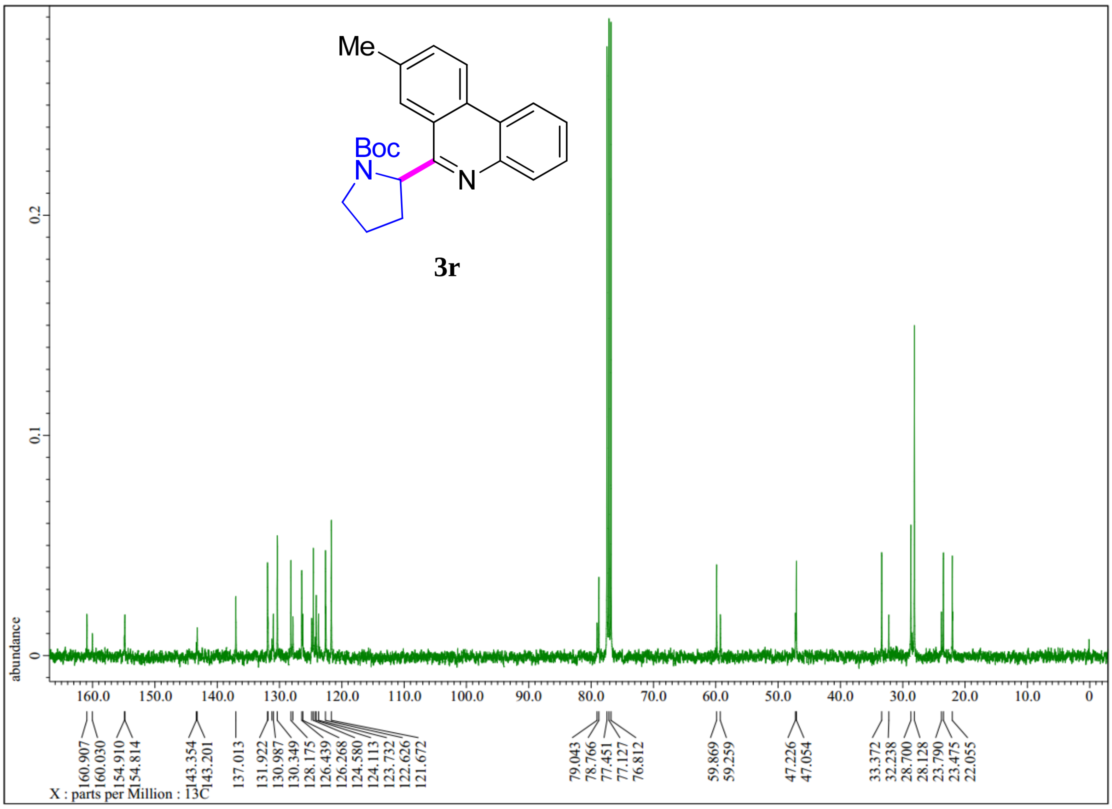
Me
Boc
N
N
3r
abundance
X : parts per Million : 13C

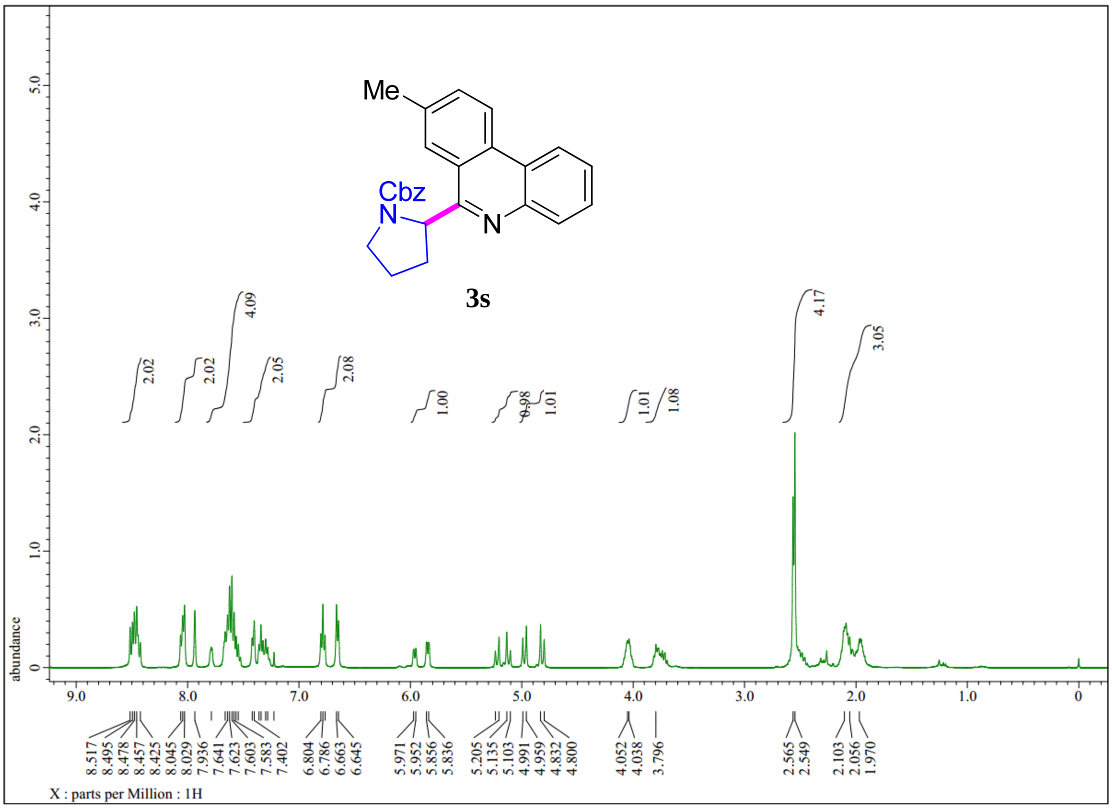
Me
Cbz
N
N
3s
X : parts per Million : 1H
abundance

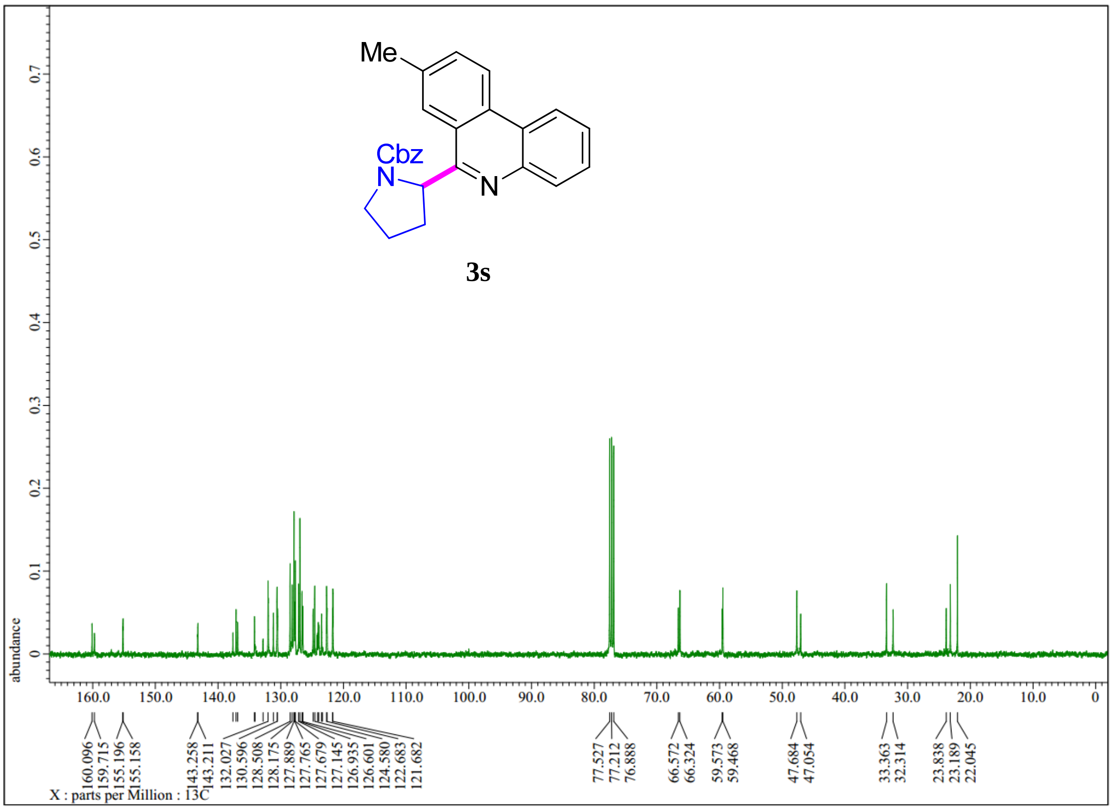
Me
Cbz
N
N
3s
X : parts per Million : 13C
abundance

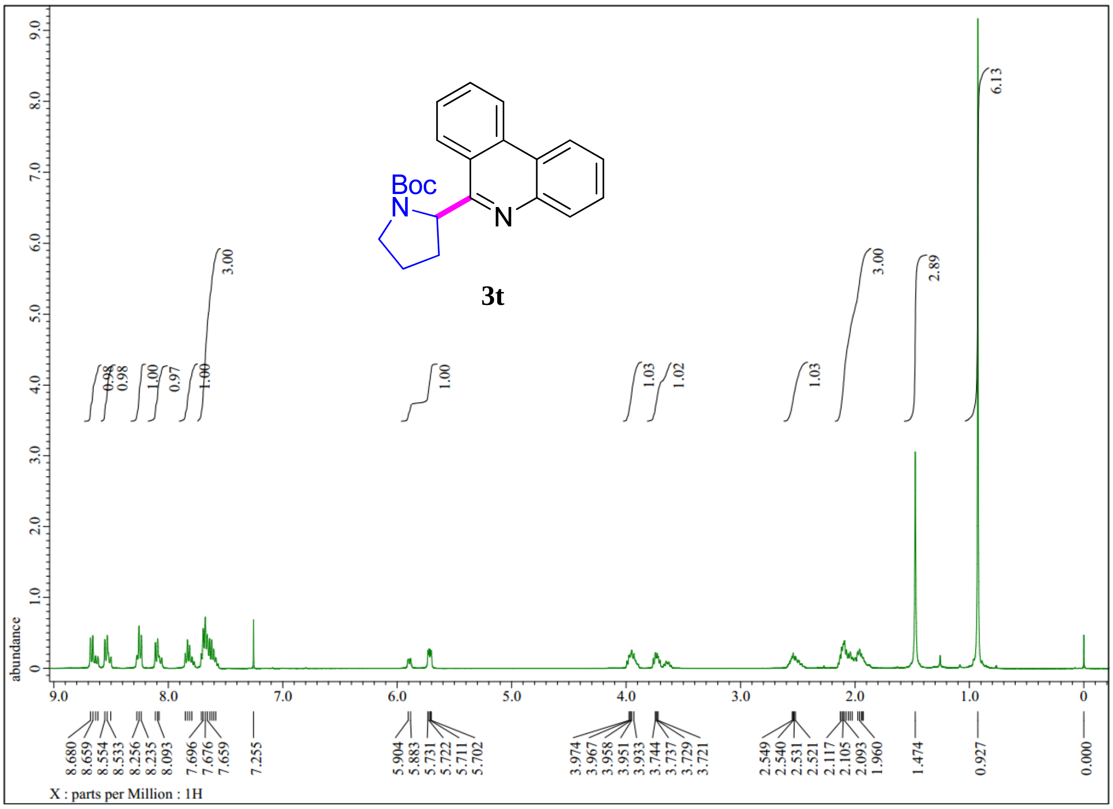

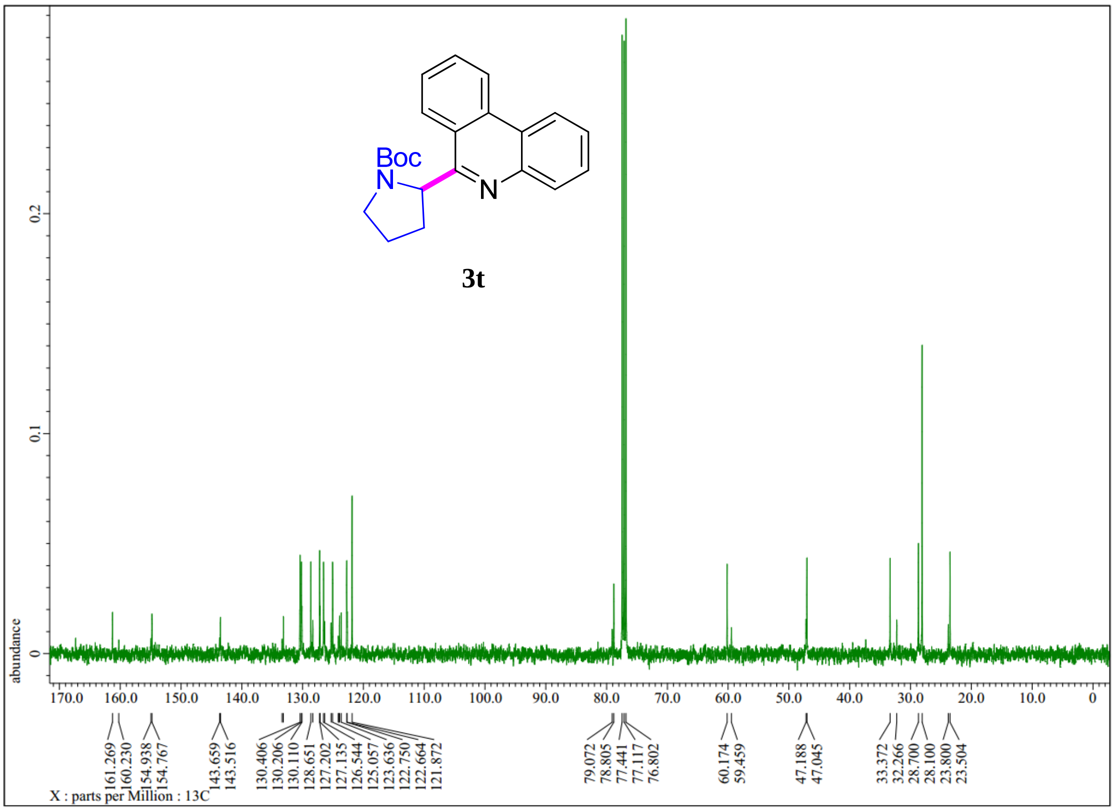

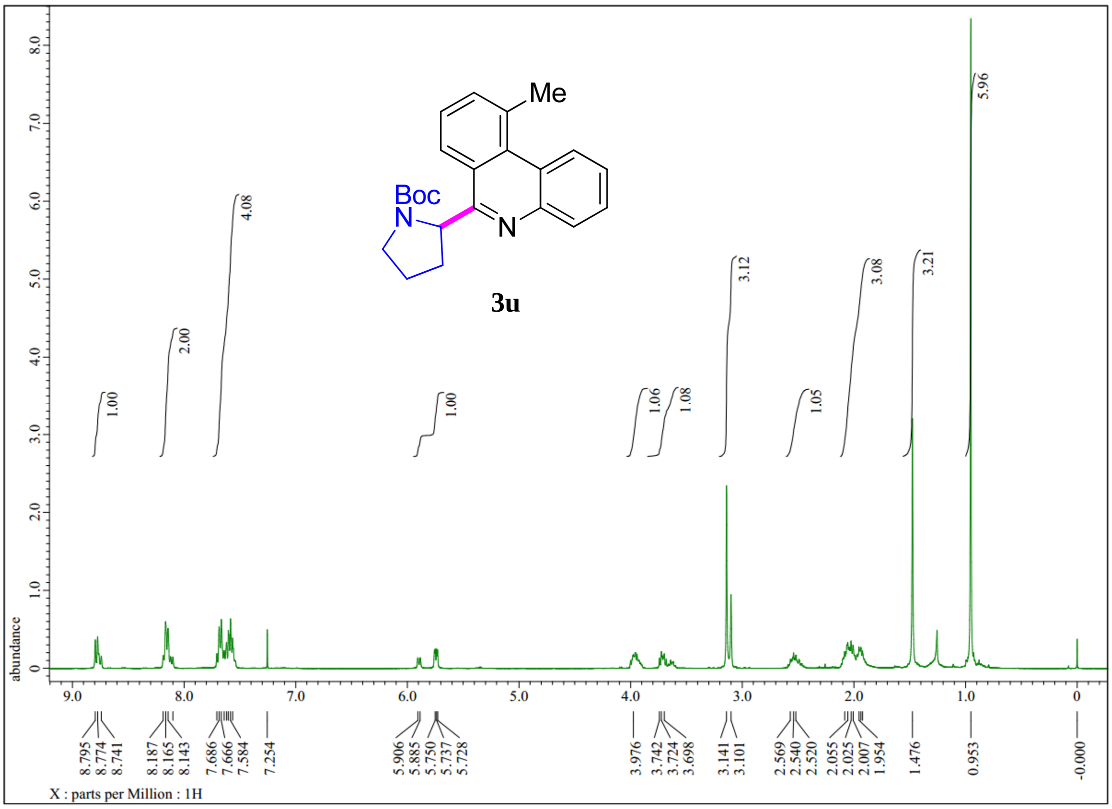

Me
Boc
N
N
3u
X : parts per Million : 1H
abundance


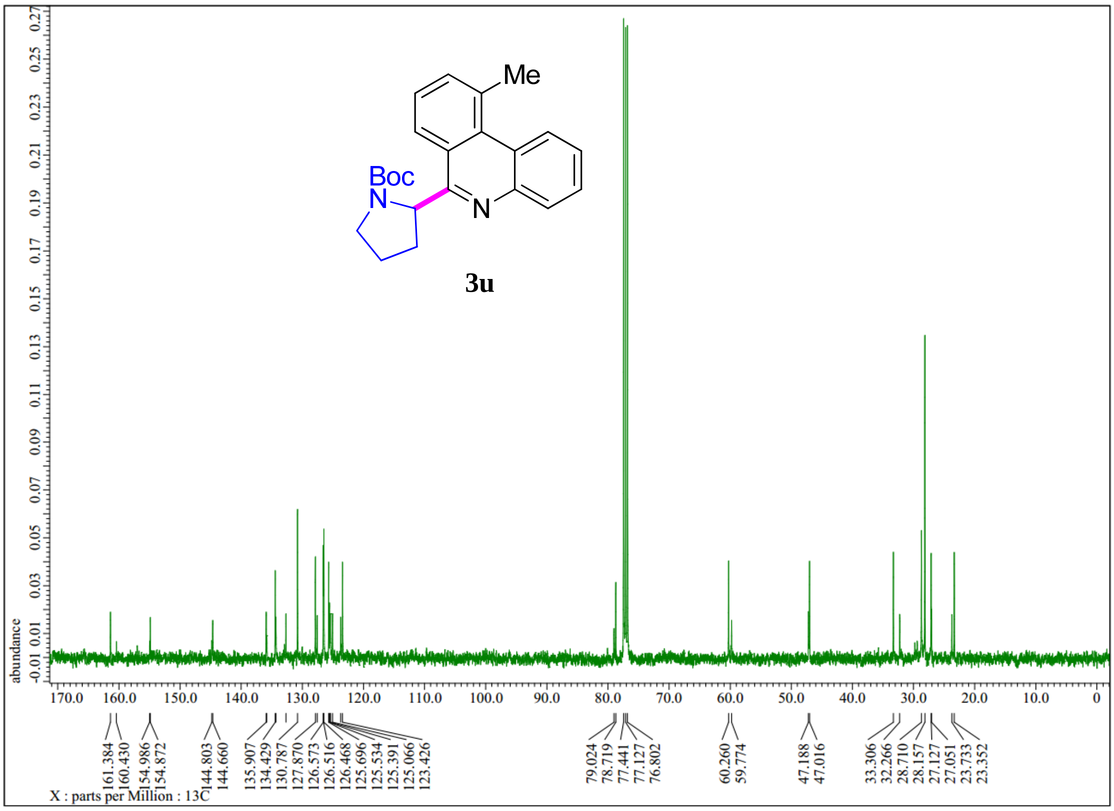

Me
Boc
N
N
3u
X : parts per Million : 13C
abundance

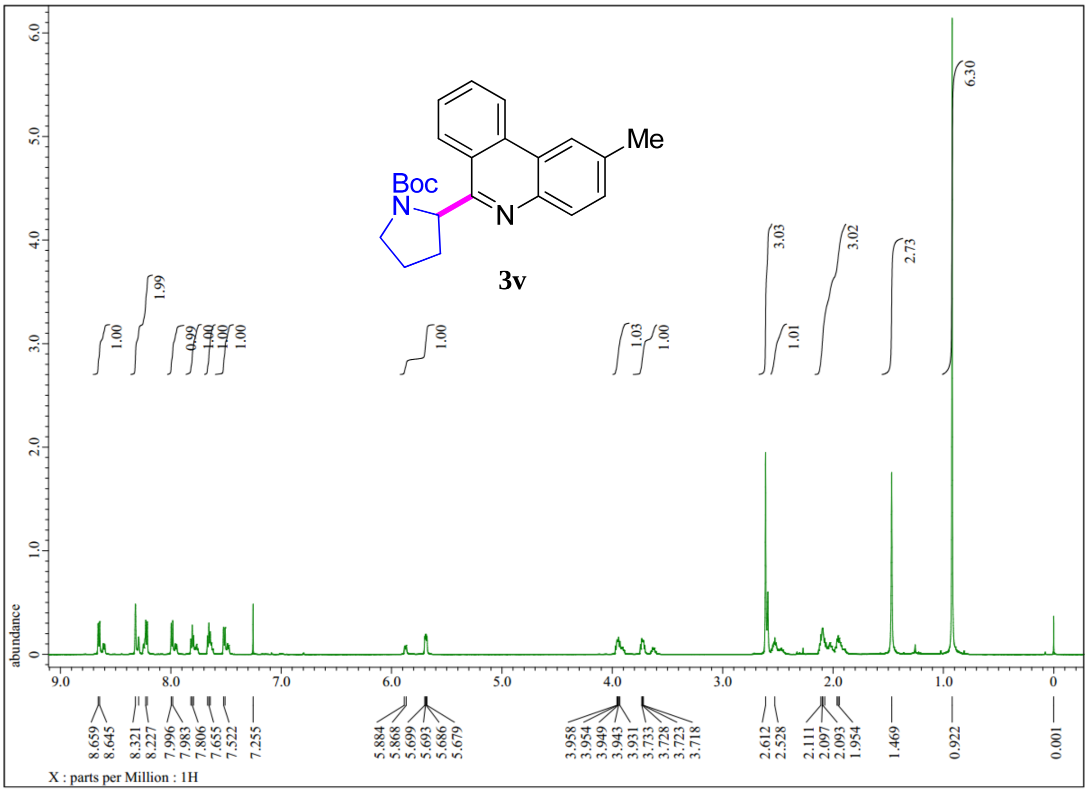
Boc
N
N
Me
3v
abundance
X : parts per Million : 1H
8.659
8.645
8.321
8.227
7.996
7.983
7.806
7.655
7.522
7.255
5.884
5.868
5.699
5.693
5.686
5.679
3.958
3.954
3.949
3.943
3.931
3.733
3.728
3.723
3.718
2.612
2.528
2.111
2.097
2.093
1.954
1.469
0.922
0.001

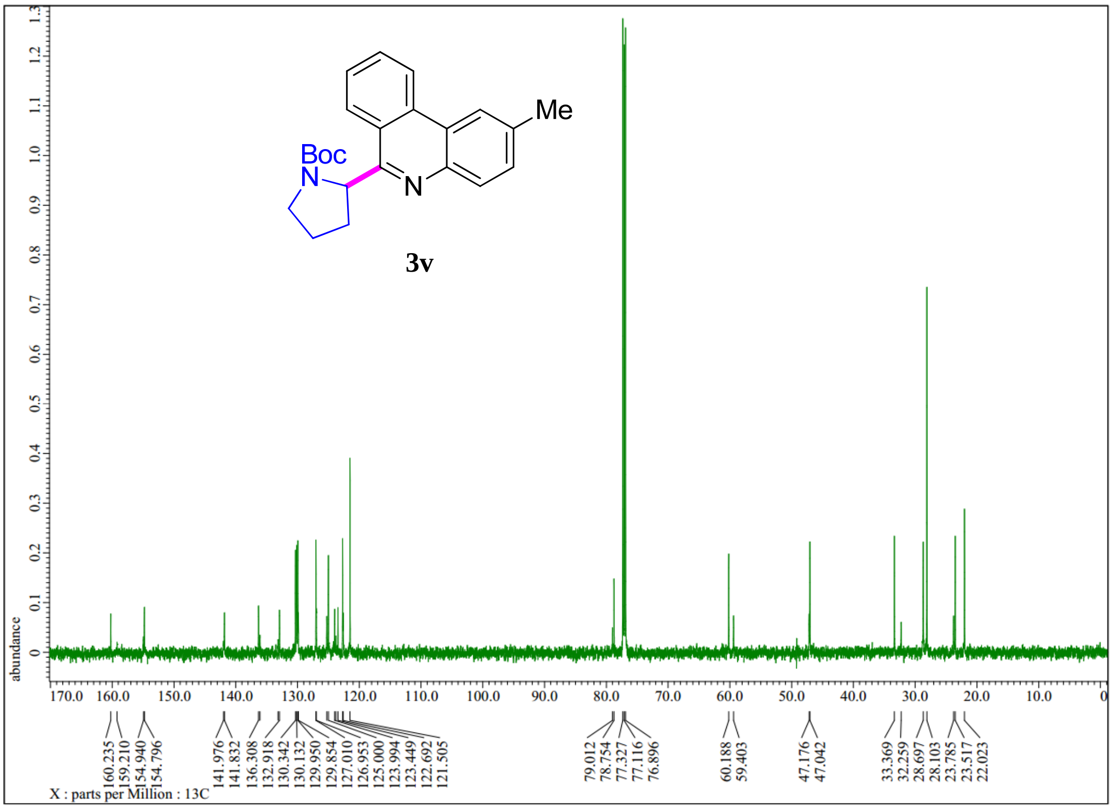
Boc
N
N
Me
3v
abundance
X : parts per Million : 13C
160.235
159.210
154.940
154.796
141.976
141.832
136.308
132.918
130.342
130.132
129.950
129.854
127.010
126.953
125.000
123.994
123.449
122.692
121.505
79.012
78.754
77.327
77.116
76.896
60.188
59.403
47.176
47.042
33.369
32.259
28.697
28.103
23.785
23.517
22.023

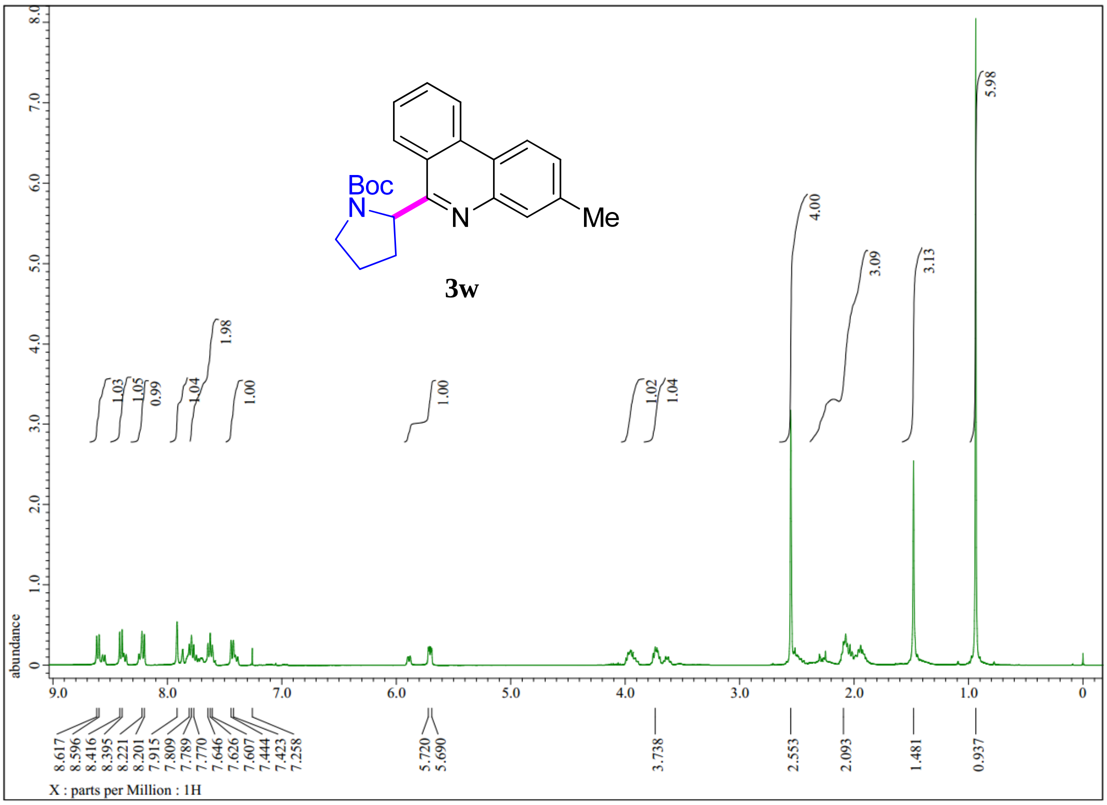
Boc
N
N
Me
3w
abundance
X : parts per Million : 1H

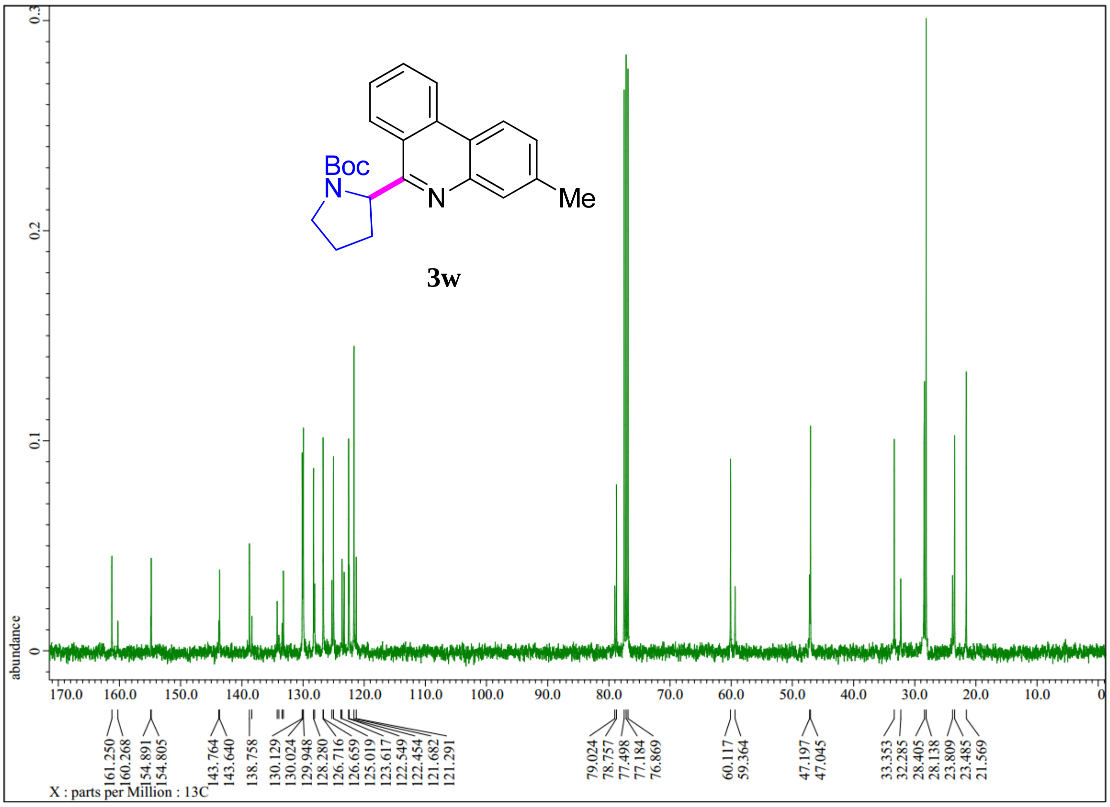
Boc
N
N
Me
3w
abundance
X : parts per Million : 13C

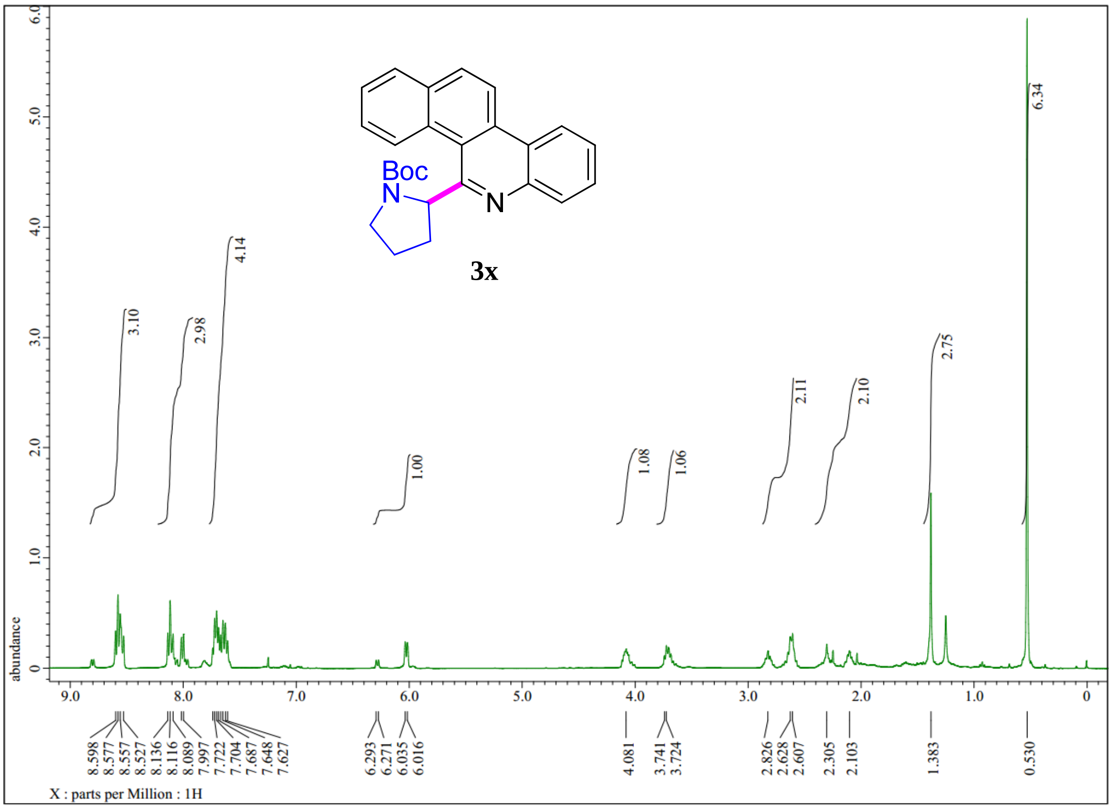
3x
Boc
N
abundance
X : parts per Million : 1H

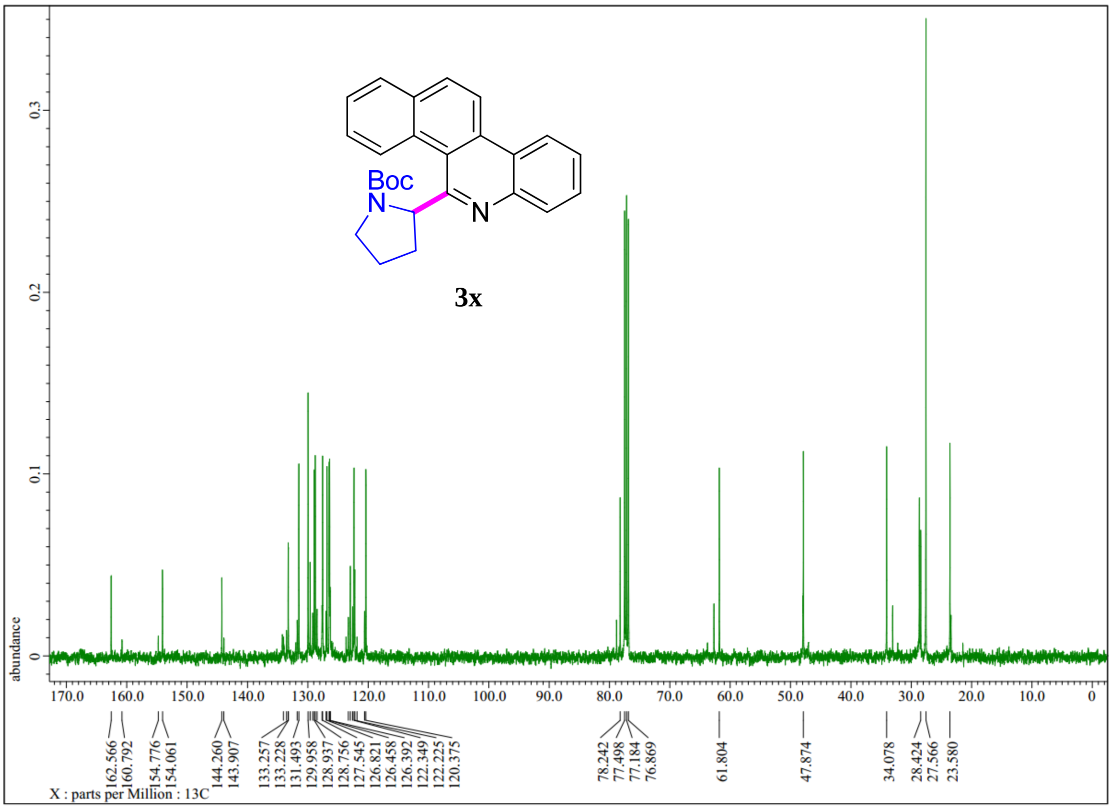
3x
Boc
N
abundance
X : parts per Million : 13C

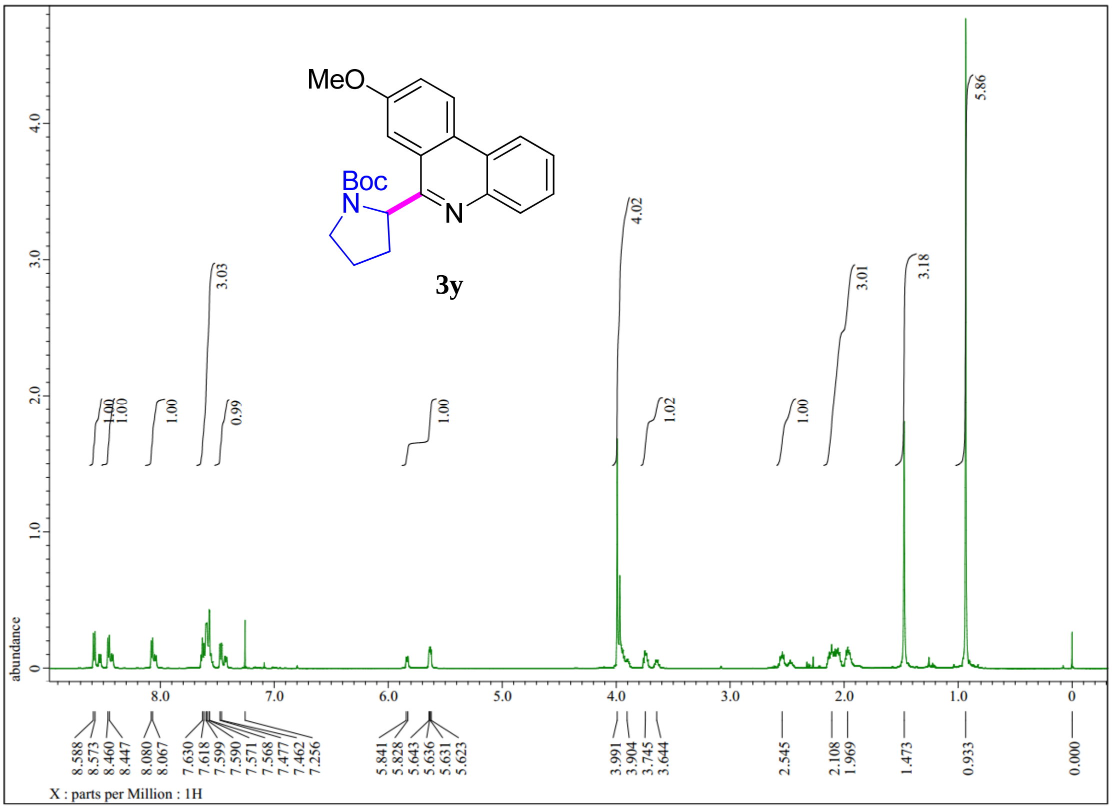
MeO
Boc
N
N
3y
X : parts per Million : 1H
abundance

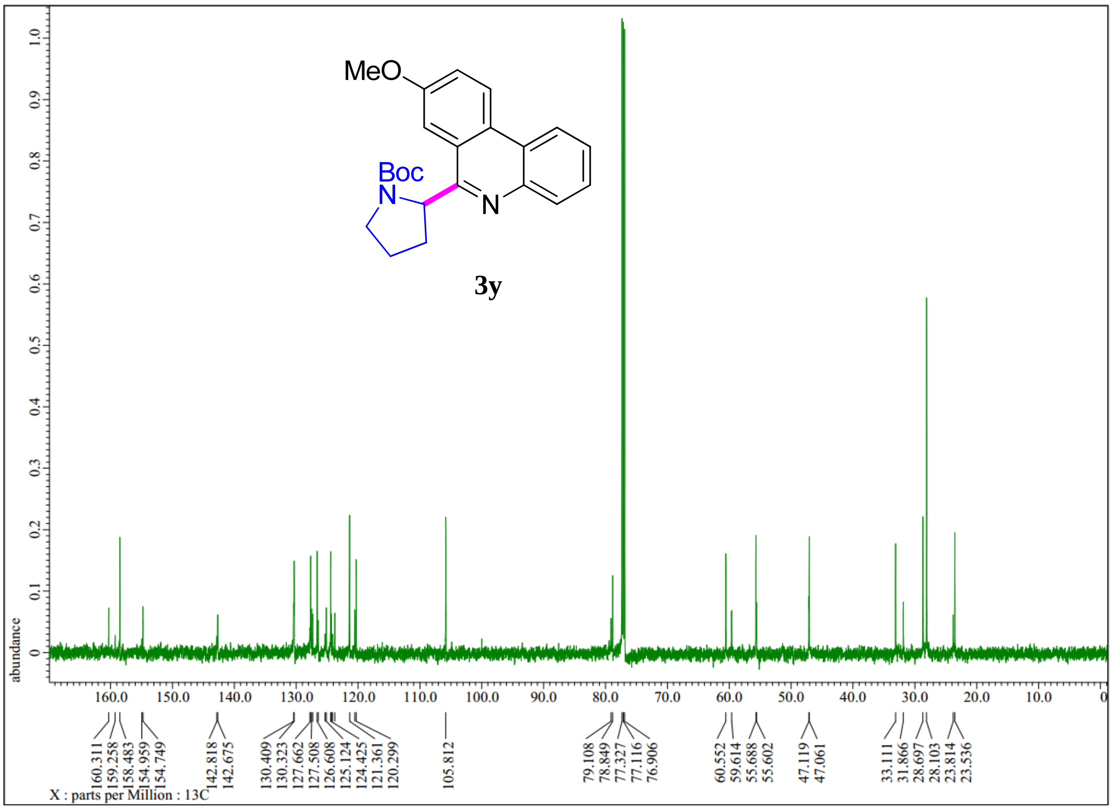
MeO
Boc
N
N
3y
X : parts per Million : 13C
abundance

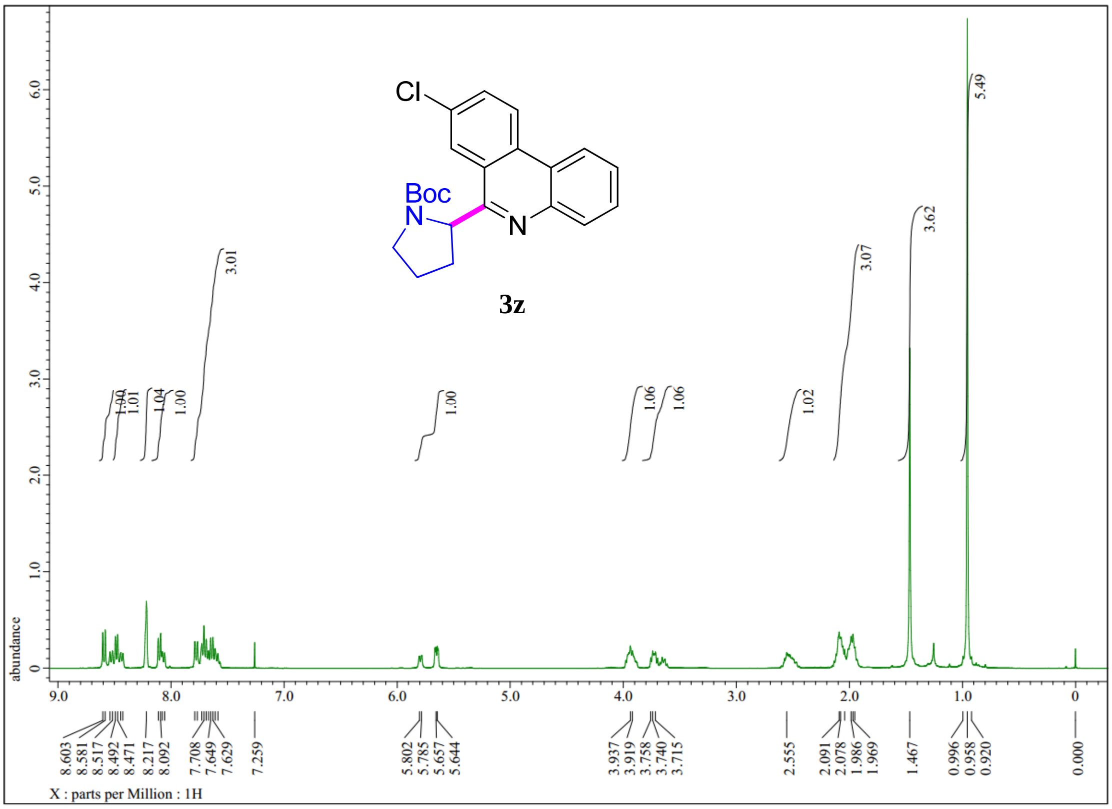
Cl
Boc
N
N
3z
X : parts per Million : 1H
abundance

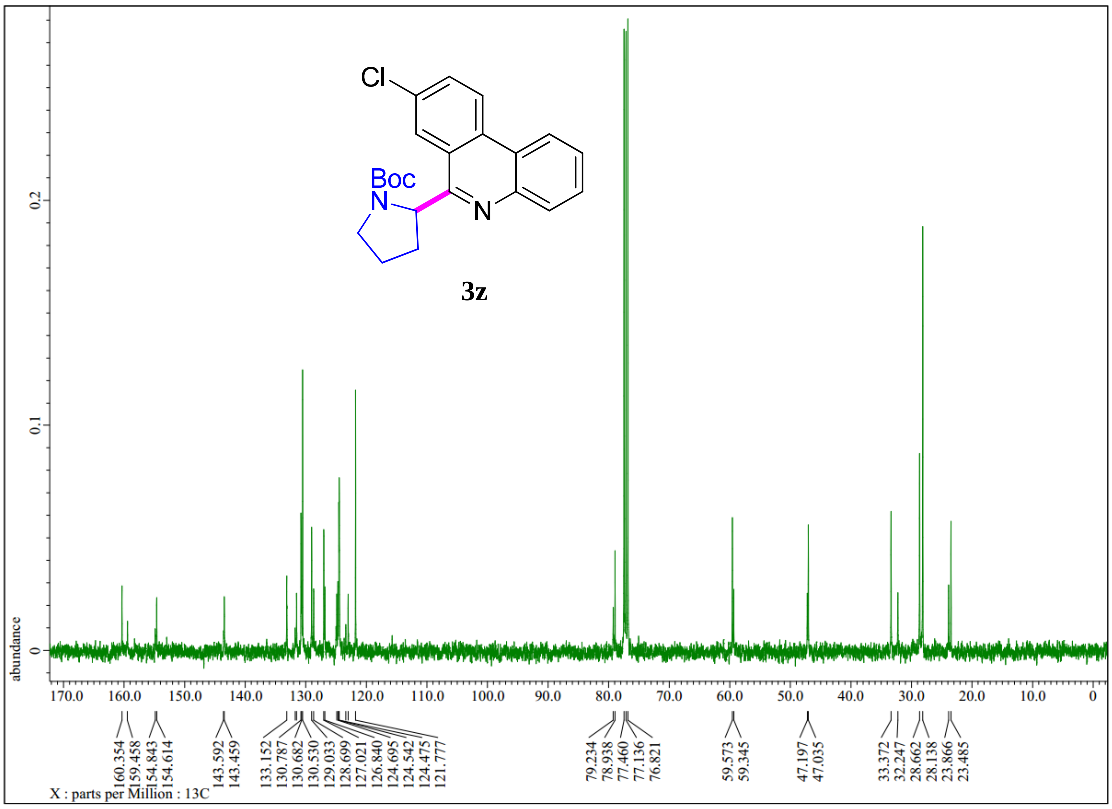
Cl
Boc
N
N
3z
X : parts per Million : 13C
abundance

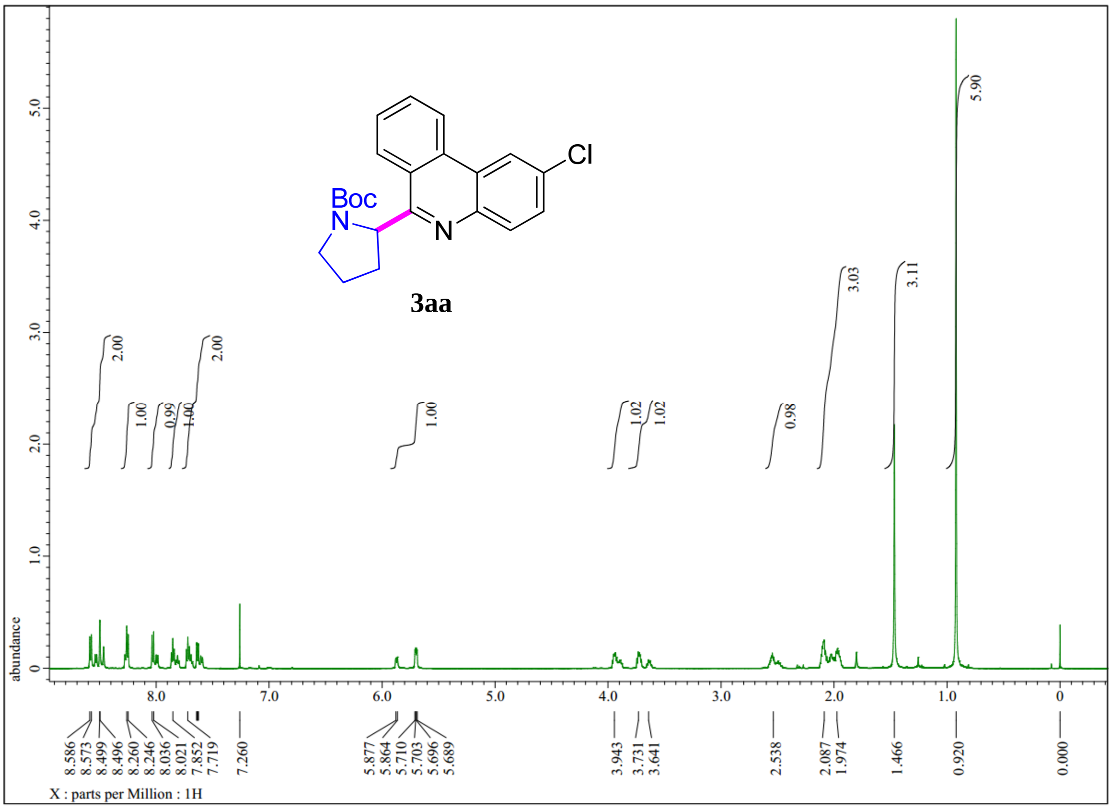
Boc
N
N
Cl
3aa
X : parts per Million : 1H
abundance

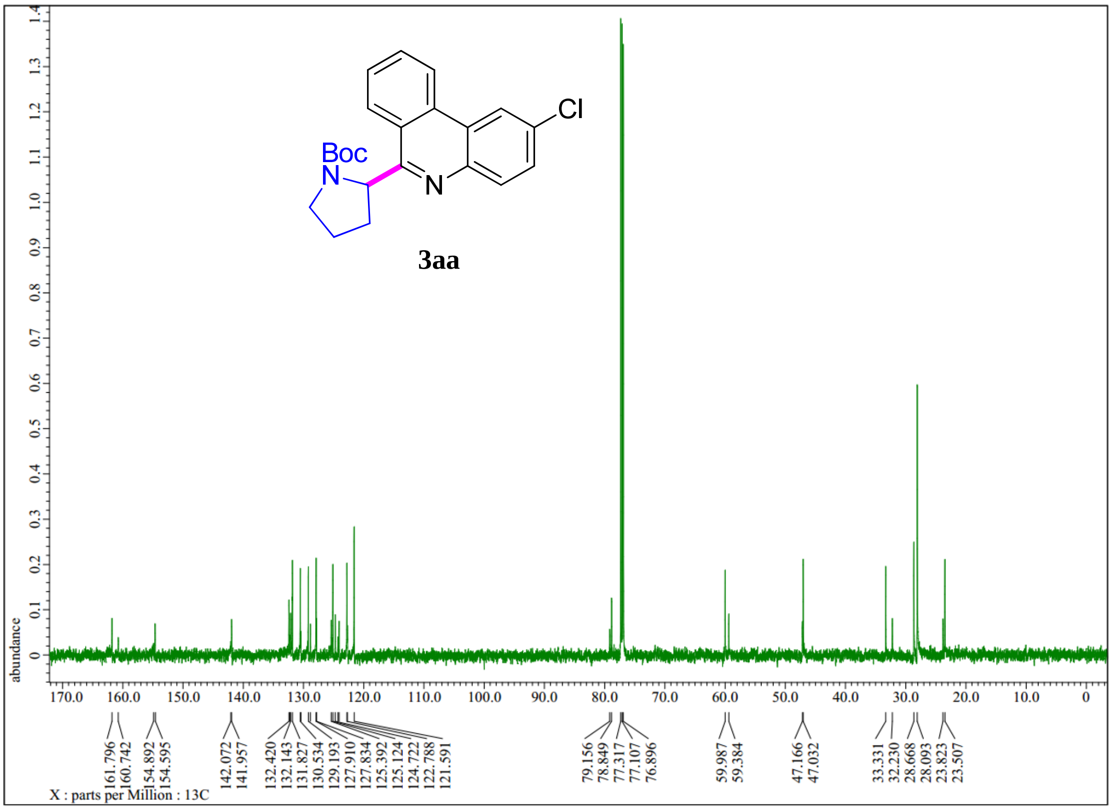
Boc
N
N
Cl
3aa
X : parts per Million : 13C
abundance

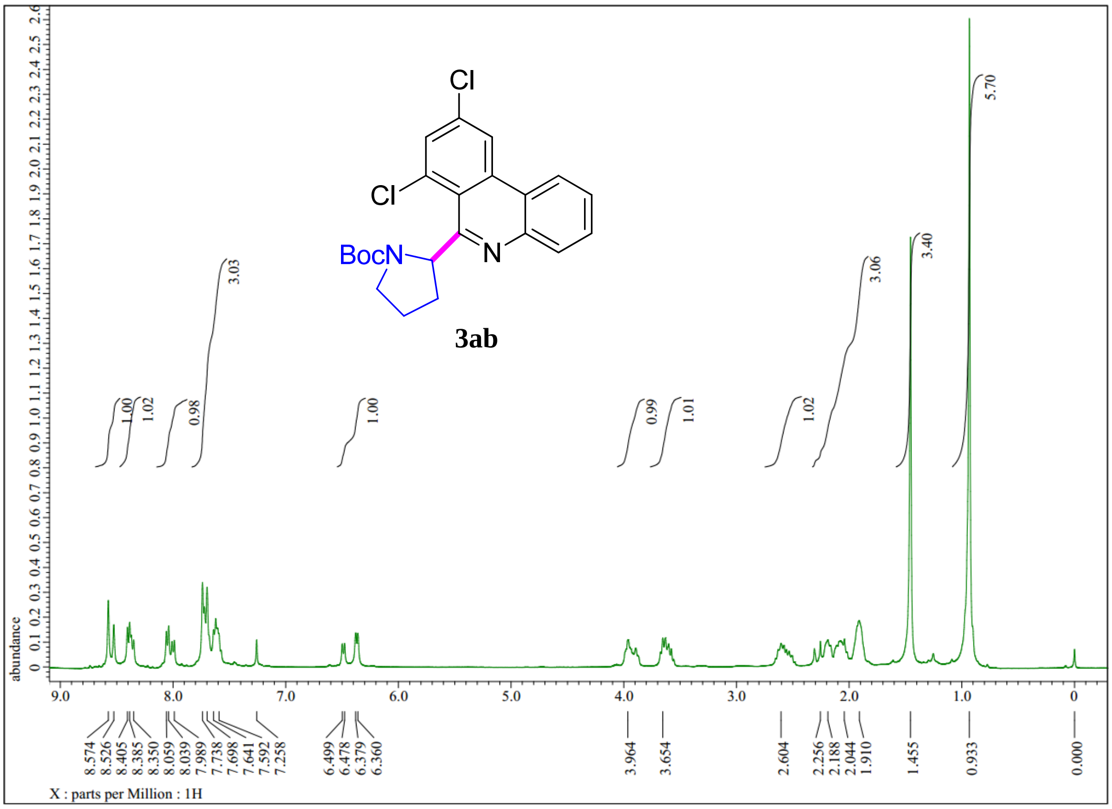
Cl
Cl
BocN
N
3ab
abundance
X : parts per Million : 1H
1.00 1.02 0.98 3.03 1.00 0.99 1.01 1.02 3.06 3.40 5.70
8.574 8.526 8.405 8.385 8.350 8.059 8.039 7.989 7.738 7.698 7.641 7.592 7.258
6.499 6.478 6.379 6.360
3.964 3.654 2.604 2.256 2.188 2.044 1.910 1.455 0.933 0.000

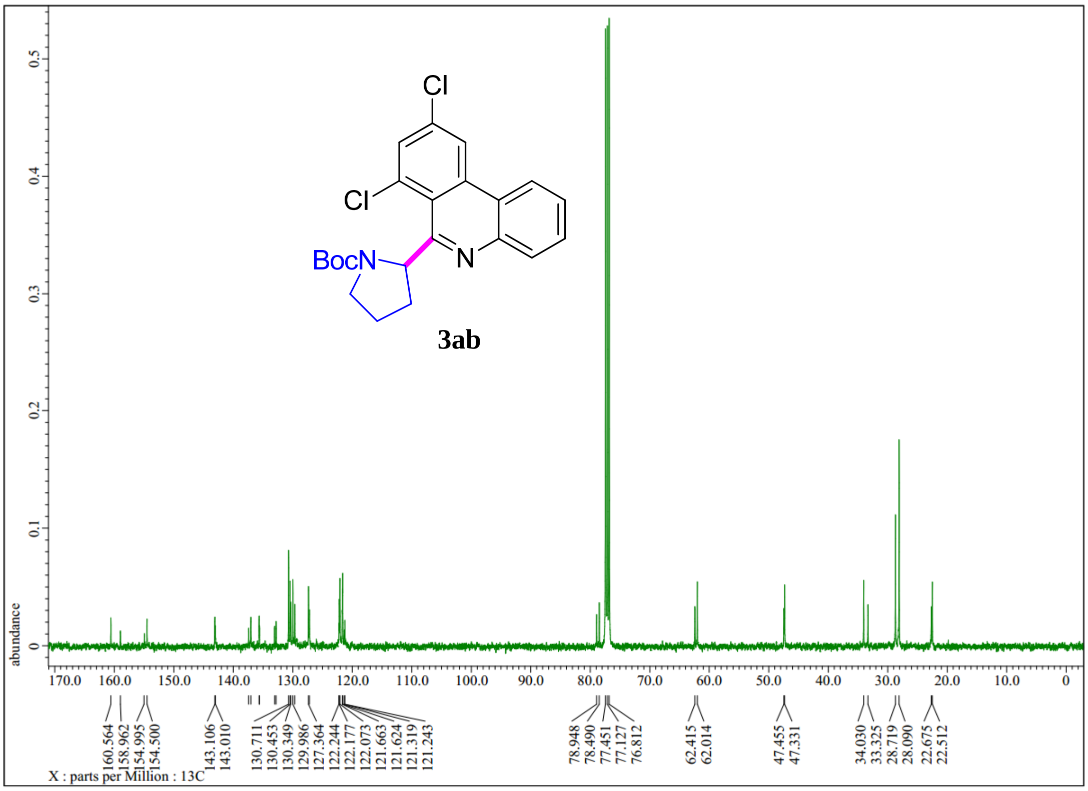
Cl
Cl
BocN
N
3ab
abundance
X : parts per Million : 13C
160.564 158.962 154.995 154.500 143.106 143.010
130.711 130.453 130.349 129.986 127.364 122.244 122.177 122.073 121.663 121.624 121.319 121.243
78.948 78.490 77.451 77.127 76.812 62.415 62.014 47.455 47.331
34.030 33.325 28.719 28.090 22.675 22.512

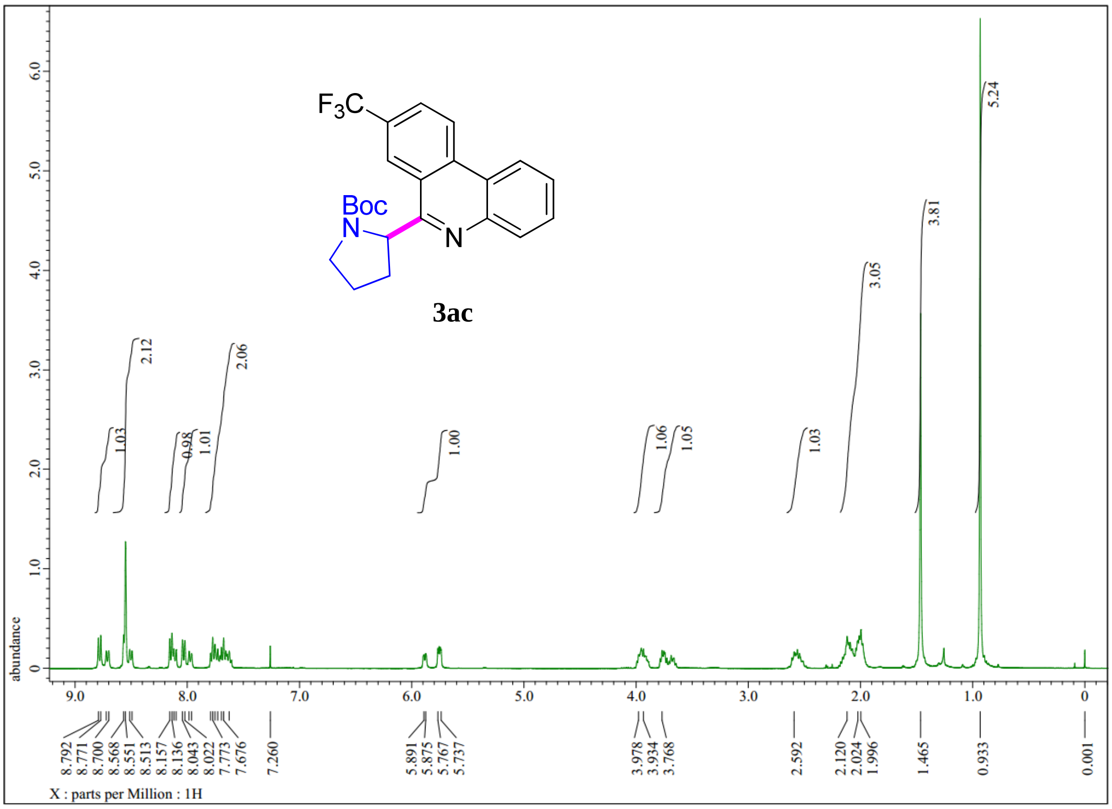

F3C
Boc
N
N
3ac
abundance
X : parts per Million : 1H
8.792
8.771
8.700
8.568
8.551
8.513
8.157
8.136
8.043
8.022
7.773
7.676
7.260
5.891
5.875
5.767
5.737
3.978
3.934
3.768
2.592
2.120
2.024
1.996
1.465
0.933
0.001


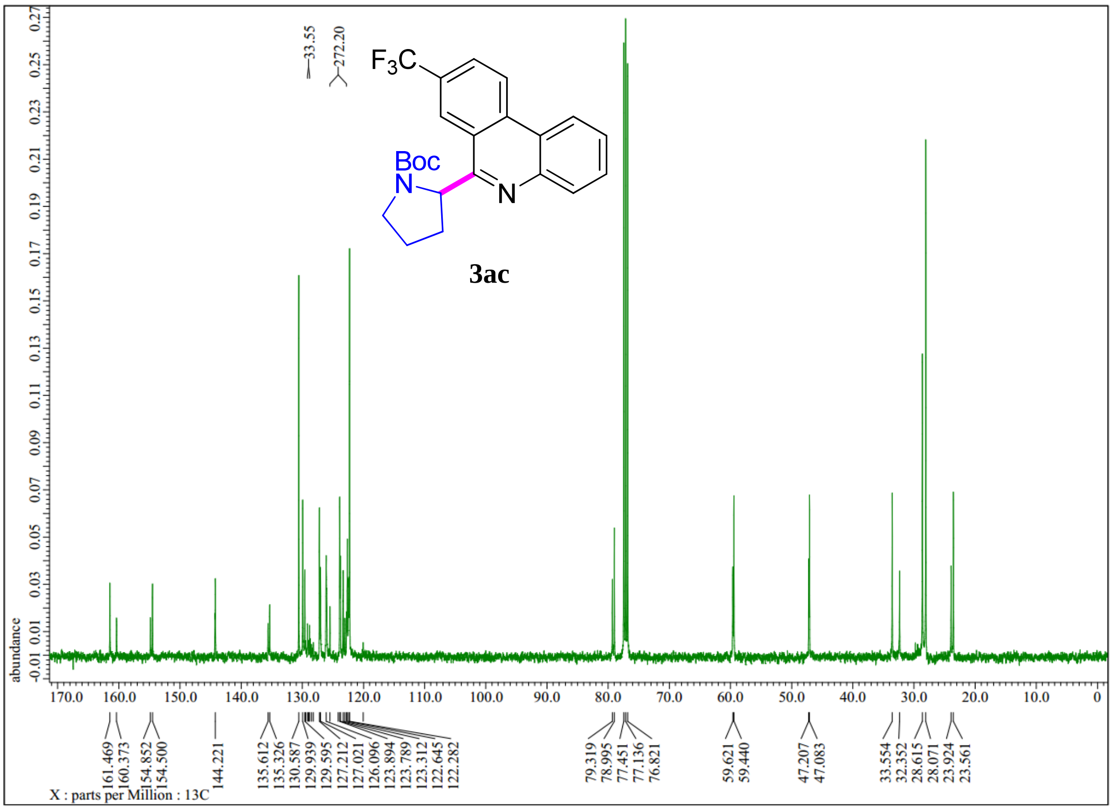

F3C
Boc
N
N
3ac
33.55
272.20
abundance
X : parts per Million : 13C
161.469
160.373
154.852
154.500
144.221
135.612
135.326
130.587
129.939
129.595
127.212
127.021
126.096
123.894
123.789
123.312
122.645
122.282
79.319
78.995
77.451
77.136
76.821
59.621
59.440
47.207
47.083
33.554
32.352
28.615
28.071
23.924
23.561

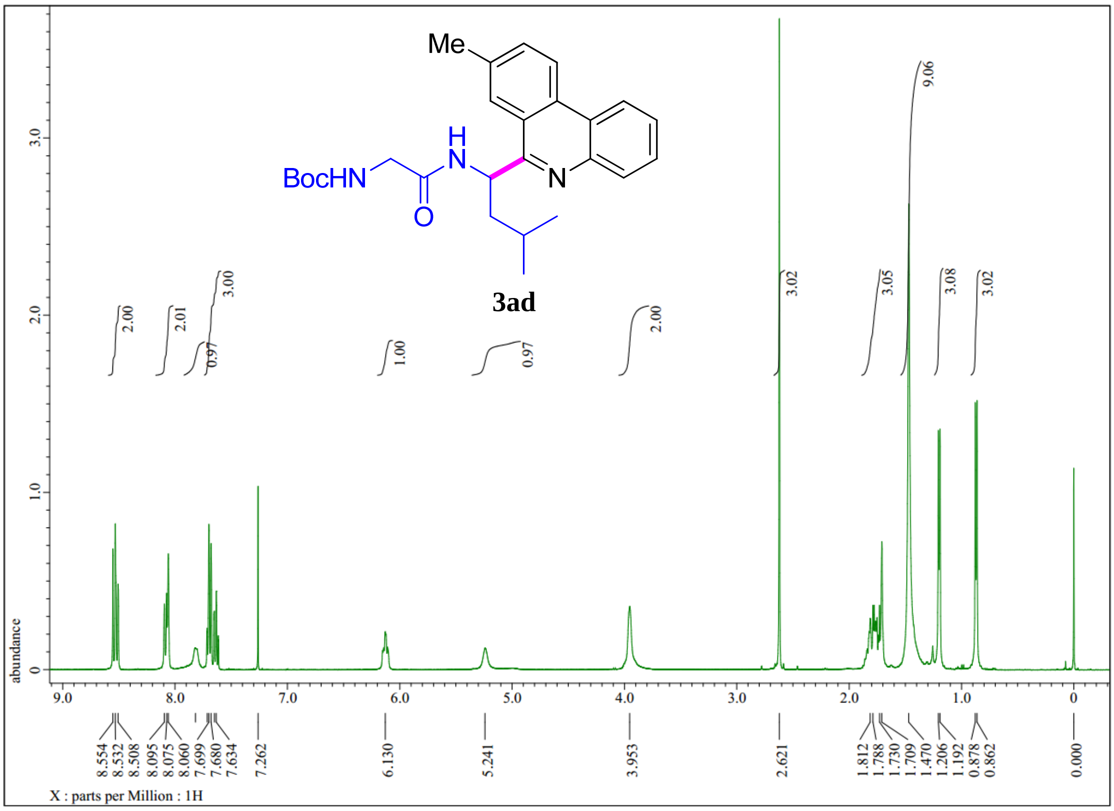

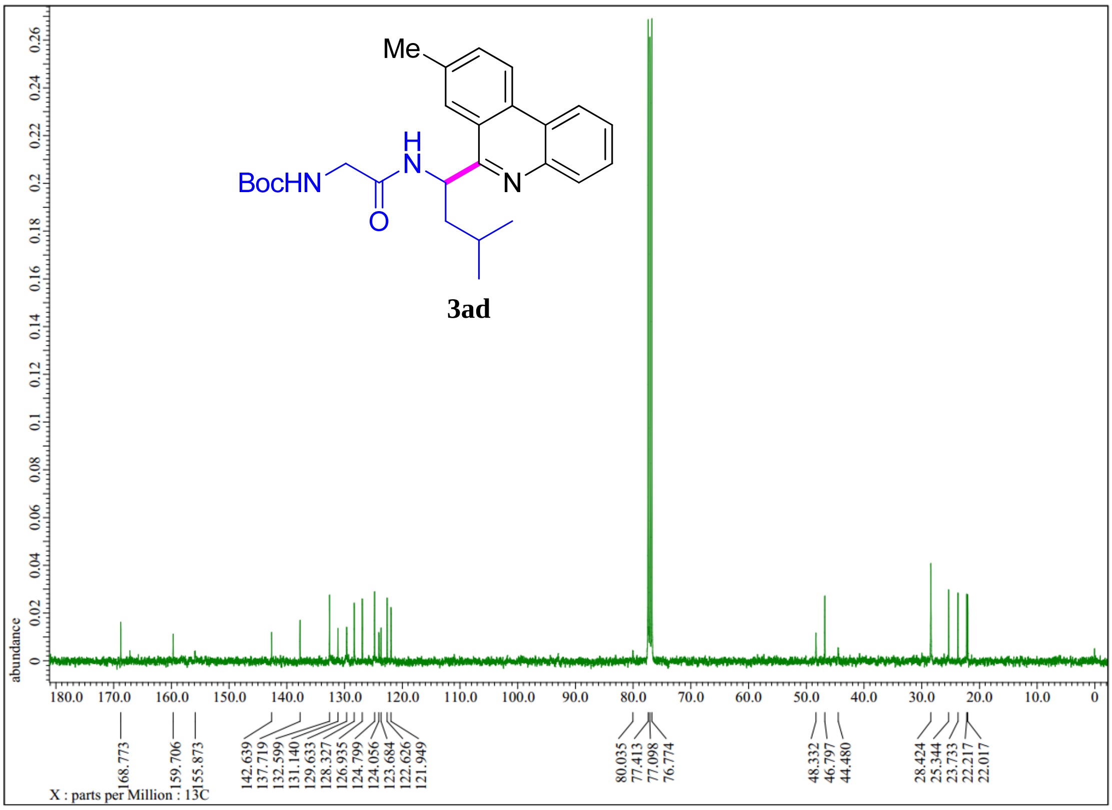

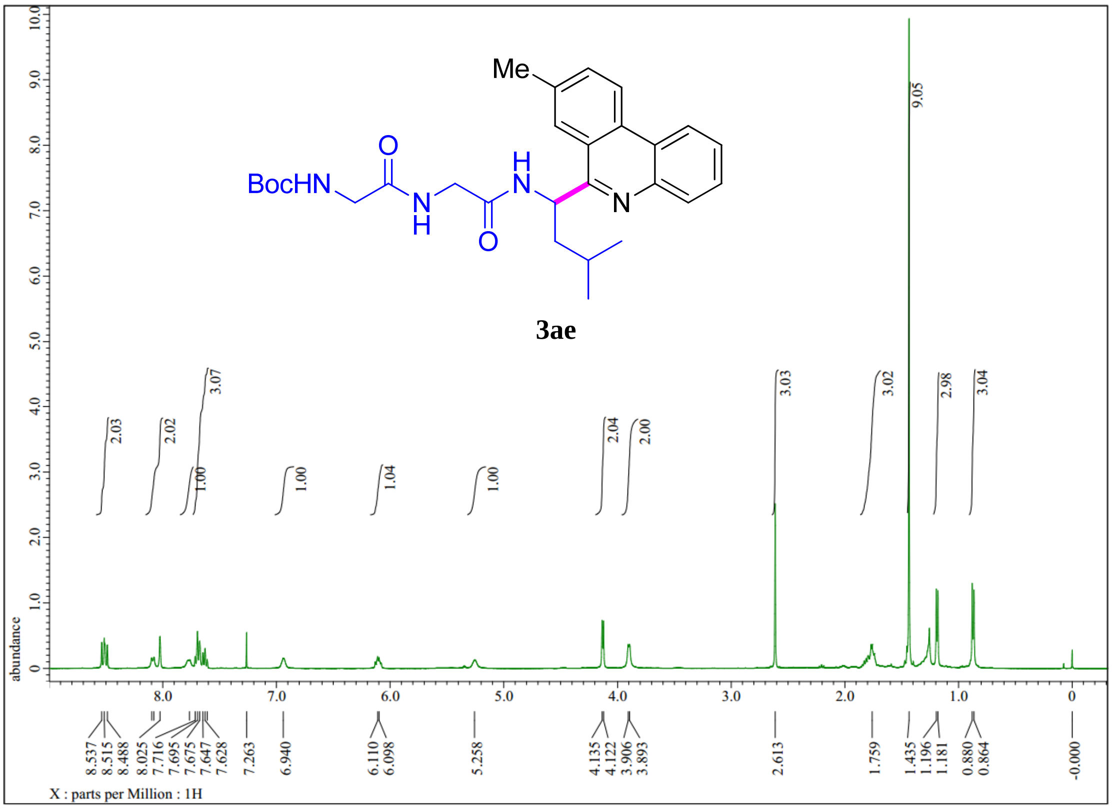

Me
BocHN
3ae
9.05
2.03
2.02
3.07
1.00
1.00
1.04
1.00
2.04
2.00
3.03
3.02
2.98
3.04
8.537
8.515
8.488
8.025
7.716
7.695
7.675
7.647
7.628
7.263
6.940
6.110
6.098
5.258
4.135
4.122
3.906
3.893
2.613
1.759
1.435
1.196
1.181
0.880
0.864
-0.000
abundance
X : parts per Million : 1H


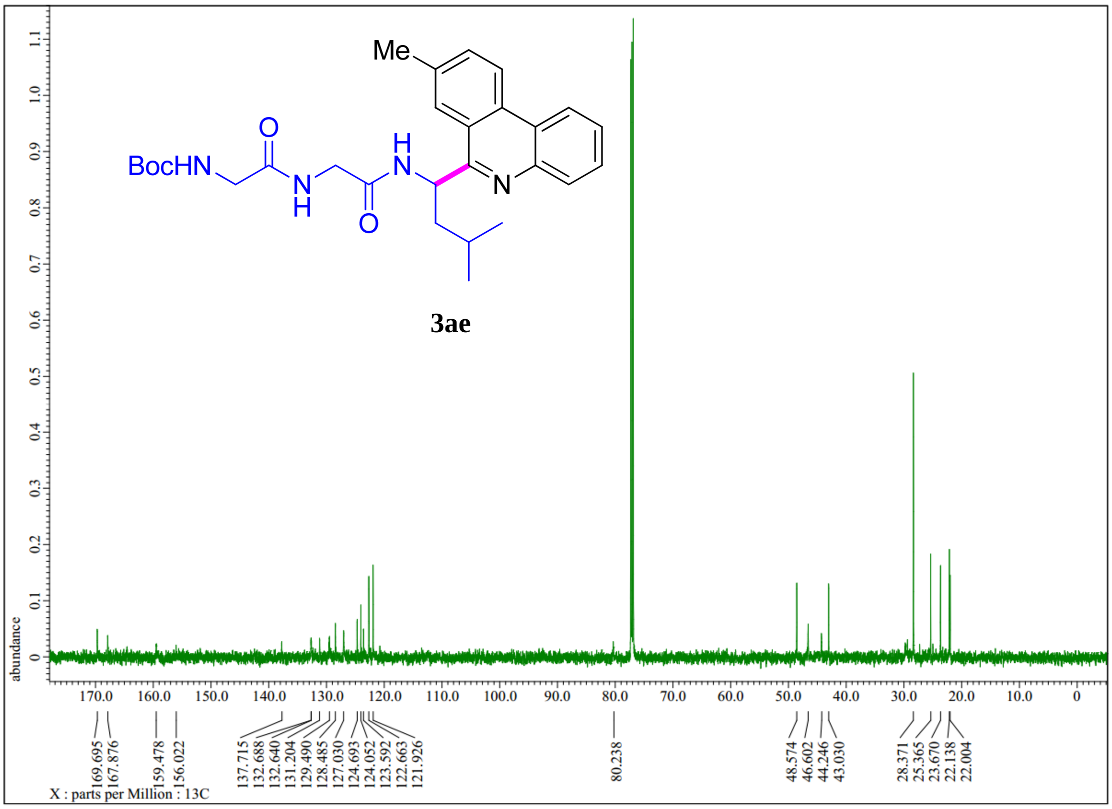

Me
BocHN
3ae
169.695
167.876
159.478
156.022
137.715
132.688
132.640
131.204
129.490
128.485
127.030
124.693
124.052
123.592
122.663
121.926
80.238
48.574
46.602
44.246
43.030
28.371
25.365
23.670
22.138
22.004
abundance
X : parts per Million : 13C

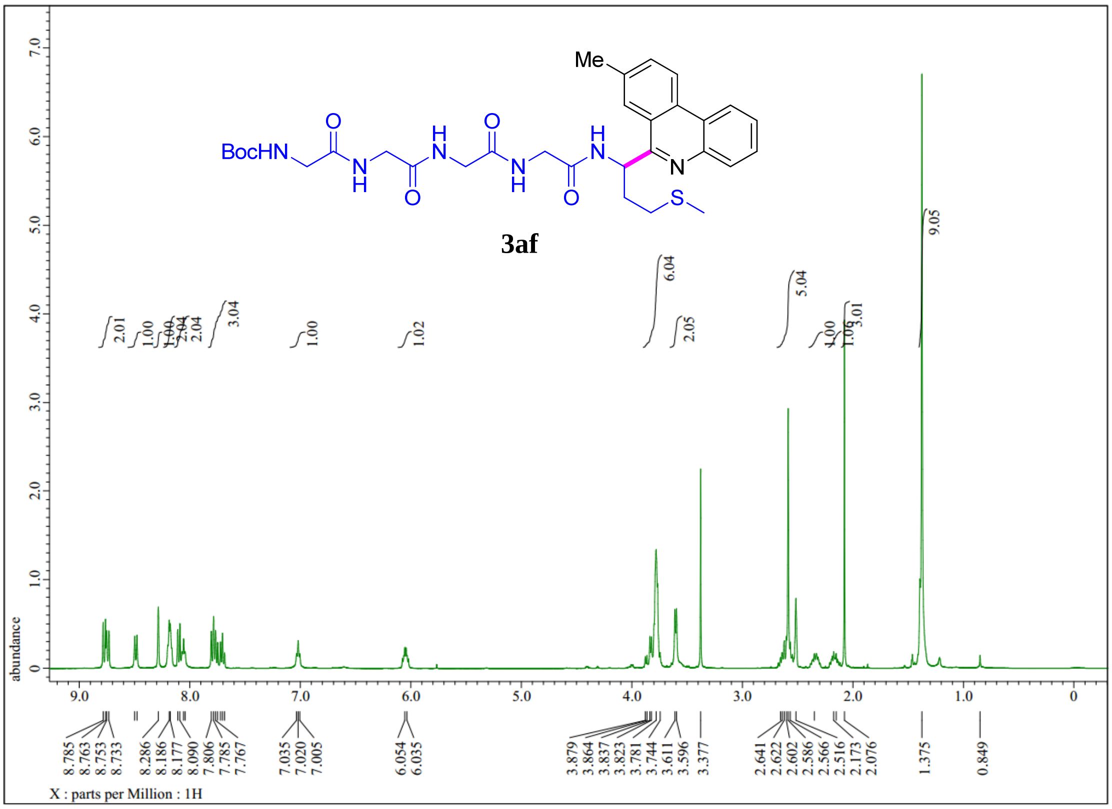

Me
BocHN
3af
abundance
X : parts per Million : 1H
8.785
8.763
8.753
8.733
8.286
8.186
8.177
8.090
7.806
7.785
7.767
7.035
7.020
7.005
6.054
6.035
3.879
3.864
3.837
3.823
3.781
3.744
3.611
3.596
3.377
2.641
2.622
2.602
2.586
2.566
2.516
2.173
2.076
1.375
0.849


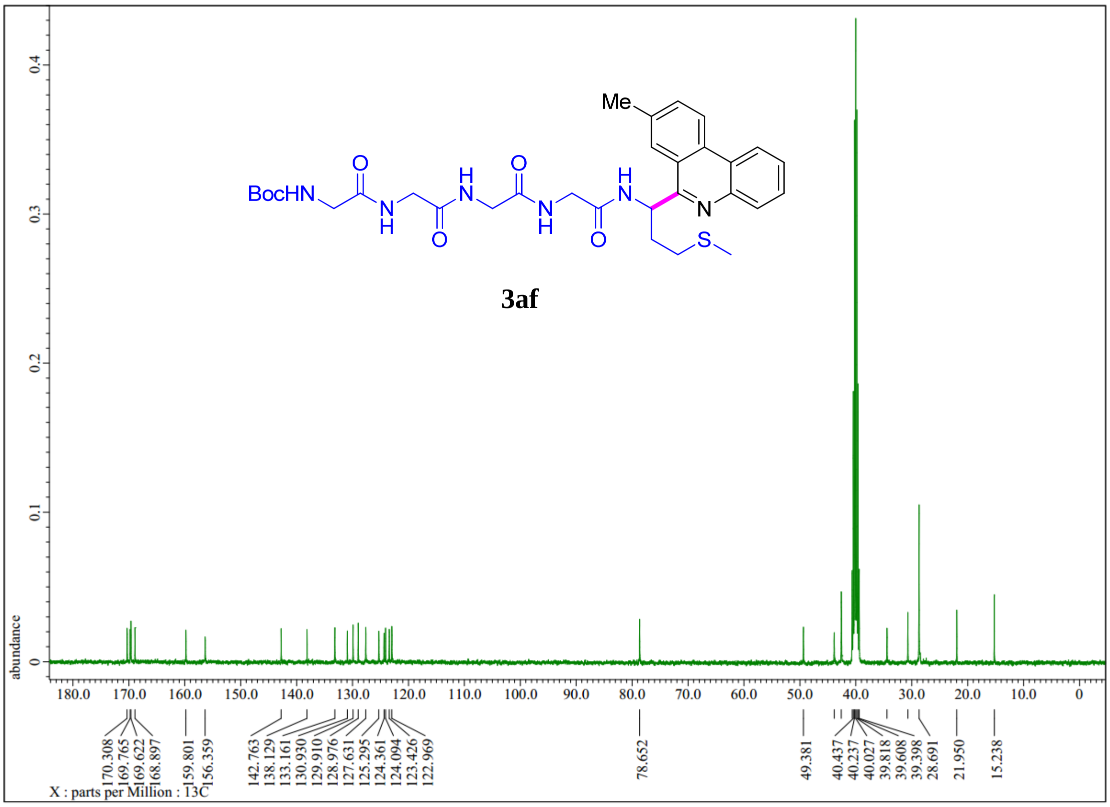

Me
BocHN
3af
abundance
X : parts per Million : 13C
170.308
169.765
169.622
168.897
159.801
156.359
142.763
138.129
133.161
130.930
129.910
128.976
127.631
125.295
124.361
124.094
123.426
122.969
78.652
49.381
40.437
40.237
40.027
39.818
39.608
39.398
28.691
21.950
15.238

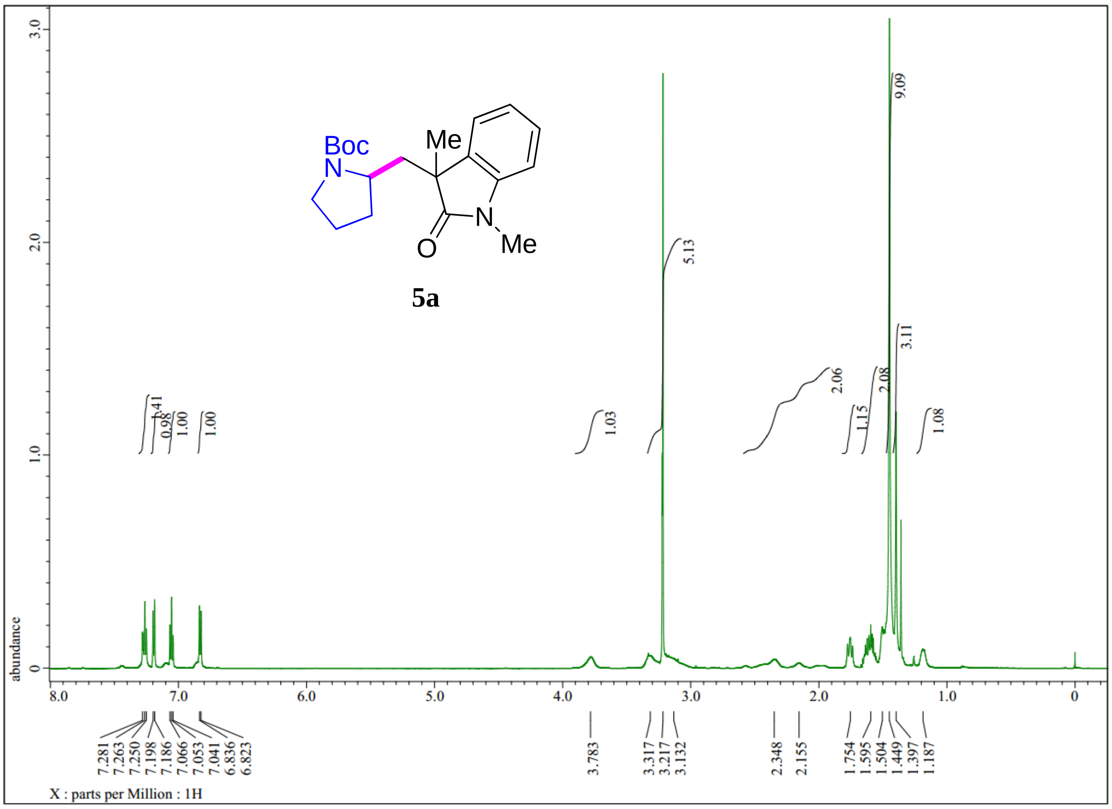

Boc
N
Me
N
Me
O
5a
5.13
9.09
3.11
2.06
2.08
1.15
1.08
1.03
1.41
0.98
1.00
1.00
abundance
X : parts per Million : 1H
7.281
7.263
7.250
7.198
7.186
7.066
7.053
7.041
6.836
6.823
3.783
3.317
3.217
3.132
2.348
2.155
1.754
1.595
1.504
1.449
1.397
1.187


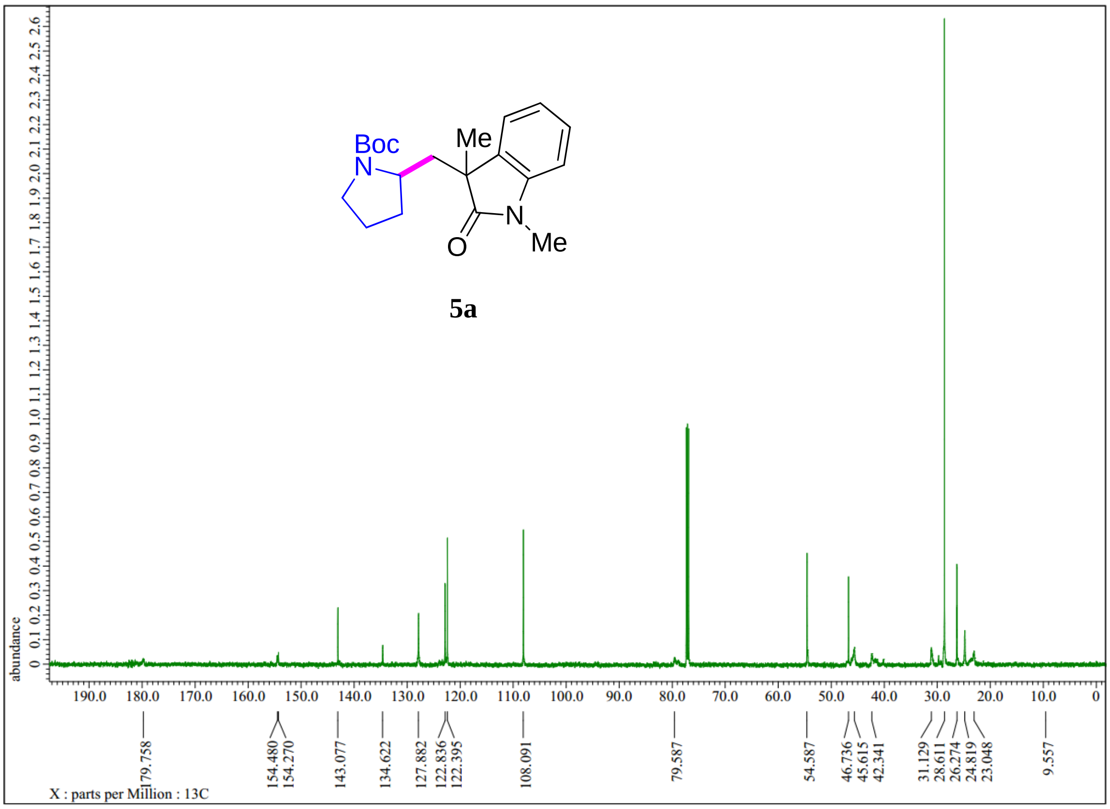

Boc
N
Me
N
Me
O
5a
abundance
X : parts per Million : 13C
179.758
154.480
154.270
143.077
134.622
127.882
122.836
122.395
108.091
79.587
54.587
46.736
45.615
42.341
31.129
28.611
26.274
24.819
23.048
9.557

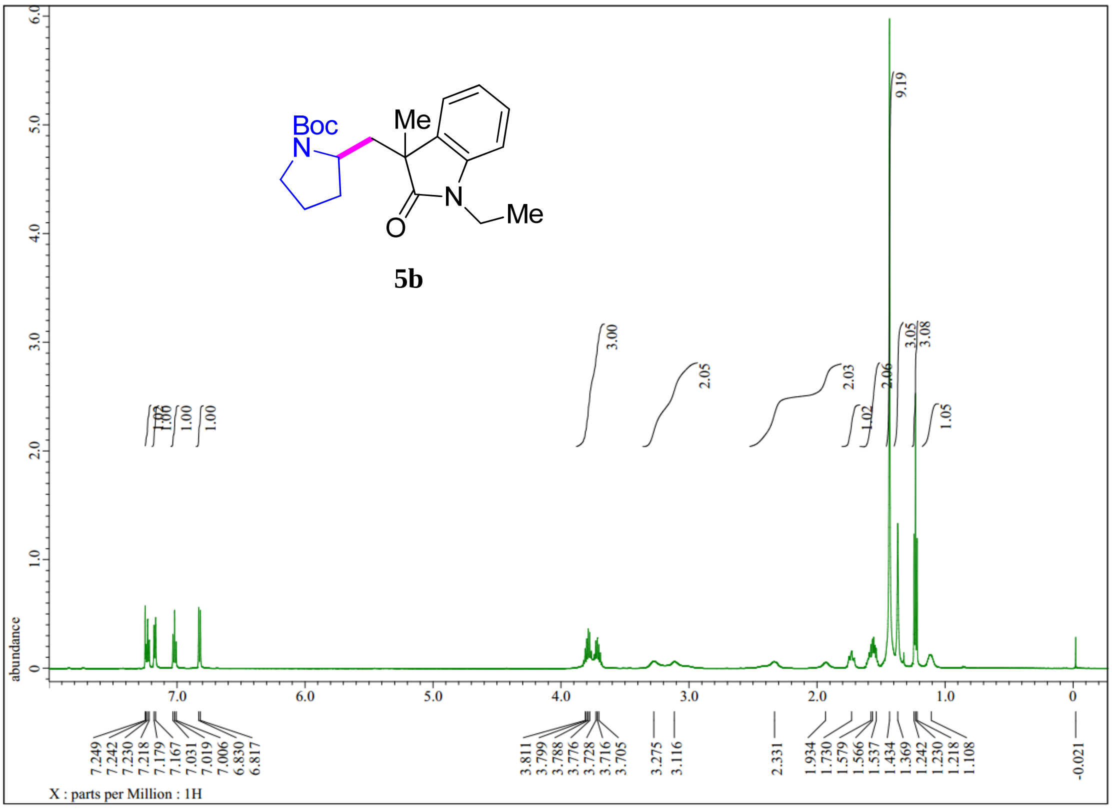

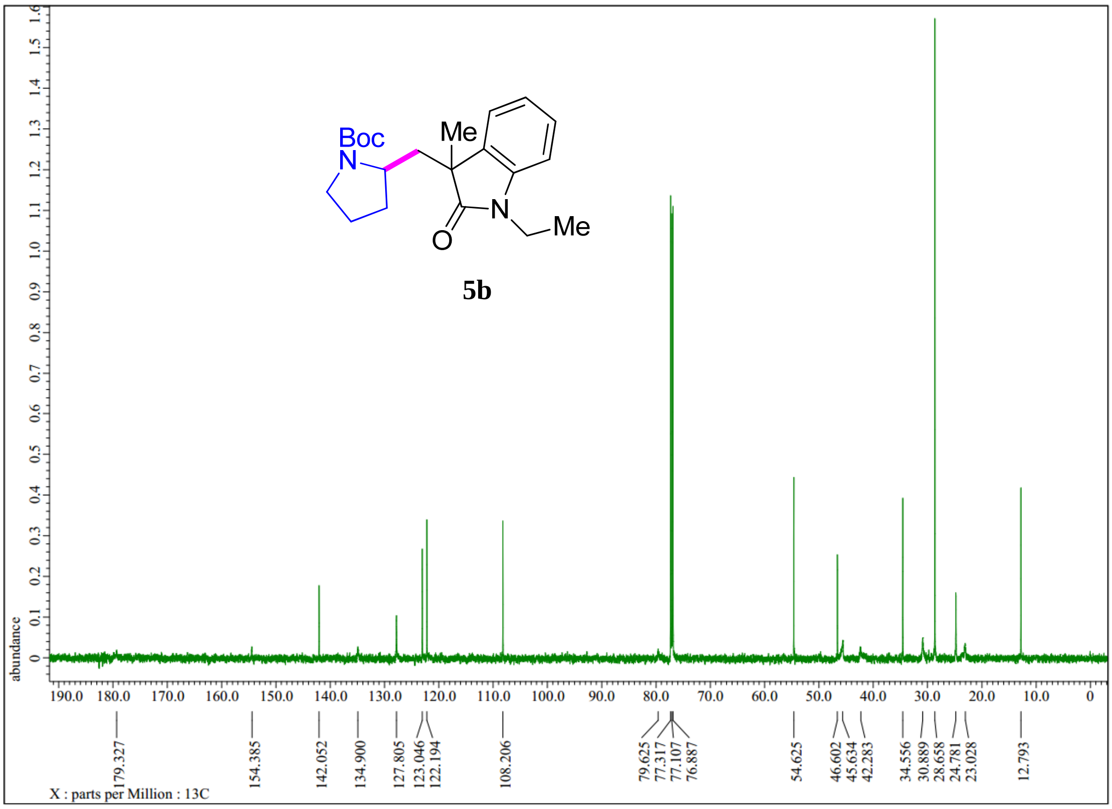

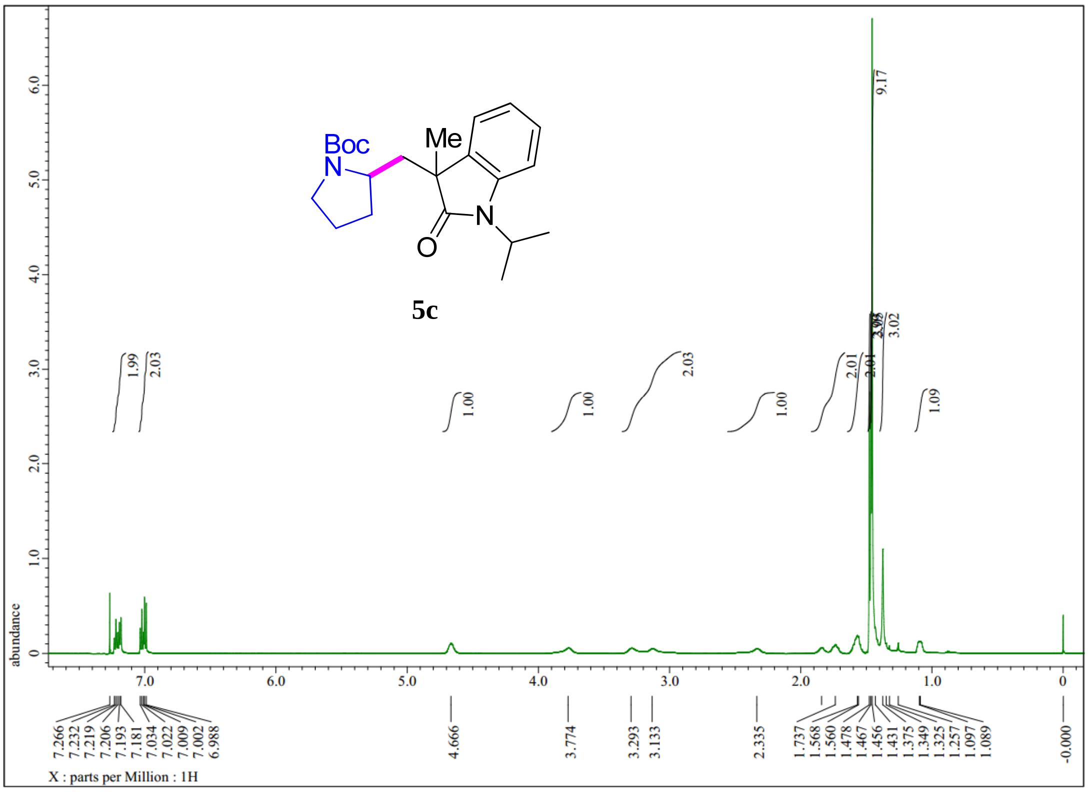
Boc
N
Me
N
O
5c
abundance
X : parts per Million : 1H
1.99
2.03
1.00
1.00
2.03
1.00
2.01
9.17
3.02
1.09
7.266
7.232
7.219
7.206
7.193
7.181
7.034
7.022
7.009
7.002
6.988
4.666
3.774
3.293
3.133
2.335
1.737
1.568
1.560
1.478
1.467
1.456
1.431
1.375
1.349
1.325
1.257
1.097
1.089
-0.000

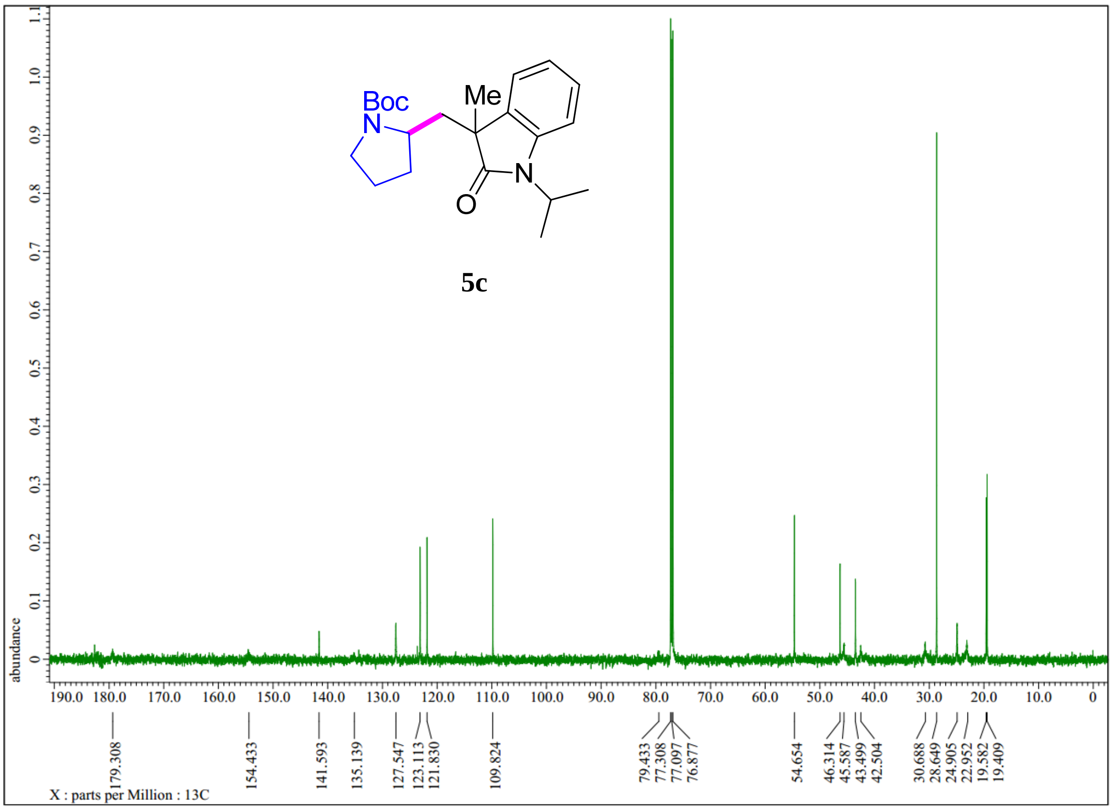
Boc
N
Me
N
O
5c
abundance
X : parts per Million : 13C
179.308
154.433
141.593
135.139
127.547
123.113
121.830
109.824
79.433
77.308
77.097
76.877
54.654
46.314
45.587
43.499
42.504
30.688
28.649
24.905
22.952
19.582
19.409

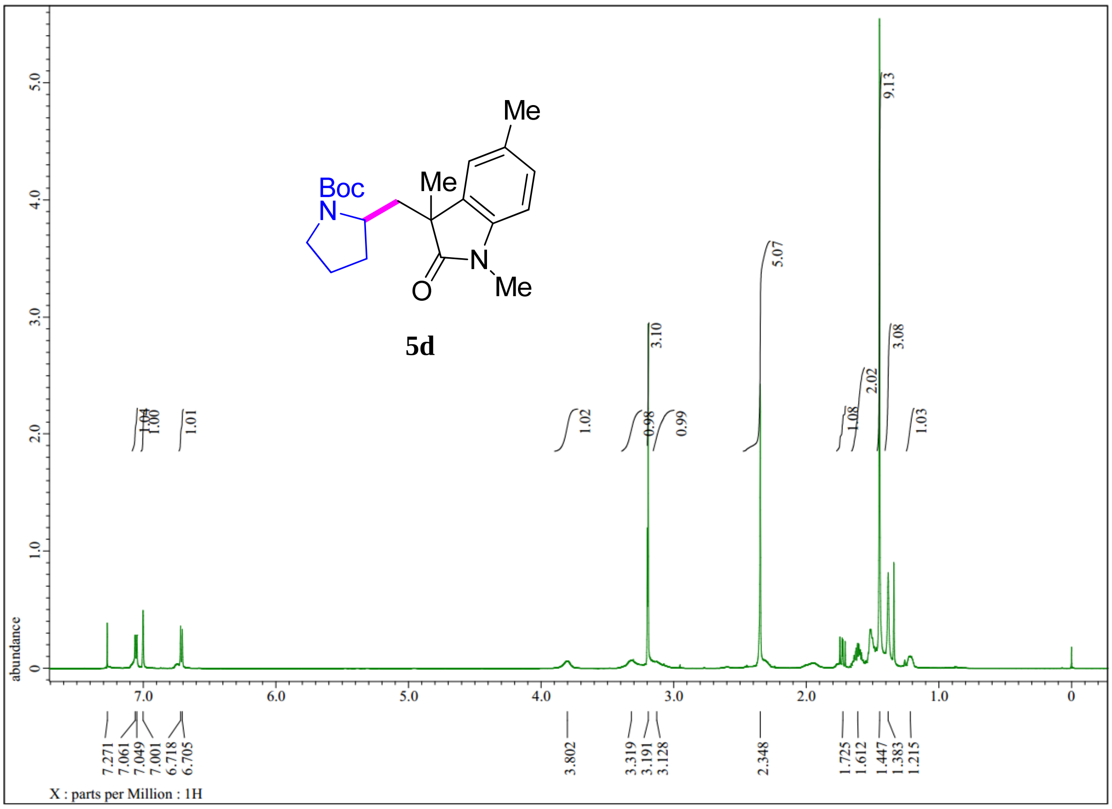
Boc
N
Me
Me
N
Me
O
5d
abundance
X : parts per Million : 1H
7.271
7.061
7.049
7.001
6.718
6.705
3.802
3.319
3.191
3.128
2.348
1.725
1.612
1.447
1.383
1.215
1.04
1.00
1.01
1.02
0.98
3.10
0.99
5.07
1.08
2.02
9.13
3.08
1.03

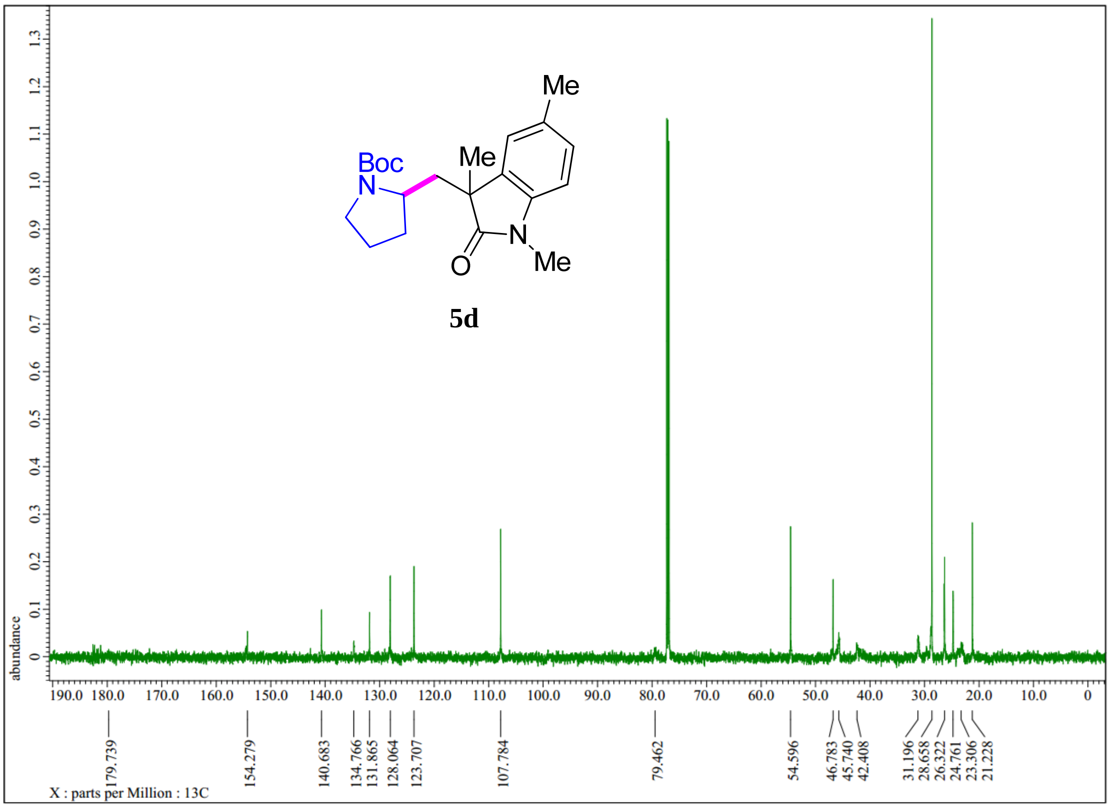
Boc
N
Me
Me
N
Me
O
5d
abundance
X : parts per Million : 13C
179.739
154.279
140.683
134.766
131.865
128.064
123.707
107.784
79.462
54.596
46.783
45.740
42.408
31.196
28.658
26.322
24.761
23.306
21.228

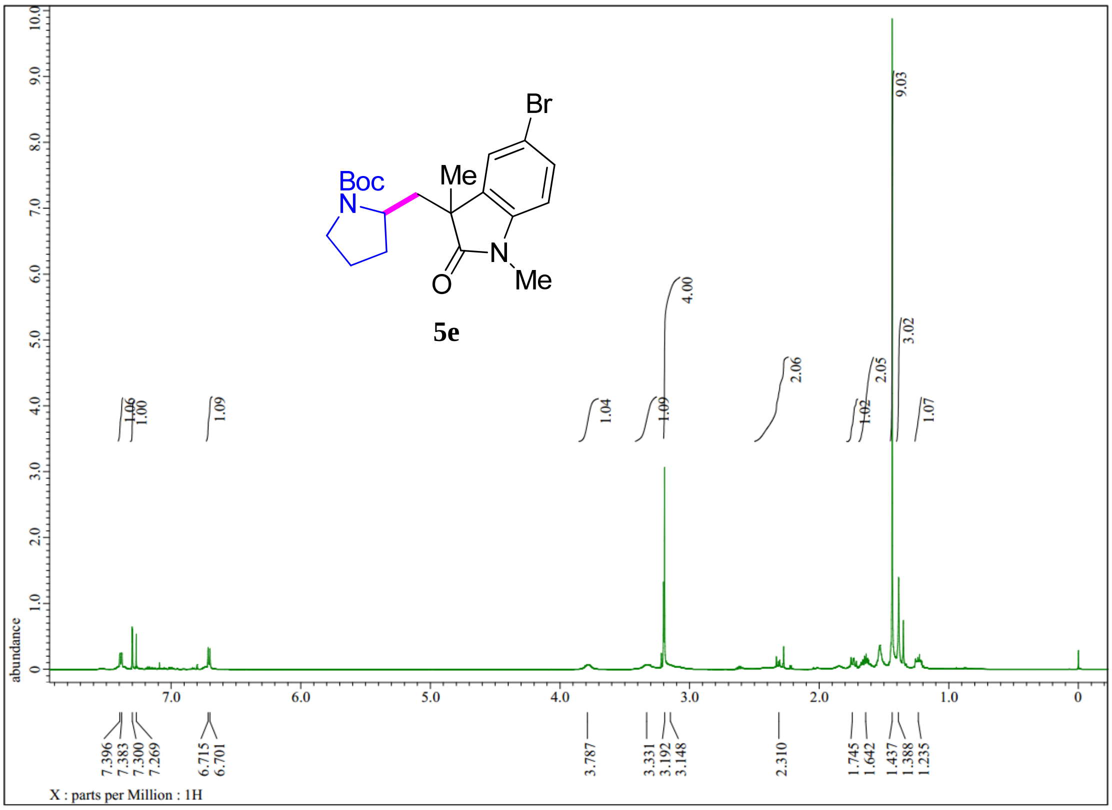
Br
Me
Boc
N
N
O
Me
5e
abundance
X : parts per Million : 1H
7.396
7.383
7.300
7.269
6.715
6.701
3.787
3.331
3.192
3.148
2.310
1.745
1.642
1.437
1.388
1.235
1.06
1.00
1.09
1.04
1.09
4.00
2.06
1.02
2.05
9.03
3.02
1.07

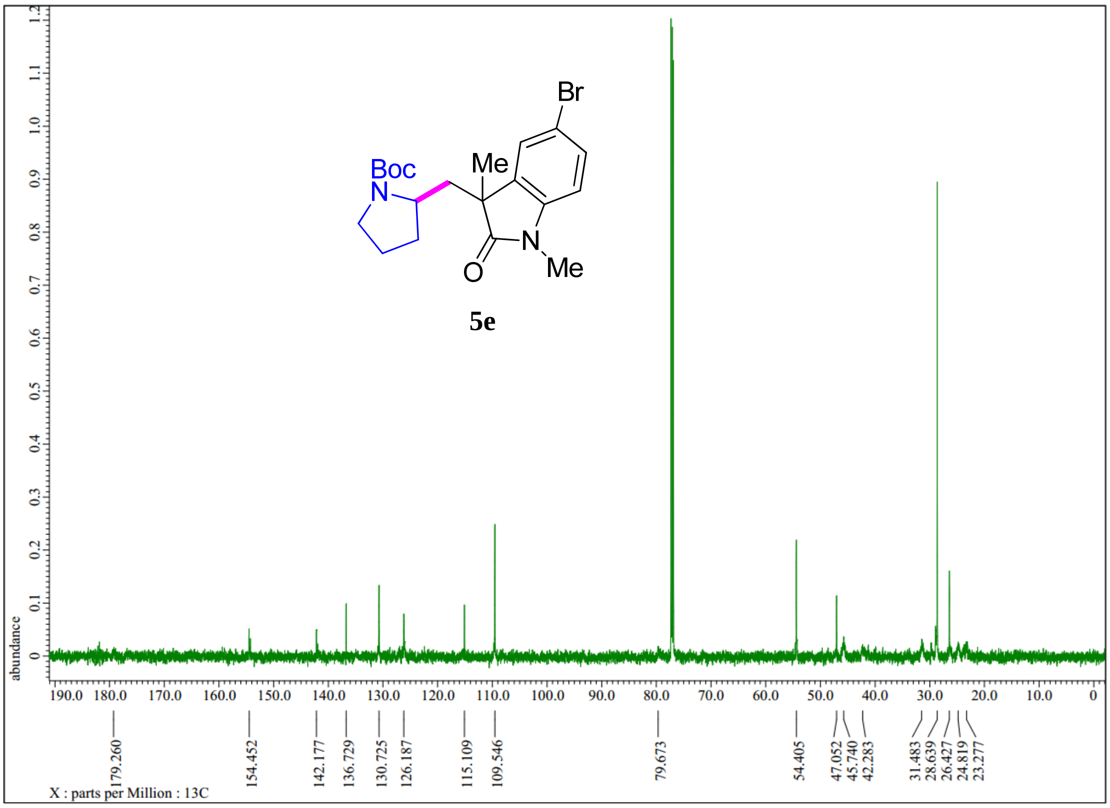
Br
Me
Boc
N
N
O
Me
5e
abundance
X : parts per Million : 13C
179.260
154.452
142.177
136.729
130.725
126.187
115.109
109.546
79.673
54.405
47.052
45.740
42.283
31.483
28.639
26.427
24.819
23.277
